# Effects and mechanisms of mUCMSCs on ovarian structure and function in naturally ageing C57 mice

**DOI:** 10.1186/s13048-021-00854-5

**Published:** 2021-10-13

**Authors:** Xing-Hua Pan, Xue-Juan Zhang, Xiang Yao, Ni-Ni Tian, Zai-Ling Yang, Kai Wang, Xiang-Qing Zhu, Jing Zhao, Jie He, Xue-Min Cai, Rong-Qing Pang, Guang-Ping Ruan

**Affiliations:** 1 Kunming Key Laboratory of Stem Cell and Regenerative Medicine, 920th Hospital of Joint Logistics Support Force, PLA, Kunming, Yunnan Province 650032 China; 2 Stem Cells and Immune Cells Biomedical Techniques Integrated Engineering Laboratory of State and Regions, Kunming, Yunnan Province China; 3 Cell Therapy Technology Transfer Medical Key Laboratory of Yunnan Province, Kunming , Yunnan Province China

**Keywords:** Ovarian senescence, mUCMSC, Follicles, Granulosa cells, Gene expression

## Abstract

**Background:**

The ovaries are the core reproductive organs in women and are critical for maintaining normal reproductive function and endocrine system stability. An ageing C57 mouse model was used to evaluate the efficacy and mechanism of mouse umbilical cord mesenchymal stem cells (mUCMSCs) and to explore the mechanism by which mUCMSCs promote the antioxidant repair of mouse granulosa cells (mGCs).

**Results:**

Eighteen-month-old C57 mice were randomly divided into a model group and a treatment group. At the same time, 2-month-old C57 mice were established as a young group (15 mice per group). The mice in the treatment group were injected via the tail vein with GFP-labelled mUCMSCs. The ovarian volume in ageing C57 mice was decreased, and there were no follicles at any stage. After mUCMSC transplantation, the mouse ovaries increased in size, follicles at various stages were observed in the cortex, and the antral follicle counts increased. The serum E2, AMH, and INH-B levels of mice in the treatment group were significantly higher than those of mice in the model control group (*P* < 0.05). mUCMSCs downregulated the expression of the autophagy-related gene LC3b and the apoptosis-related genes Bax and Caspase-3, upregulated the expression of SOD2 and the peroxidase gene PRDX IV, and reduced apoptosis rates and reactive oxygen species (ROS) levels in granulosa cells.

**Conclusions:**

mUCMSCs play roles in promoting the repair of ageing ovaries by regulating immunity, anti-inflammatory responses and the PI3K-Akt signalling pathway.

**Supplementary Information:**

The online version contains supplementary material available at 10.1186/s13048-021-00854-5.

## Background

Female fertility decreases with age. Since the 1960s, the development of contraceptive methods, the expansion of economic wealth, the increasing education level among women, and the participation of women in the work force have prompted many women to delay family planning [1, 2]. Owing to this delay, an increasing number of women who attempt to conceive are not able to become pregnant within 12 months. Thus, many couples rely on assisted reproductive technology (ART) to achieve pregnancy, but ART compensates for the decrease in natural fertility only to a limited extent [3], and many couples are still unable to conceive after long-term infertility treatments. Female reproductive ageing is attributed primarily to age-related changes in ovarian function, which are controlled predominantly by the number of follicles present in the ovarian cortex and the gradual decrease in oocyte mass within the follicles [4]. In mammalian ovaries, fewer than 1% of follicles ovulate, whereas the remaining 99% undergo follicular atresia. Autophagy and apoptosis have been previously shown to be involved in the mechanisms regulating primordial follicular development and atresia. Our current study shows that mouse umbilical cord mesenchymal stem cells (mUCMSCs) downregulate the expression of apoptosis-related genes (Bax and Caspase-3) and upregulate the expression of SOD2 and the peroxidase gene PRDX IV while reducing the granulocyte apoptosis rate and reactive oxygen species (ROS) level.

Female mammals are endowed with a finite number of oocytes at birth, each of which is enclosed by a single layer of somatic cells (granulosa cells, GCs) in a primordial follicle. The fate of most follicles is atretic degeneration, a process that culminates in near exhaustion of the oocyte reserve at approximately the fifth decade of life in women, leading to menopause [5]. The ovaries are the core reproductive organs in women and are critical for maintaining normal reproductive function and endocrine system stability. With ageing, the ovaries also age during a period known as perimenopause; this results in a decrease in fertility and leads to increased risk of bone fractures; hot flashes; urogenital atrophy; and diseases of the cardiovascular, cerebrovascular, and neuropsychiatric systems [6–9]. These changes substantially alter the physical and mental health of women and can impose substantial financial burdens on the family and society.

Normally, ROS levels in the body are maintained in a physiological balance through signal transduction and redox regulation, but consistently high ROS levels can induce oxidative damage. Under normal conditions, a complex antioxidant defence system clears ROS and maintains the redox state in a variety of cells [10]. However, as antioxidant levels decrease during ageing, the balance between ROS production and clearance is disrupted, leading to oxidative stress. Excessive ROS can induce autophagy, a lysosome-mediated self-digestion process that maintains protein and organelle quality control. ROS-induced autophagy is presumed to increase during ageing, and oxidative stress is considered a major component of human ageing [11]. Therefore, autophagy is believed to be involved in the ageing process.

Many studies have used mesenchymal stem cells (MSCs) to treat ovarian ageing. Li et al. [12] administered human umbilical cord MSCs (hUCMSCs) to naturally ageing rats. hUCMSCs secrete hepatocyte growth factor (HGF), vascular endothelial growth factor (VEGF) and insulin-like growth factor-1 (IGF-1) and promote the expression of the above three factors, thereby improving ovarian reserve function and mediating resistance to ovarian ageing. Ding et al. [13] used human amniotic MSCs (hAMSCs) to treat naturally ageing mice and observed improved ovarian function.

MSCs have become the most attractive cell type for cell-based therapies due to their proliferative capacity, multipotency, paracrine effects and immunomodulatory properties. The collection of umbilical cord MSCs (UCMSCs) is easy, painless and not ethically restricted; moreover, because sources are abundant and the cells have low immunogenicity, they have become the first choice for many MSC applications. For example, UCMSCs have been used to treat various diseases with good results, as shown in rat models of cerebral ischaemia, Parkinson’s disease, Alzheimer’s disease, multiple sclerosis, retinal disease, autoimmune diseases, and type 1 and type 2 diabetes [14].

In the current study, C57 mUCMSCs were transplanted into naturally ageing C57 mice, and the effects were evaluated by observing mouse hair colour, ovarian index values, hormone levels, ovarian tissue structure, and antral follicle counts. GCs in ovarian tissue were sorted for Smart-seq2 single-cell transcriptome sequencing, and the expression levels of the GC transcriptome in the young control group, model control group, and treatment group were compared. Second, C57 mouse ovarian GCs (mGCs) were cultured to establish a cellular model of oxidative stress. These cells were indirectly co-cultured with mUCMSCs to detect cellular ROS and apoptosis levels and evaluate whether mUCMSCs can mitigate oxidative stress in GCs. The results provide a valuable theoretical basis for the use of UCMSCs to treat ovarian ageing and repair oxidative damage.

## Results

### Model screening and evaluation results

Compared with young mice, naturally ageing C57 mice (18 months) presented a disordered oestrous cycle and ovarian atrophy, and the ovarian parenchyma was occupied by interstitial cells. The disordered oestrous cycle manifested as a no-oestrus period that was essentially the inter-oestrous period. Alternatively, the length of each period was different from that of the normal period. No follicles at any stage were observed, and serum INH-B levels were reduced (*P* < 0.05). These findings satisfied the criteria of ovarian ageing; thus, 18-month-old C57 mice were used as the mouse model of natural ovarian ageing.

### Growth and morphology of mUCMSCs

**Fig. 1:**
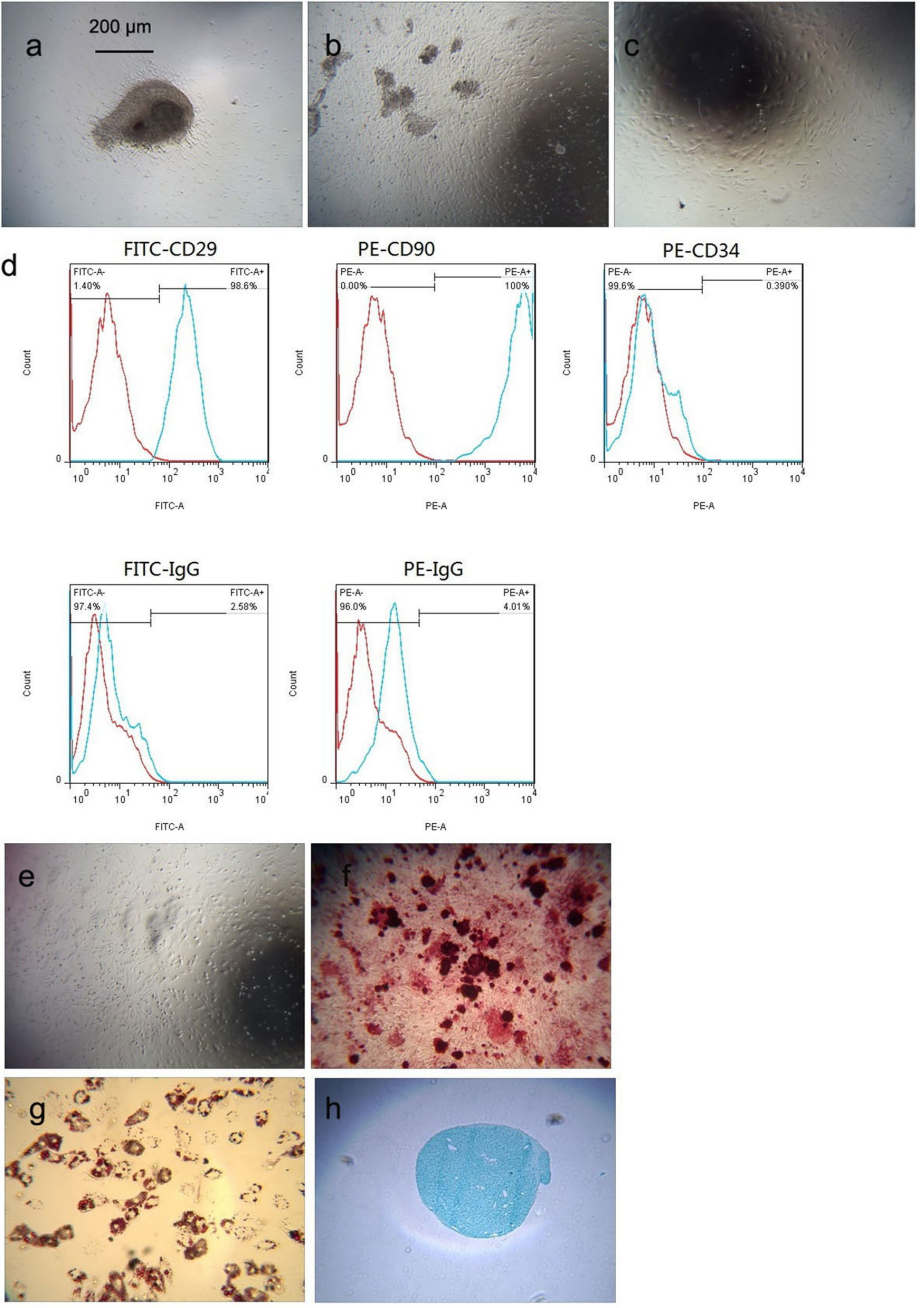
Growth and morphology of mUCMSCs (100 ×). **a** Primary cell growth and morphology on day 1 after collection. **b** Primary cell growth and morphology on day 6 after collection. **c** P4 cell growth and morphology. **d** Flow chart of mUCMSCs: the percentages of cells with positive expression for the cell surface markers CD29, CD90, and CD34 were 98.6%, 100%, and 0.39%, respectively. The FITC and PE isotype controls were negative. **e–h** mUCMSCs were induced to differentiate into osteoblasts, adipocytes and chondrocytes (100 ×) in vitro. **e** Negative control; **f** osteoblast differentiation; **g** adipogenic differentiation; and **h** cartilage differentiation

Umbilical cord tissue from mice was cut into tissue blocks, and the blocks were evenly spread in a 10 cm Petri dish. On the second day, a small number of cells around the tissue block had migrated out and displayed a short spindle-like shape (Fig. 1a). Over time, more cells appeared in colonies around the tissue mass, eventually covering 80% of the bottom of the dish (Fig. 1b), at which point the cells were passaged via digestion with trypsin for different times. A mixed cell population remained on the bottom of the culture dish. After the 3^rd^ to 5^th^ passages, the adherent cells showed spindle-like shapes and displayed a fibroblast-like and uniform morphology indicative of mUCMSCs (Fig. 1c).

### Immunophenotype of mUCMSCs

Flow cytometry showed that 98.6, 100, and 0.39% of cells expressed the mUCMSC surface antigens CD29, CD90, and CD34, respectively, while the isotype control results were negative. This expression pattern is consistent with the phenotypic characteristics of UCMSCs. The results are shown in Fig. 1d.

### Multipotent differentiation potential of mUCMSCs

Fourth-generation mUCMSCs were used for in vitro differentiation experiments, and the differentiation time was 14–21 days. The negative control is shown in Fig. 1e. After the induction of osteogenesis, Alizarin red staining was performed. Red-stained calcium nodules were present, with a positive rate of approximately 98% (Fig. 1f). After the induction of adipogenesis, staining with oil red O showed red-stained lipid droplets, with a positive rate of approximately 90% (Fig. 1g). After the induction of cartilage formation, staining with Alcian blue showed proteoglycans, as indicated by blue-stained chondrocytes, and the positive rate was approximately 95% (Fig. 1h).

### GFP labelling and tracing of mUCMSCs

**Fig. 2:**
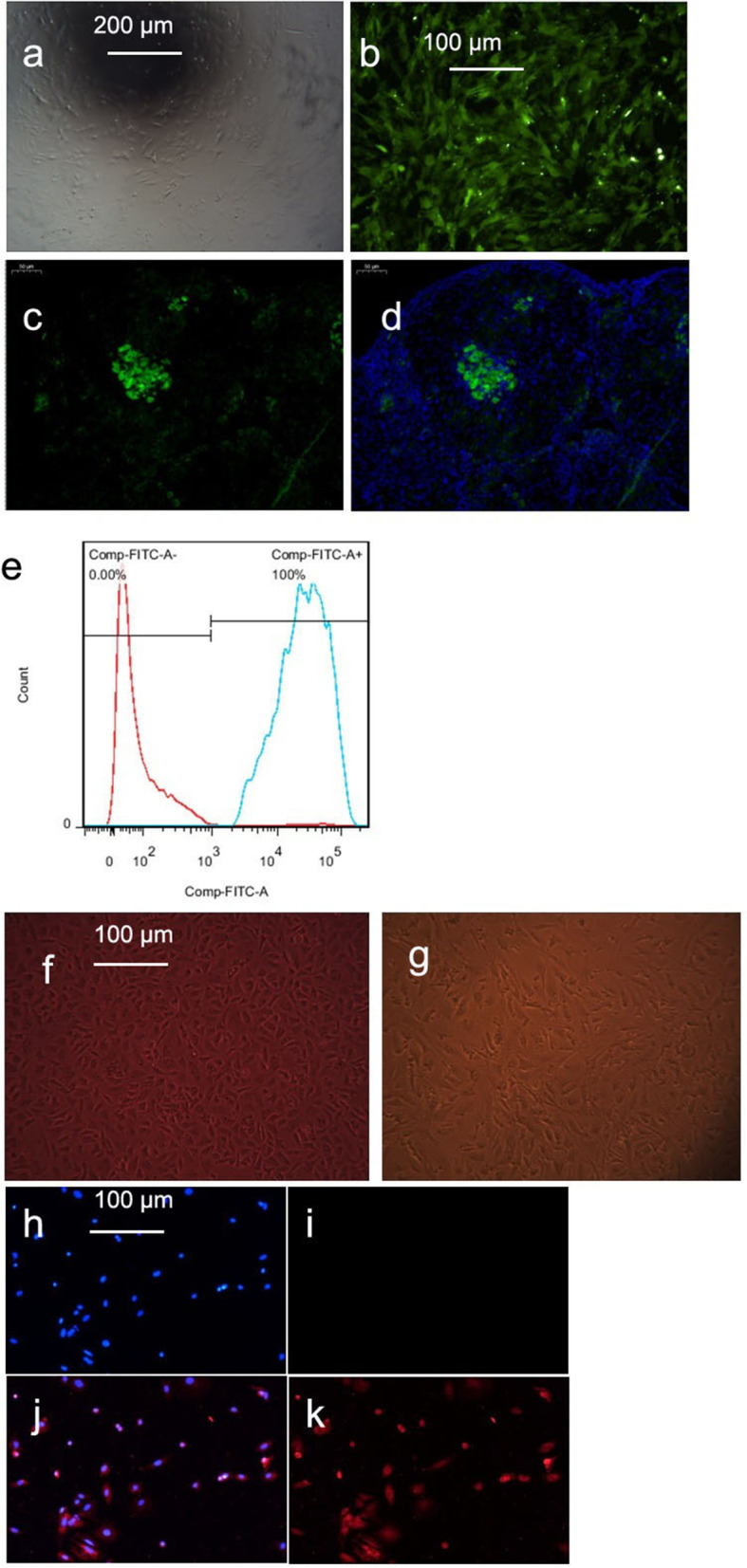
**a-d** GFP-labelled mUCMSCs (**a**: 100 × ; **b**, **c**, and **d**: 200 ×). **a** Morphology of GFP-labelled cells under an optical microscope; **b** morphology of GFP-labelled cells under a fluorescence microscope; **c** GFP-labelled cells in ovarian tissue under a fluorescence microscope; **d** GFP-labelled cells in ovarian tissue under a fluorescence microscope after DAPI staining; and **e** percentage of mUCMSCs labelled with GFP. **f and g** Growth and morphology of mGCs (200 ×). **f** Primary mGCs; **g** P1-generation mGCs. **h–k** Immunofluorescence staining of mGCs (100 ×). **h** Negative DAPI staining; **i** negative Fluoromount staining; **j** DAPI and FSHR staining; **k** Fluoromount and FSHR staining

Third-generation mUCMSCs were labelled with GFP and observed under an optical microscope (see Fig. 2a). Upon excitation, most cells exhibited bright green fluorescence (see Fig. 2b), and the percentage of GFP-positive cells detected using flow cytometry was close to 100% (Fig. 2e). One month after the GFP-labelled mUCMSCs were injected into mice, cells with green fluorescence (see Fig. 2c) were observed in ovarian tissue under a fluorescence microscope. GFP-labelled mUCMSCs (Fig. 2d) were observed after DAPI staining. Approximately 5% of cells exhibited positive staining, and the cells were mainly located in the ovarian cortex, indicating that UCMSCs injected into the tail veins of mice migrated to the mouse ovaries.

### Growth, morphology, and identification of mGCs

#### Growth and morphology of mGCs

The most important structures in follicles are the GCs, which participate not only in the secretion of hormones but also in growth and development and strongly influence the development of the follicles. Therefore, adherent cultures of mGCs were established. The mGCs primarily exhibited a short fusiform morphology with a uniform size (Fig. 2f). However, after passage to the P1 generation, morphological analysis revealed cells of different sizes (Fig. 2g). GCs are difficult to culture, and passaging should not exceed 3 generations. During the experiment, the adherence of P2-generation cells was significantly reduced, and many cells died and detached. Therefore, P1-generation mGCs were used in subsequent experiments.

#### Identification of mGCs

C57 mGCs were identified using immunofluorescence staining, and the purity of the cells exceeded 90% (Fig. 2h).

### Efficacy evaluations

#### Change in mouse hair colour

**Fig. 3:**
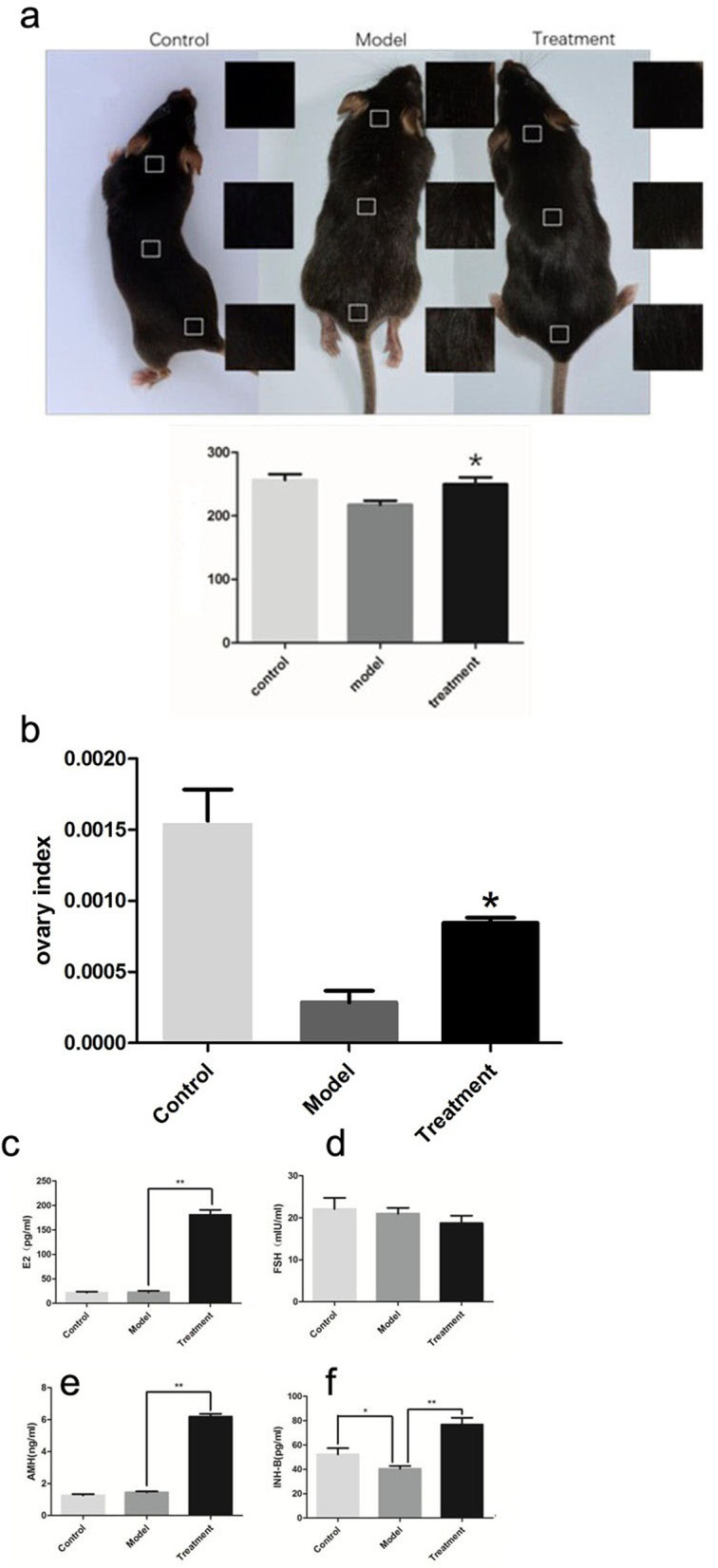
**a** Changes in the grey values of mouse hair colour: * *P* < 0.05 compared with the model group. **b** Statistical chart of the mouse ovarian index: * *P* < 0.05 compared with the model group. **c-f** Hormone levels in mouse serum. **c** Mouse serum E2 levels; **d** Mouse serum FSH levels; **e** Mouse serum AMH levels; and **f** Mouse serum INH-B levels. * *P* < 0.05 and ** *P* < 0.01

After mUCMSC transplantation, the hair colour of ageing C57 mice became darker and brighter, and the gloss was significantly improved (see Fig. 3a). ImageJ software was used to quantify the hair colour of each mouse in the three groups in the same area of the neck, back and tail based on the grey value and extent of colour loss. The degree of colour loss and the grey value of the hair colour in the treatment group were significantly higher than those in the model control group (*P* < 0.05) (Fig. 3b).

#### Mouse ovarian index

The ovarian index was calculated as the ratio of the ovarian weight to the total body weight of each mouse. Compared with the model control group, the treatment group had an increased ovarian index (*P* < 0.05) (Fig. 3c).

#### Changes in endocrine hormones

Compared with control mice, treated mice showed significantly higher serum oestradiol (E2), inhibin B (INH-B), and anti-Müllerian hormone (AMH) levels (*P* < 0.05); however, a difference in follicle-stimulating hormone (FSH) levels was not observed between groups (*P* > 0.05) (Fig. 3d).

#### Histological changes in the ovaries

**Fig. 4:**
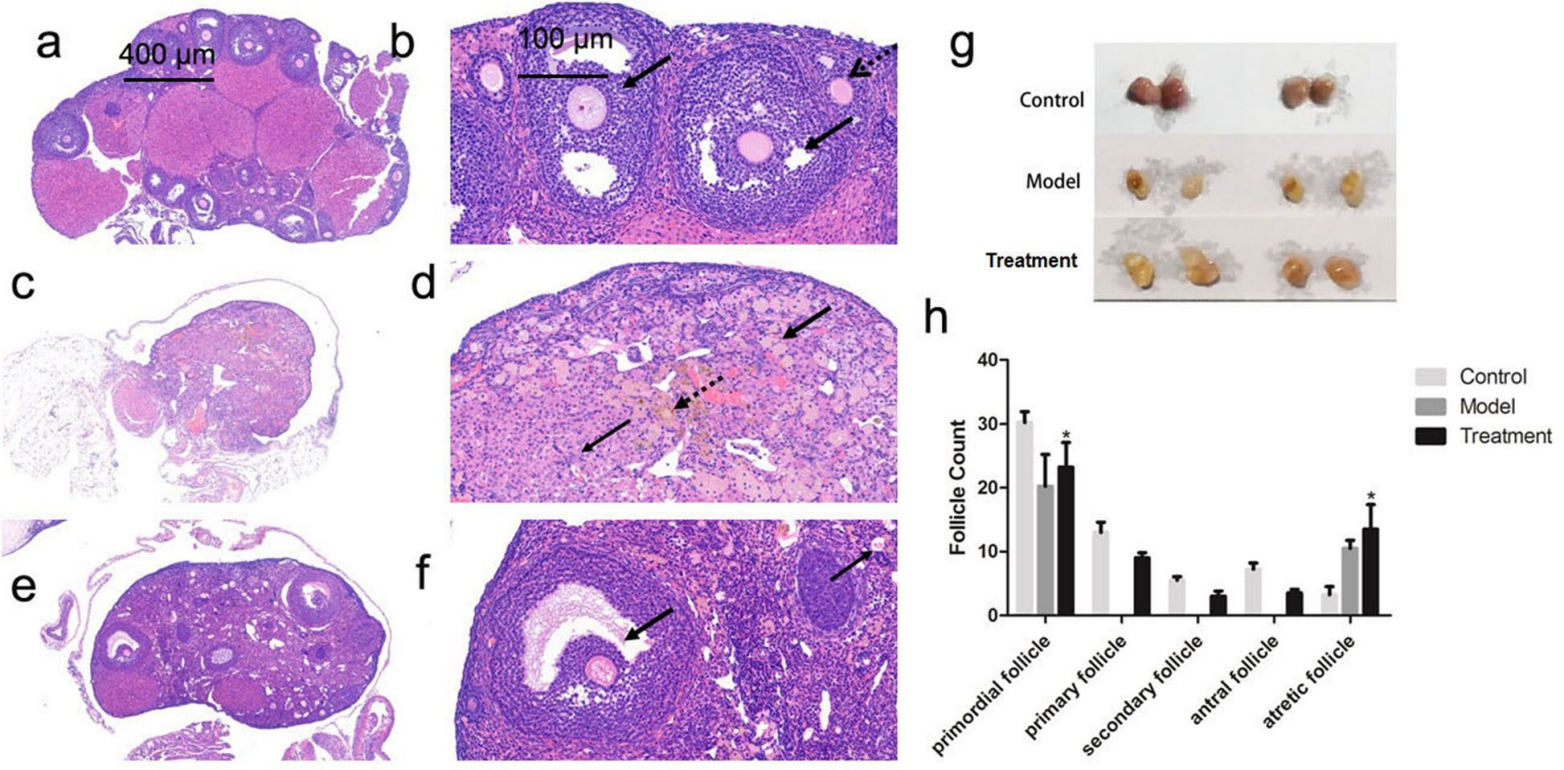
Ovarian tissue structure (**a**, **c**, and **e**: 50 × ; **b**, **d**, and **f**: 200 ×). **a** and **b** Preantral follicles (black dotted arrows) and antral follicles (thick black arrows) were visible in the young control group. **c** and **d** In the model control group, the nuclei were condensed (thick black arrow). In addition, a small number of infiltrating inflammatory cells (thin black arrow) and a small amount of brownish-yellow pigment deposition (black dotted arrow) were observed. **e** and **f** In the mUCMSC treatment group, more atretic follicles were observed, and the structures of the inferior antral follicles (thick black arrows) and atretic follicles (thin black arrows) were significantly improved. **g** Observation of ovaries from each group. **h** Numbers of follicles at various stages in each group; * *P* < 0.05 compared with the model group

Based on gross observation, the ovaries of the C57 mice in the young control group were approximately 6 × 3 × 3 mm in size and pale peach in colour. The ovaries of the C57 mice in the model control group were smaller, approximately 1 × 1 × 1 mm in size, and darker, exhibiting a colour akin to reddish brown. After transplantation of mUCMSCs, the ovary size increased to approximately 4 × 2 × 2 mm, and the colour shifted to light red (see Fig. 4g). In the young control group, the ovarian structure observed under a light microscope after haematoxylin–eosin (HE) staining was intact, and the follicular structure at each level of the ovaries was clear (Fig. 4a). At higher magnification (Fig. 4b), preantral follicles and antral follicles were visible. The ovarian structure in the model control group was completely lost, and no follicular structures were observed at any stage (Fig. 4c). At high magnification (Fig. 4d), a large number of primordial follicles and GCs were swollen, necrotic and disintegrating, and the nuclei were condensed. In addition, small numbers of infiltrating inflammatory cells and small amounts of brownish-yellow pigment deposits were observed. After mUCMSC treatment of model mice, the ovarian structure was complete, the structure was clear, and the follicles and corpus luteum structures of all stages were visible in the ovaries. More atretic follicles were observed, and the structures of the inferior antral follicles and atretic follicles were significantly improved (Fig. 4e and f). The follicles at all stages were counted, and compared with the model control group, the treatment group presented an increase in the number of primordial follicles. The ovaries of the model control group did not contain primary follicles, secondary follicles, or antral follicles, whereas all were present in treated model mice. Finally, the treatment group exhibited more atretic follicles than the model control group (Fig. 4h).

### Changes in the expression of ovarian ageing-related proteins

#### Changes in the expression of the ageing-related proteins P53, P16, and superoxide dismutase 1 (SOD1)

**Fig. 5:**
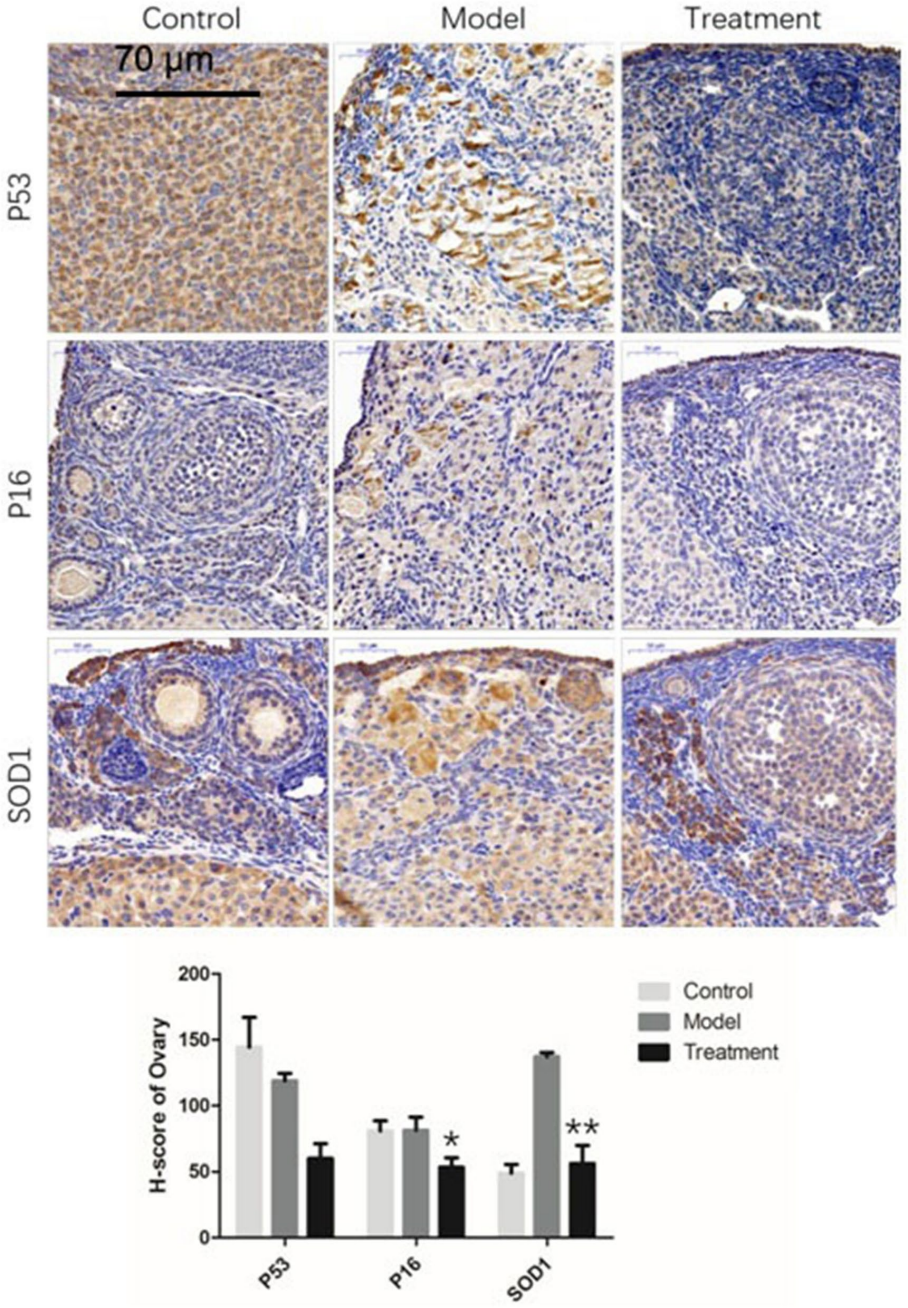
Protein staining and analysis of ageing-related gene expression in ovaries (300 ×). * *P* < 0.05 and ** *P* < 0.01

The P53 protein was expressed at higher levels in the young control group than in the model control group and the treatment group (*P* > 0.05). The P16 protein was expressed at lower levels in the treatment group than in the model control group (*P* < 0.05). The SOD1 protein was expressed at higher levels in the model control group than in the young control group, but the levels were significantly decreased in the treatment group (*P* < 0.01) (Fig. 5).

#### Changes in the expression of the autophagy-related proteins Beclin1, LC3b, Sirt1, Sirt3, and P62

**Fig. 6:**
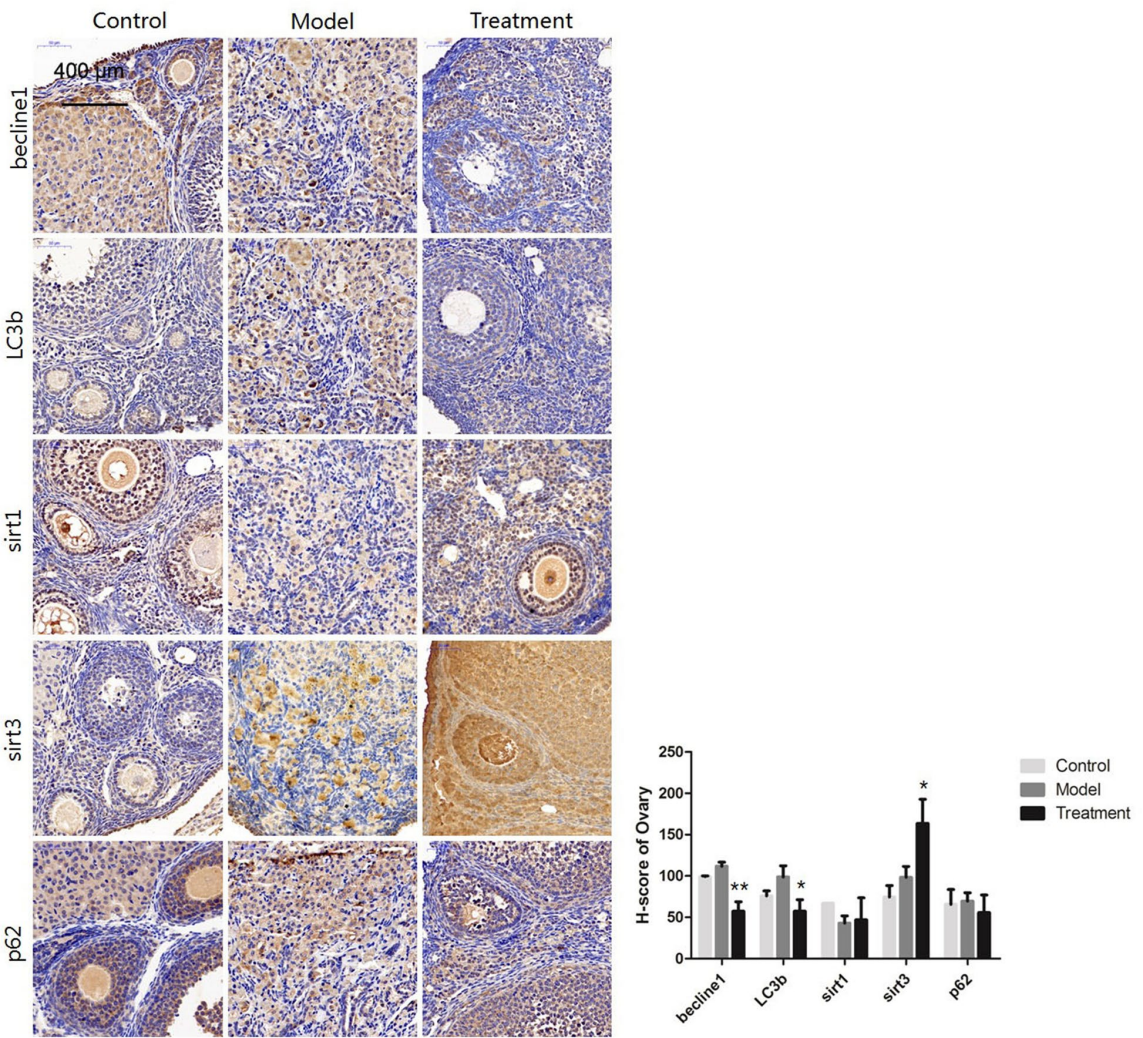
Protein staining and analysis of autophagy-related gene expression in ovaries (300 ×). * *P* < 0.05 and ** *P* < 0.01

The autophagy-related proteins Beclin1 and LC3b were expressed at lower levels in the treatment group than in the model control group (*P* < 0.05), while Sirt3 was expressed at significantly higher levels in the treatment group than in the model control group (*P* < 0.05). The protein expression of Sirt1 and P62 was not significantly different between the groups (Fig. 6).

#### Changes in the expression of the apoptosis-related proteins Bax, Bcl-2, and Caspase-3 and the GC-specific protein FSH receptor (FSHR)

**Fig. 7:**
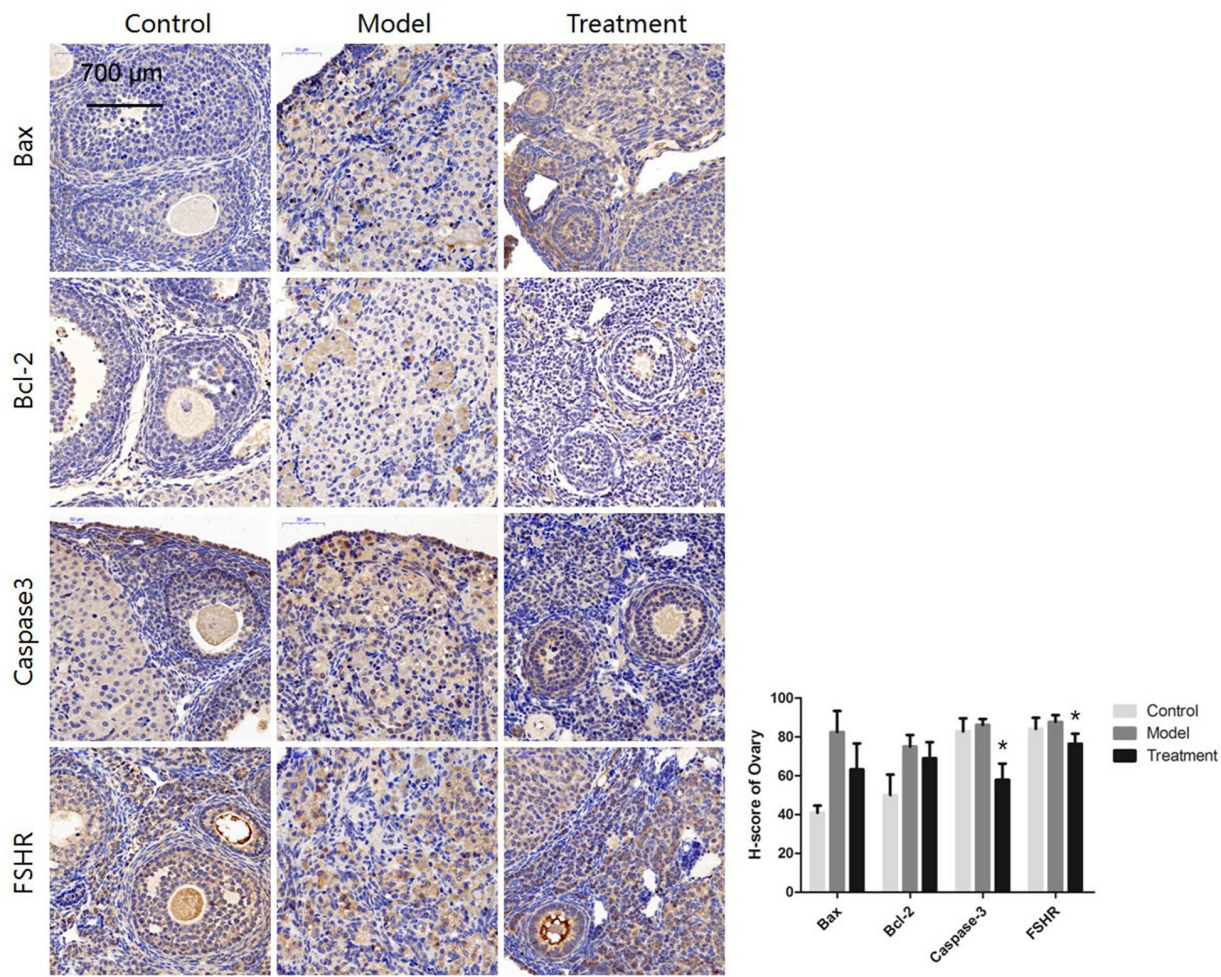
Protein staining and analysis of apoptosis-related gene expression in ovaries (300 ×). * *P* < 0.05 and ** *P* < 0.01

The expression of the apoptosis-associated protein Caspase-3 was significantly lower in the treatment group than in the model control group (*P* < 0.05), while the expression of other apoptosis-related proteins, such as Bax and Bcl-2, was not significantly different between groups. The GC-specific protein FSHR was expressed at lower levels in the treatment group than in the model control group (*P* < 0.05) (Fig. 7). FSHR is a protein specifically expressed by GCs in the ovaries, and its levels were decreased in the treatment group. It is speculated that the increase in oocytes after treatment led to a relative decrease in GCs. Notably, the young control mice exhibited caspase-3 activity equal to that of the older model mice; however, as shown in Fig. 7, the expression of apoptosis-related genes was higher in the older model group than in the young control group, and the expression levels were significantly decreased in the treatment group. Although there were no significant differences between some groups, the general trends were the same.

### Changes in ovarian cell apoptosis

**Fig. 8:**
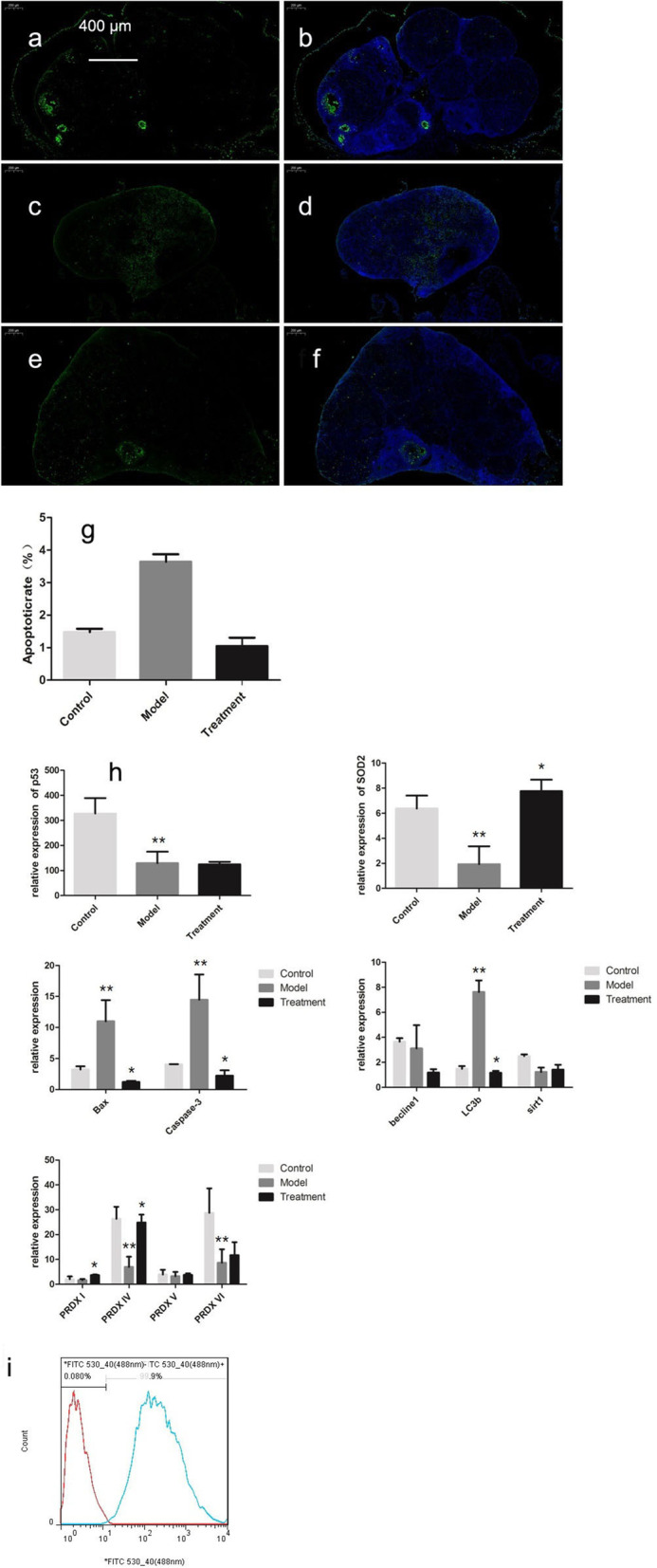
TUNEL staining of cells in ovarian tissues (50 ×). **a** and **b** Control group. **c** and **d** Model group. **e** and **f** mUCMSC treatment group. **g** Chart showing the statistical results of the positive rate (i.e., apoptosis rate); * *P* < 0.05 compared with the model group. **h** Expression of each gene in ovarian tissues. ** *P* < 0.05 between the model group and the control group; * *P* < 0.05 between the treatment group and the model group. **i** Positive rate of mGC sorting

The rate of positive TUNEL staining in the ovaries was higher in the model control group than in the young control group (*P* < 0.05) but lower in the treatment group than in the model control group (*P* < 0.05) (Fig. 8a).

### Transcriptome analysis of the expression levels of ovarian ageing-related genes

The expression of the ageing-related gene P53 was lower in the model control group than in the young control group (*P* < 0.05) (Fig. 8b). SOD2 expression was higher in both the young control group and the treated ageing group than in the model control group (*P* < 0.05) (Fig. 8c). Ovarian expression of the apoptosis-related genes Bax and Caspase-3 was higher in the model control group than in the young control group, but the ageing-related difference was attenuated in the mUCMSC treatment group (*P* < 0.05) (Fig. 8d). The autophagy-related gene LC3b was expressed at higher levels in the model control group than in the young control group, and its expression was downregulated in the treatment group (*P* < 0.05) (Fig. 8e). The expression of the peroxidase gene PRDX I was higher in the treatment group than in the model control group (*P* < 0.05). The expression of PRDX IV was decreased in ageing ovaries but upregulated in the treatment group (*P* < 0.05); PRDX VI expression was downregulated in ageing ovaries (*P* < 0.05) (Fig. 8f).

### Results of GC sequencing

#### Results of GC sorting

GC-specific FSHR antibodies were used to sort ovarian GCs via flow cytometry, with an average positive rate of 99% (Fig. 8g).

#### Results of GC sequencing

##### Differentially expressed genes in GCs

**Fig. 9:**
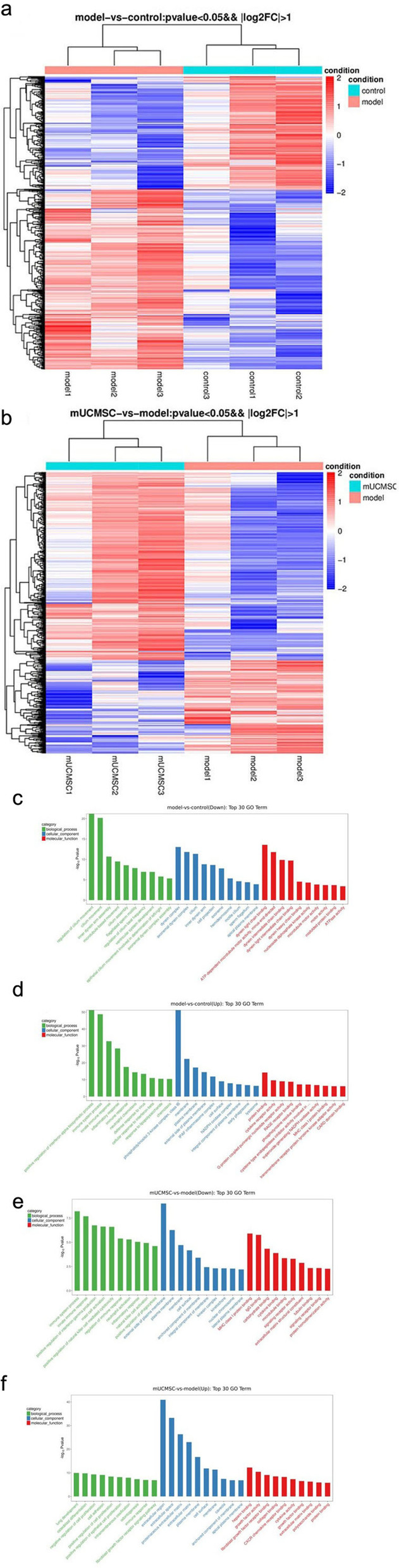
Sequencing results from GCs. **a** Heat map of the results of mRNA clustering analysis of ovarian GCs in the young control group and the model control group: Controls 1, 2, and 3 indicate mice from the young control group, and models 1, 2, and 3 indicate mice from the model control group. Red in the figure indicates protein-coding genes with high expression; blue indicates protein-coding genes with low expression. **b** Heat map of the results of mRNA clustering analysis of ovarian GCs in the mUCMSC treatment group and the model control group: Models 1, 2, and 3 indicate mice from the model control group, and mUCMSCs 1, 2, and 3 indicate mice from the mUCMSC treatment group. Red in the figure indicates protein-coding genes with high expression; blue indicates protein-coding genes with low expression. **c** and **d** GO enrichment analysis of differentially expressed genes in ovarian GCs from the young control group and model control group. **c** GO terms associated with downregulated genes; **d** GO terms associated with upregulated genes. Green indicates GO terms related to biological processes, blue indicates GO terms related to cell components, and red indicates GO terms related to molecular functions. **e** and **f** GO enrichment analysis of differentially expressed genes in ovarian GCs from the mUCMSC treatment group and the model control group. **e** GO terms associated with downregulated genes; **f** GO terms associated with upregulated genes. Green indicates GO terms related to biological processes, blue indicates GO terms related to cell components, and red indicates GO terms related to molecular functions

In all, 707 differentially expressed genes were detected in ovarian GCs between the model control group and the young control group, of which 272 were upregulated and 435 were downregulated. In addition, 832 differentially expressed genes were detected in GCs between the mUCMSC treatment group and the model control group, of which 277 genes were upregulated and 555 were downregulated. In the clustering analysis, the same types of samples appeared in the same clusters, and protein-coding genes in the same cluster were predicted to have similar biological functions. A sequencing sample cluster map was obtained based on the differentially expressed genes (Fig. 9a and b).

##### Gene Ontology (GO) enrichment analysis of differentially expressed genes in GCs

GO enrichment analysis of the top 30 genes (after screening for GO entries with more than 2 differentially expressed genes in the three classifications, the top 10 entries were selected according to the -log10(p) values in descending order) was performed, and bar graphs were constructed (as shown in Fig. 9c-d). Eleven common differences were identified (Table 1).

**Table 1:** GO analysis results for three sets of common differentially expressed genes

Term	Model vs control	mUCMSC vs model
Immune system process	upregulated	downregulated
Innate immune response	upregulated	downregulated
Immune response	upregulated	upregulated
Inflammatory response	upregulated	downregulated
External side of plasma membrane	upregulated	downregulated
MHC class I protein binding	upregulated	downregulated
Membrane	upregulated	downregulated, upregulated
Plasma membrane	upregulated	downregulated, upregulated
Cell surface	upregulated	downregulated, upregulated
Protein binding	upregulated	upregulated
Apical plasma membrane	downregulated	upregulated

##### Kyoto Encyclopedia of Genes and Genomes (KEGG) enrichment analysis of differentially expressed genes in GCs

**Fig. 10:**
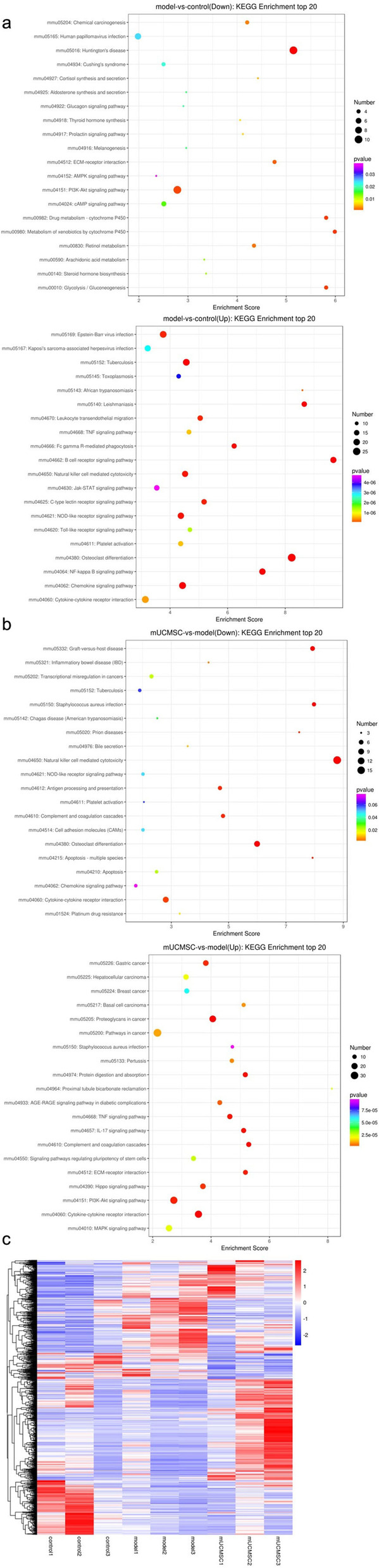
**a** KEGG pathway enrichment analysis of mRNAs identified in ovarian GCs from the young and model control groups. The X-axis in the figure represents the enrichment score, and the bubble colour changes from purple to blue to green to red depending on the *p* value; a smaller enrichment p value indicates greater significance. **b** Upregulated mRNAs in the KEGG pathway enrichment analysis of ovarian GCs from the mUCMSC treatment group and the model control group. The X-axis in the figure represents the enrichment score, and the bubble colour changes from purple to blue to green to red depending on the *p* value; a smaller enrichment p value indicates greater significance. **c** Heat map of the results of mRNA cluster analysis of ovarian GCs in the young control group, the model control group and the mUCMSC treatment group. Controls 1, 2, and 3 represent mice in the young control group; models 1, 2, and 3 represent mice from the model control group; and mUCMSCs 1, 2, and 3 represent mice from the mUCMSC treatment group. Red in the figure indicates genes with high expression; blue indicates genes with low expression

A KEGG pathway analysis of differentially expressed genes was performed to determine the pathways enriched for these genes (to determine which cellular pathways may be related to the protein-coding genes that were differentially expressed in the samples). KEGG enrichment analysis was performed for the top 20 genes after screening for pathway entries corresponding to more than 2 differentially expressed genes according to the -log10(p) values sorted in descending order. The results are shown in bubble charts (Fig. 10a and b). A larger bubble indicates a greater number of differentially expressed protein-coding genes. The colour of each bubble ranges from purple to blue to green to red according to the enrichment p value; a smaller enrichment p value indicates greater significance. Venn analysis was performed on the differentially enriched pathways between the young control group and the model control group and between the mUCMSC treatment group and the model control group, and 10 identical pathways were identified (Table 2).

**Table 2:** Common enriched pathways identified from KEGG pathway analysis

Term	Model vs control	mUCMSC vs model
Osteoclast differentiation	upregulated	downregulated
Chemokine signalling pathway	upregulated	downregulated
Tuberculosis	upregulated	downregulated
NOD-like receptor signalling pathway	upregulated	downregulated
Natural killer cell-mediated cytotoxicity	upregulated	downregulated
Platelet activation	upregulated	downregulated
Cytokine-cytokine receptor interaction	upregulated	downregulated, upregulated
TNF signalling pathway	upregulated	upregulated
PI3K-Akt signalling pathway	downregulated	upregulated
ECM-receptor interaction	downregulated	upregulated

##### Analysis of differential gene expression trends in three groups of GCs

**Fig. 11:**
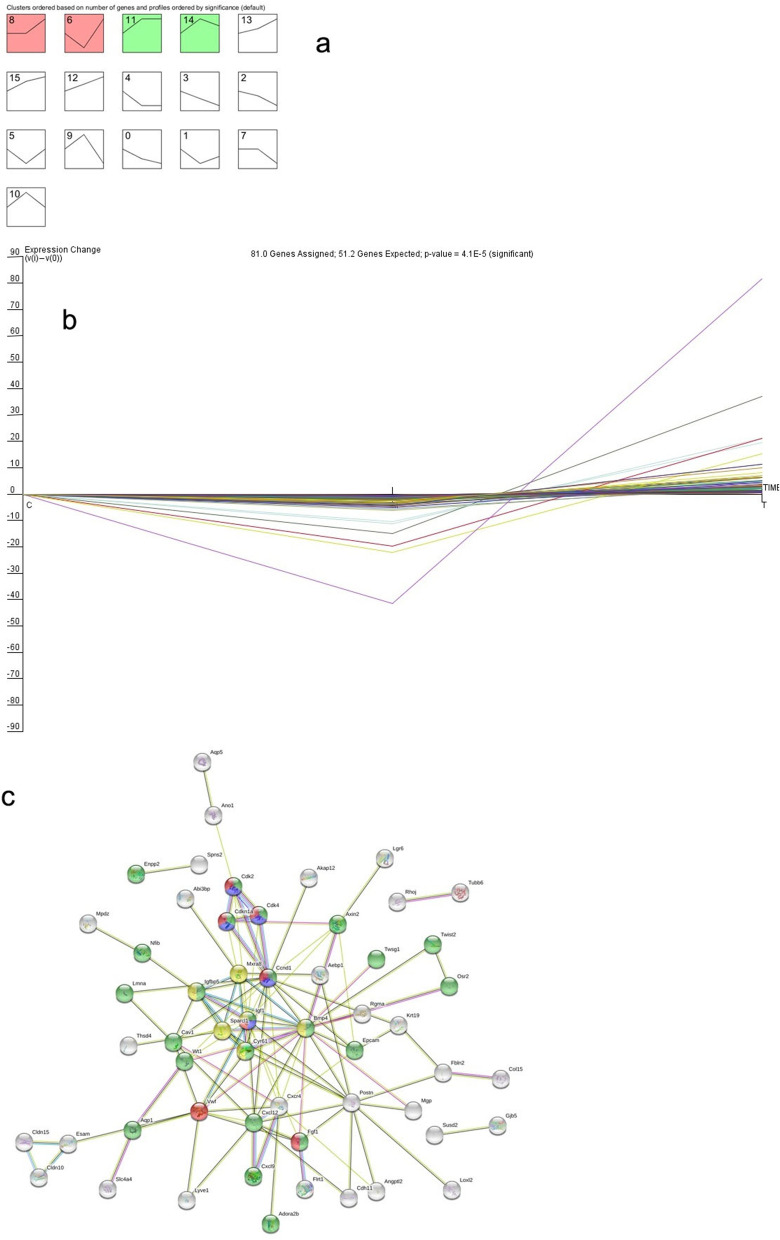
**a** STEM trend analysis of ovarian GC mRNAs in the young control group, the model control group and the mUCMSC treatment group. For trends 8, 6, 11, and 14, *P* < 0.05; for the rest without colour, *P* > 0.05. **b** Trends of 81 differentially expressed genes in the three groups. On the abscissa, A represents the young control group, B represents the model control group, and C represents the mUCMSC treatment group. **c** STRING map of the 81 differentially expressed genes. Green indicates regulation of cell proliferation, which is a biological process; blue indicates the P53 signalling pathway; red indicates the PI3k-Akt signalling pathway; and yellow indicates the IGFBP pathway regulating the transport and uptake of IGF

The same types of samples tend to group together in clustering analysis, and protein-coding genes in the same cluster may have similar biological functions. Three groups of cluster analysis heat maps (Fig. 10c) were used to compare the three groups at the same time. The differentially expressed genes were obtained, and the normalized expression levels of the differentially expressed genes were calculated for each group. The average values of the three groups were analysed with STEM software to obtain a trend chart (Fig. 11a). In trends 8 and 6, the differentially expressed genes were downregulated in the model control group compared with the young control group but upregulated in the treatment group (*P* < 0.05). In contrast, in trends 11 and 14, the differentially expressed genes were upregulated in the model control group compared with the young control group but downregulated in the treatment group (*P* < 0.05). All other trends had *p* values > 0.05. A steeper trend indicated a greater gene expression change, and the most obvious trend was trend 6. A total of 81 genes with trend 6 (as shown in Fig. 11b) were queried in the STRING database, and a network diagram of these genes was obtained (Fig. 11c). The genes were mainly involved in the regulation of cell proliferation, the P53 signalling pathway, the phosphatidylinositol kinase-serine/threonine kinase (PI3k-Akt) signalling pathway and insulin-like growth factor (IGF) binding protein (IGFBP) regulation of IGF transport and uptake.

### Analysis of antioxidant levels in mUCMSCs

#### Model of oxidative stress in mGCs

mGCs were treated with H_2_O_2_. The mGCs were placed in a hypoxic environment, and the mechanisms by which GCs protect against oxidative stress were investigated. Under hypoxic conditions, the ROS levels in the mGCs increased (*P* < 0.05).

#### ROS levels in mGCs

**Fig. 12:**
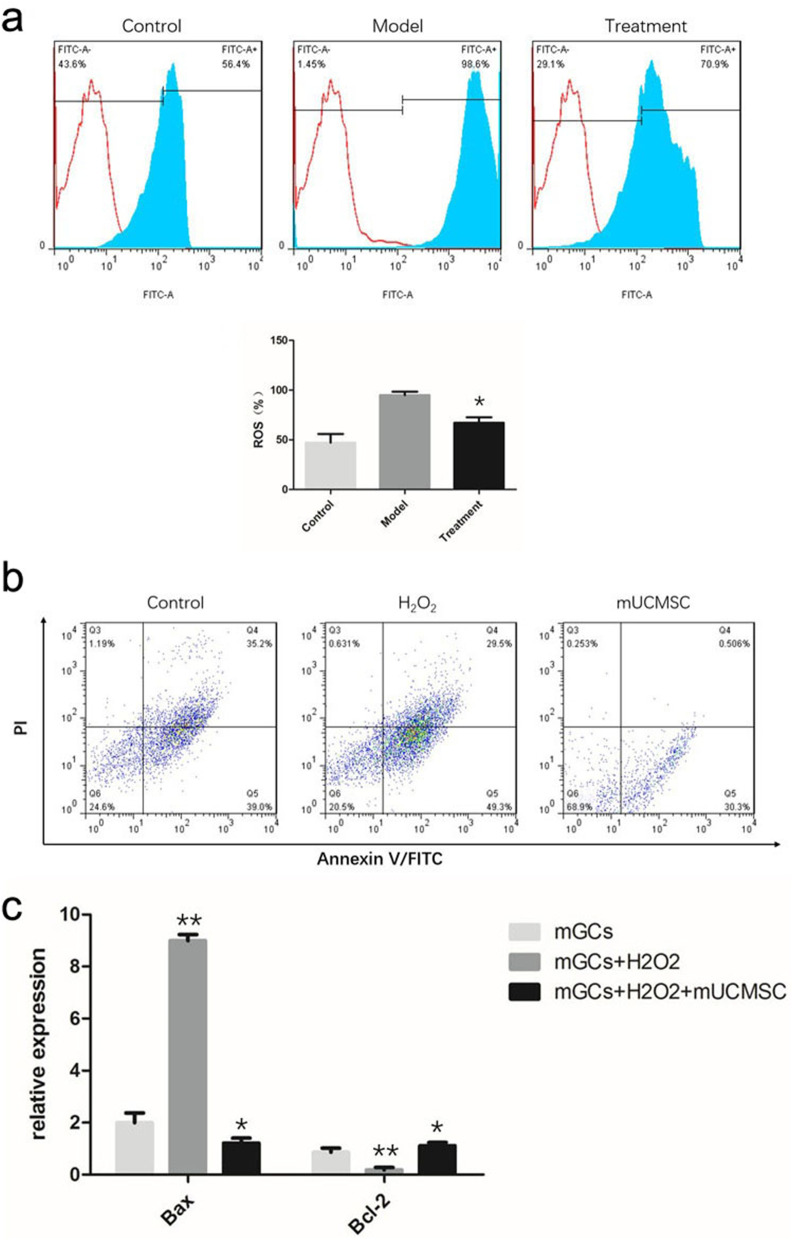
**a** ROS levels in ovarian GCs: * *P* < 0.05 compared with the model group. **b** mGC apoptosis: Q4 represents late apoptotic cells, and Q5 represents early apoptotic cells. **c** Levels of the Bax and Bcl-2 transcripts in mGCs: ** *P* < 0.05 between the model group and the control group; * *P* < 0.05 between the co-culture group and the model group

After H_2_O_2_ treatment, the ROS levels in mGCs from ageing C57 mice increased. After indirect co-culture with mUCMSCs, the ROS levels in mGCs decreased significantly (Fig. 12a).

#### mGC apoptosis

Flow cytometry was used to detect mGC apoptosis. Co-culture with mUCMSCs significantly reduced the rates of early and late apoptosis in mGCs (Fig. 12b).

#### Transcript levels of the apoptosis-related genes Bax and Bcl-2

qRT-PCR was used to detect the transcript levels of apoptosis-related genes in mGCs. Co-culture with mUCMSCs reduced the expression of the pro-apoptotic gene Bax and upregulated the expression of the antiapoptotic gene Bcl-2 (Fig. 12c).

## Discussion

### Evaluation of the ovarian ageing model

The duration of the female fertile life span is influenced by the number of oocytes stored in the ovaries as primordial follicles. Cell death, both during ovarian development in the embryo and in the postnatal ovaries, plays a critical role in determining how many primordial follicles are established and maintained within the ovaries [15]. The main functions of the ovaries are ovulation and hormone secretion. The numbers of follicles in the ovaries are limited. At 20 weeks of gestation, a female foetus will have 6 to 7 million primordial follicles; this number will gradually decrease in later years, reaching 300,000 to 400,000 by puberty and dropping to approximately 1,000 after menopause. Ultimately, the follicles are depleted by 12 to 14 years after menopause due to atresia and apoptosis [16]. On average, approximately 400–500 mature eggs are released during the life of a healthy woman; most of the follicles degenerate or are arrested at various stages of development. In addition to the decrease in the number of follicles during ageing, the quality of oocytes decreases. Recently, it was reported that bone marrow (BM) transplantation (BMT) into adult female mice conditioned a week earlier with highly cytotoxic drugs rescues ovarian function and fertility [17].

Animal models of ovarian ageing include natural ageing models and induced ageing models. Ding et al. [13] used 12- to 14-month-old female mice as ageing models. Jia Li et al. [12] used 12- to 14-month-old female SD rats to study oestrous cycles and cycle disorders. Induced ageing models employ mainly superovulation to accelerate ovarian ageing. Zhang et al. [18] induced superovulation in female mice and exposed the mice to ozone inhalation to establish ovarian ageing models. The establishment of an appropriate animal model is key to successful research. Because the present study focused on natural ageing, naturally ageing animals were used.

This laboratory primarily studies the use of UCMSCs for the treatment of ageing-related diseases. The current study successfully established and verified a C57 mouse natural ageing model according to a previous model. Female C57 mice aged 72 weeks were used as a model of ovarian ageing. In humans, the menstrual cycle proceeds regularly during the normal fertility period. When the menopausal transition period begins, the regularity of the menstrual cycle is gradually lost, which indicates that the ovaries are beginning to age. The oestrous cycle in mice is comparable to the menstrual cycle in humans. Vaginal smears were evaluated in the current study to compare the oestrous cycles of young and old mice. The oestrous cycles of old mice were indeed disordered; therefore, these mice were used as animal models of ovarian ageing.

### Collection of mUCMSCs for cell transplantation

In many studies, the stem cells used to treat ovarian ageing are hUCMSCs [12], hAMSCs [13], and human adipose-derived MSCs [19]. Chemotherapy-induced premature ovarian failure (POF) in women is currently clinically irreversible. BM MSC (BMSC) treatment is a promising cellular therapeutic strategy for POF. However, the underlying mechanism governing the efficacy of BMSCs in treating POF has not been determined. Yang et al. [20] have shown that BMSC and BMSC-derived exosome transplantation can significantly recover the oestrus cycle, increase the numbers of basal and sinus follicles in POF rats, increase E2 and AMH levels, and reduce FSH and luteinizing hormone (LH) levels in the serum. Liu et al. [21] have suggested that hAMSC transplantation can improve injured ovarian tissue structure and function in mice with oxidative damage-induced POF. hAMSCs are known to exert their effects through mechanisms related to promotion of follicular development, GC proliferation, and secretion function via improvement of the local ovarian microenvironment. Despite this knowledge, researchers have not established a mouse model or adopted the use of mUCMSCs. Because MSCs have very low immunogenicity and do not cause immune rejection, homologous MSCs can be used for treatments. However, the differences between humans and mice are obvious, and the differences in their MSCs have not been specifically quantified. mUCMSCs were used in this study to exclude effects due to species differences. As the animal model was established in C57 mice, the injected mUCMSCs were also directly collected from young C57 mice.

Young C57 mice are small and weigh approximately 19–25 g. The umbilical cords collected from pregnant mice were particularly small (approximately 1–2 cm long) and were surrounded by a membrane. Because of the umbilical cord’s size and delicate nature, blood in the umbilical cord could not be completely removed. Therefore, primary culture was initially performed to retain a portion of the tissue around the umbilical cord and remove any remaining blood. After preliminary explorations, mUCMSCs were digested with 0.25% trypsin for 1 min or less. This approach substantially reduced the number of miscellaneous cells and enabled passage to the third generation. The purity of the resulting mUCMSCs was greater than 90%. Based on morphological assessment and the outcomes of induced differentiation and flow cytometry, the mUCMSCs cultured in this study met the standards for UCMSCs and were used for cell transplantation.

### Evaluation of mUCMSC transplantation

One month after GFP-labelled mUCMSCs were injected into mice via the tail vein, cells with green fluorescence were observed in mouse ovarian tissue under a fluorescence microscope, indicating that the mUCMSCs had migrated to and settled in the mouse ovaries.

The efficacy of transplantation was evaluated from several perspectives. First, the hair colour of the mice was compared. The hair colour of the model control group was dark, but many white hairs were also present. After treatment, the hair became black and glossier, with no white hairs present. Second, gross and structural observations of ovarian tissues were performed. The results showed that the ovaries shrank and became atrophied with ageing and that the ovaries contained mainly interstitial cells. The ovarian structure was completely lost, and follicles and GCs were not observed at any stage. Swelling, necrosis, and a small number of infiltrating inflammatory cells were also present. After mUCMSC treatment, the ovarian volume became significantly larger, the ovarian structure was restored, and follicles of all stages were observed. Ovarian reserve function was also significantly improved after mUCMSC treatment. Further improvement in the reproductive ability of female mice after treatment should indicate that mUCMSCs may delay or even reverse ovarian ageing.

Third, we examined changes in the levels of hormones in mice. In addition to monitoring AMH, we evaluated ovarian function and E2, FSH, and INH-B levels. Significantly higher levels of E2 and INH-B were detected in the treatment group than in the model group, indicating that ovarian function was improved after mUCMSC treatment. Additionally, follicles of various stages, including antral follicles, appeared. GCs in the antral follicles secreted E2 and INH-B. According to experience, the FSH levels should have been increased in the old model group and decreased in the treatment group; although our results showed that FSH levels were reduced to a certain extent after treatment, the difference was not statistically significant. We speculate that the mice developed a regular oestrous cycle after the improvement in ovarian function, but the time at which the FSH levels of the mice increased during tissue collection and thus the reducing effect of mUCMSCs on FSH levels were not obvious.

### Possible mechanisms by which mUCMSCs improve ovarian function in aged mice

Numerous studies have reported that hUCMSC therapy can rescue the structure and function of injured tissues. Zhang et al. [18] explored the protective effects of hUCMSC transplantation in a model of accelerated ovarian ageing and compared 2 methods of hUCMSC transplantation: i) transplantation via intravenous (IV) injection and ii) transplantation via in situ ovarian micro-injection (MI). The results suggested that both methods of transplantation may improve ovarian function but that IV transplantation of hUCMSCs improves ovarian function and structural parameters to a significantly greater extent than MI transplantation of hUCMSCs. The two factors that affect ovarian ageing are a decrease in the number of follicles and a decrease in the quality of oocytes. The decrease in follicle number results from an acceleration of the rate of recruitment of primordial follicles and an increase in the number of atretic follicles. On the other hand, the increase in the aneuploidy of oocytes due to ageing is caused by a decrease in the mass of GCs around the oocytes.

Many studies on the treatment of ovarian ageing with MSCs have been conducted in China and other countries. Jia Li [12] treated 12- to 14-month-old SD rats with hUCMSCs and found that the hUCMSCs not only secreted HGF, VEGF and IGF-1 but also promoted the expression of these three growth factors. The results of the present study showed that the expression of the autophagy-related gene LC3b was increased in the ovarian tissue of ageing mice and decreased in ageing mice treated with mUCMSCs, indicating that autophagy was enhanced. Moreover, the ovarian tissue was recovered, autophagy was reduced, and autophagy-related gene expression was decreased in the treated mice.

Based on the immunohistochemistry and qRT-PCR results, the ovarian levels of SOD1, the autophagy-related gene LC3b, the granulocyte apoptosis-related gene Bax, and the apoptosis-related gene Caspase-3 were upregulated in the model group but downregulated in the treatment group. In contrast, the expression of SOD2 and PRDX IV was downregulated in the model group and upregulated in the treatment group. Second, TUNEL staining of ovarian tissue showed an increase in the apoptosis rate in ageing ovarian tissues, but the tissues of mice treated with mUCMSCs showed a reduced apoptosis rate. Therefore, we speculate that mUCMSCs mitigate the effects of ovarian ageing by reducing oxidative stress and ovarian cell apoptosis.

In the present study, Sirt3 expression was significantly increased in the mUCMSC treatment group; thus, we believe that Sirt3 is involved in promoting ovarian repair.

### Ovarian GC transcriptome sequencing analysis

Follicles are composed of oocytes, two somatic GCs, and follicular membrane cells surrounding the oocytes, which play a major role in steroid production. GCs proliferate and secrete steroid hormones in response to several stimuli, such as FSH and LH, and are therefore important participants in follicular development [22]. Insufficient survival signals and/or physiological/non-physiological apoptotic signals cause GCs to promote follicular atresia and apoptosis [23, 24].

Thus far, the sequencing and analysis of ovarian GCs has focused primarily on tumours; little research on the roles of these cells in ageing has been conducted. In addition, there have been no studies reporting the isolation or sequencing of mGCs.

Ovarian GCs surround oocytes; thus, the quality of GCs affects oocyte development. Therefore, this study was designed to screen genes and signalling pathways that affect ovarian GCs by comparing differentially expressed mRNAs in ovarian GCs from each group. GO enrichment analysis of the functions of differentially expressed genes revealed enrichment of genes related to the inflammatory response, immune response, cytokine and cytokine receptor activity, cell surface components, and protein binding. Compared with the young control group, the model control group showed upregulated expression levels of genes regulating the inflammatory response and immune response in ovarian GCs. In the mUCMSC-treated group, genes regulating the inflammatory response and immune response were downregulated in ovarian GCs. Consistent with the results of this study, previous research has revealed that UCMSCs possess anti-inflammatory activity, regulate immunity and can promote tissue repair through paracrine cytokine signalling. As shown in Table 1, GO analysis showed that the immune response was downregulated in the model group vs the treatment group. However, the immune system process, innate immune response, and inflammatory response terms were upregulated in the model group vs the treatment group. We conclude that UCMSCs improve the quality of ovarian GCs by exerting anti-inflammatory effects and by regulating immunity to preserve oocyte quality.

KEGG pathway analysis revealed that the enriched pathways of the differentially expressed genes included the chemokine signalling pathway, the NOD-like receptor signalling pathway, natural killer cell-mediated cytotoxicity, cytokine-cytokine receptor interactions, the TNF signalling pathway, the PI3K-Akt signalling pathway, and the ECM-receptor interaction pathway. A STEM trend analysis of the three groups identified 81 differentially expressed genes that were downregulated in old mice compared to young mice but upregulated in treated mice. The 81 genes were queried against the STRING network and were found to be associated mainly with cell proliferation regulation, the P53 signalling pathway, the PI3k-Akt signalling pathway and IGFBPs involved in IGF transport and uptake.

## Conclusions

In C57 mice conventionally raised to an age of 18 months, the ovarian volume decreased, the level of INH-B decreased, and follicles at all stages disappeared; thus, 18-month-old mice are useful models for studying ovarian ageing. Very pure populations of mUCMSCs with strong proliferation and multidirectional differentiation potential were obtained using the tissue block attachment method. In aged mice treated with mUCMSCs via transplantation, the ovarian volume was increased; follicles at all stages were visible in the cortex; the antral follicle count was increased; and serum E2, AMH, and INH-B levels were increased. All of these findings indicated that ovarian reserve function was significantly improved. Thus, mUCMSCs exert a curative effect on ovarian ageing.

## Methods

### Screening and evaluation of ovarian ageing in a C57 mouse model

Fifty female C57 mice aged 8 days or 8 months (weighing 15 ± 2.5 g or 20 ± 5 g, respectively) were purchased from Weitong Lihua (Beijing, China) and provided conventional feed until they reached the age of 2 months or 18 months, respectively.

#### Observation of the mouse oestrus cycle

Each mouse was marked via hair plucking. A baby cotton swab was dipped, and part of the cotton was removed. The mouse was fixed in a supine position with the left hand. The cotton swab head was gently inserted into the mouse’s vagina and rotated gently for 1 turn. Then, the vaginal secretions on the cotton swab head were spread evenly on a clean labelled glass slide and fixed with 4% paraformaldehyde for 30 min. A prepared melanin staining solution was dropped onto the slide, and the sample was stained for 30 min. The staining solution was then carefully washed away with distilled water, and the cells were observed with an optical microscope. The oestrus cycle phase was determined according to the following standards: pre-oestrus was indicated by the presence of numerous oval-shaped nucleated epithelial cells with a few white blood cells; oestrus was indicated by the presence of numerous anucleate keratinized epithelial cells; late oestrus was indicated by the simultaneous presence of anucleate keratinized epithelial cells, oval-shaped nucleated cells, and white blood cells; and the inter-oestrous period was indicated by the presence of numerous white blood cells and a small number of nucleated epithelial cells. The cells were collected at 09:00 every day and observed continuously for 30 days. A complete oestrus cycle in mice generally lasts for 7–11 days.

For 30 days, three vaginal smears were collected for observation of the mouse oestrus cycle, and 0.5 ml of blood was collected from 3 mice via retro-orbital sampling to measure serum E2, FSH, AMH, and INH-B levels with corresponding ELISA kits (purchased from Elisa Biotech) according to the manufacturer’s instructions. The animals were sacrificed by cervical dislocation. Paraffin-embedded sections of ovarian tissue were examined using HE staining, the ovarian structure was observed, and antral follicles were counted. The experimental protocols were approved by the Experimental Animal Ethics Committee of the 920th Hospital of the PLA Joint Logistics Support Force. All experiments were performed in accordance with relevant guidelines and regulations, including the Animal Research: Reporting of In Vivo Experiments (ARRIVE) guidelines.

### Preparation and identification of mUCMSCs

Eight-week-old female C57 mice were mated, and the gestation period of the mice was 18–22 days. Eight pregnant C57 mice were sacrificed on day 18 post conception. The umbilical cords were collected under aseptic conditions. First, the membrane on the surface of each mouse embryo was cut to find the placenta and expose the umbilical cord. The umbilical cord was clamped with tweezers before it was cut and placed into a 10 cm Petri dish containing 2 ml of saline. After all the umbilical cords were removed, the uterus and foetal mice were discarded, and fresh tweezers and scissors were used to carefully separate the umbilical cords and press out the cord blood. The cords were placed into a clean Petri dish with fresh normal saline and washed 2 to 3 times. Next, each washed umbilical cord was placed into an EP tube and aggressively cut with scissors for 3 to 5 min until the tissue pieces were approximately 1 mm^3^ in volume. The tissue was submerged in 0.5 ml of medium containing 10% FBS, mixed well, and then transferred to a 10 cm Petri dish containing 3 ml of medium to keep the tissue block from floating. After 24 h, the medium was supplemented to bring the volume to approximately 7 ml, and the medium was changed every 3 days. After the confluency reached approximately 90%, the adherent cells were digested with 0.25% trypsin, collected by centrifugation, and subcultured at a ratio of 1:3. CD29, CD34, and CD90 expression was analysed using flow cytometry. CD29-FITC and CD90-PE were purchased from American BD Company. CD34-PE was purchased from BioLegend. The mUCMSCs were induced to differentiate into osteoblasts, adipocytes and chondrocytes, and their differentiation potential was analysed by morphological observation under a microscope.

For GFP labelling of mUCMSCs, the optimal multiplicity of infection (MOI) value (referring to the number of viral particles infecting each cell) was initially determined to be 100. P3-generation mUCMSCs were seeded into the wells of a six-well plate; two wells contained coverslips that had previously been wiped with alcohol, heated with an alcohol burner, and cooled. When the confluency reached 80%, a CMV-Luciferase-EGFP-Puro lentivirus from Shanghai Jiman Biological was added along with the transfection reagent polybrene (final concentration 5 µg/ml). The virus-containing medium was changed after 8 h, and the cells were incubated for another 48 h. The coverslips were placed on a glass slide, and the cells were fixed with 4% paraformaldehyde for 30 min to observe GFP expression under a fluorescence microscope. The cells in the remaining wells were digested into a cell suspension and washed twice. The cell pellets were resuspended in PBS and divided into flow tubes (100 µl/tube), and 300 µl of PBS was added. The suspensions were run through a flow cytometer to detect the percentage of cells positive for GFP expression.

### mGC culture and identification

Primary mGCs were purchased and cultured in a cell incubator at 37 °C in an atmosphere containing 5% CO_2_ for 4 h after sterilization. Then, the complete medium filling the T25 flask was reduced by half, and the culture was continued in the incubator. On the next day, half of the solution was replaced with mGC medium (purchased from Shanghai Cybertron, PriMed-iCell-028). When the cell confluence reached 80%, the cells were digested with 0.25% trypsin (2 ml) for 1 min, and then mGC medium (4 ml) was added to terminate the digestion. The cells were collected in a 15 ml centrifuge tube and centrifuged at 300 × g for 5 min, and the pellet was resuspended in 2 ml of mGC medium. T25 culture bottles were then filled with 4 ml of mGC medium and 1 ml of the cell suspension. The cells were seeded onto chamber slides, stained with a rabbit anti-mouse FSHR antibody, observed under a fluorescence microscope, and photographed.

### Treatment with mUCMSCs

Briefly, 18-month-old C57 mice were randomly divided into a model control group and a treatment group. At the same time, 2-month-old C57 mice were established as a young control group. Each group contained 15 mice. The mice in the treatment group were injected with GFP-labelled mUCMSCs via the tail vein (1 × 10^7^ cells/kg in 100 µl, injected every Monday and Thursday for 3 consecutive weeks). The mice in the model control group were injected with the same volume of normal saline. The mice in the young control group received no treatment. One month after the final injection of mUCMSCs, the treatment efficacy and mechanism were evaluated.

### Evaluation of mUCMSC transplantation

The mouse coat colour was observed, and the ovarian index was calculated by comparing the weight of the ovaries to the total body weight. ELISAs were conducted to detect serum E2, FSH, AMH and INH-B levels. Gross dissection was performed to observe the ovarian shape and size, and HE staining was conducted to observe the ovarian tissue structure and count the antral follicles. Three mouse ovaries were collected per group, and 3 HE-stained slices were made for each ovary. The entire HE slice was scanned, the scanned slices were reviewed under a microscope to count the numbers of follicles at all levels, and statistical analysis was performed.

### Analysis of the mechanism underlying the effects of mUCMSCs

1. Immunohistochemical staining of ovarian tissue was conducted to detect the expression of senescence-related proteins (P53, P16, and SOD1), autophagy-related proteins (Beclin1, LC3b, Sirt1, Sirt3, and P62), apoptosis-related proteins (Bax, Bcl-2, and Caspase-3), and the granular cell-specific protein FSHR. After the tissue was mounted on a slide with neutral gum, it was scanned, and the resulting density data were entered into QuantCenter software. The software automatically identified and set all dark brown tissue areas as strongly positive, all brownish-yellow areas as moderately positive, all light yellow areas as weakly positive, and all blue cell nuclei as negative. Then, we identified and analysed the areas of strongly positive, moderately positive, weakly positive and negative staining (unit: pixel), calculated the percentage of positive staining for each slice and finally performed H-score scoring.

2. TUNEL staining was performed to observe apoptosis in ovarian tissues.

3. qRT-PCR detection of ageing-related genes (P53 and SOD2), autophagy-related genes (Beclin1, LC3b, and Sirt1), apoptosis-related genes (Bax, Bcl-2, and Caspase-3) and peroxidase genes (PRDX I, PRDX IV, PRDX V, and PRDX VI) was performed. Cloning and amplification primers for mouse ovarian senescence-related genes were designed and synthesized by Wuhan Saiweier Biotechnology Co., Ltd. (Table 3).

**Table 3:** Primer sequences used for the analysis of 13 ovarian ageing-related genes

Gene ID Gene name	Primer sequence (5' → 3')	Product length (bp)	Annealing temperature (°C)
NM_001127233.1 (P53)	F: ATGAACCGCCGACCTATCCT	264	60.76 59.13
	R: GCGGATCTTGAGGGTGAAATAC		
NM_013671.3 (SOD2)	F: TCCCAGACCTGCCTTACGACT	244	61.1 60.4
	R: CCCTTAGGGCTCAGGTTTGTC		
NM_009810 (CASP3)	F: GTCTGACTGGAAAGCCGAAAC	205	58.9 59.5
	R: GACTGGATGAACCACGACCC		
NM_007527 (Bax)	F: GCCTTTTTGCTACAGGGTTTCAT	151	61.9 59.9
	R: TATTGCTGTCCAGTTCATCTCCA		
NM_009741.5 (Bcl2)	F: GCTACCGTCGTGACTTCGCA	270	61.9 61.6
	R: CATCCCAGCCTCCGTTATCC		
NM_019584.3 (Beclin1)	F: GCAGCAGTTCAAAGAAGAGGTG R: TTTTGATGGAATAGGAGCCGC	122	60.3 58.7
NM_026160.4 (LC3b)	F: CGTCCTGGACAAGACCAAGTTC R: GCAAGCGCCGTCTGATTATC	80	61.12 59.77
NM_001159589.1 (sirt1)	F: CAGCATCTTGCCTGATTTGTAA R: TGGGGTATAGAACTTGGAATTAGTG	268	57.29 58.1
NM_011034.4 (PRDX I)	F: CCACGGAGATCATTGCTTTCA	139	58.64 58.55
	R: GGCCCAATCCTCCTTCTTTC		
NM_001313711.1 (PRDX IV)	F: TGTTGCGGACCGAATCTCTG R: CAATAAGGTGCTGGCTTGGAGA	178	60.39 60.62
NM_001358444.1 (PRDX V)	F: AAGAAGCAGGTTGGGAGTGTG R: AACAGCCAGGTGTAAATGCCC	201	60.48 61.17
NM_001303408.1 (PRDX VI)	F: CACCACAGGAACTGGCAGAC R: TGGTGGCAGGGTAGAGGAAG	317	61.47 60.41
NM_007393.3 (β-actin)	F: GTGACGTGACATCCGAAAGA R: GTAACAGTCCGCCTAGAAGCAC	287	58.7 61.0

4. Smart-seq2 technology was used to sequence mRNAs from mGCs, and a bioinformatics method was used to statistically analyse the sequencing data in order to identify differentially expressed protein-coding genes and pathways in the mGC transcriptome.

5. Primary mGCs were cultured to the P1 generation and stimulated with H_2_O_2_ at a concentration of 1 mmol/l for 4 h to establish an oxidative stress model. The model cells were cocultured with mUCMSCs in a Transwell chamber (with mGCs in the lower chamber and mUCMSCs in the upper chamber). mGC apoptosis and ROS levels were quantitatively measured, and the transcription levels of the apoptosis-related gene Bax and the antiapoptotic gene Bcl-2 were detected using qRT-PCR.

### Statistical analyses

All statistical analyses were performed using SPSS 21.0 statistical software. Measurement data are reported as the means ± standard deviations. The data for the three groups and the data described above were analysed using one-way ANOVA. For the analysis of differentially expressed transcripts, the negative binomial distribution test was performed to determine the significance of the read values for the differentially expressed genes, and GO and KEGG pathway analyses were performed to test the significance of the enriched differentially expressed genes using the hypergeometric distribution test. *P* < 0.05 was considered to indicate a significant difference.

### Supplementary Information

**Additional file 1.**

## Data Availability

All the data and materials are available in the manuscript.

## Abbreviations

mUCMSCs: Mouse umbilical cord mesenchymal stem cells
mGCs: Granulosa cells
ART: Assisted reproductive technology
ROS: Reactive oxygen species
hUCMSCs: Human umbilical cord mesenchymal stem cells
HGF: Hepatocyte growth factor
VEGF: Vascular endothelial growth factor
IGF-1: Insulin-like growth factor-1
hAMSCs: Human amniotic mesenchymal stem cells
MSCs: Mesenchymal stem cells
UCMSCs: Umbilical cord mesenchymal stem cells
INH-B: Inhibin B
AMH: Anti-Müllerian hormone
PCA: Principal component analysis
IGFBPs: Insulin-like growth factor binding proteins
E2: Oestradiol
FSH: Follicle-stimulating hormone
FSHR: Follicle-stimulating hormone receptor
