# Correction: Effects and mechanisms of mUCMSCs on ovarian structure and function in naturally ageing C57 mice

**DOI:** 10.1186/s13048-023-01297-w

**Published:** 2023-11-16

**Authors:** Xing‑Hua Pan, Xue‑Juan Zhang, Xiang Yao, Ni‑Ni Tian, Zai‑Ling Yang, Kai Wang, Xiang‑Qing Zhu, Jing Zhao, Jie He, Xue‑Min Cai, Rong‑Qing Pang, Guang‑Ping Ruan

**Affiliations:** 1 Kunming Key Laboratory of Stem Cell and Regenerative Medicine, 920Th Hospital of Joint Logistics Support Force, PLA, Kunming, Yunnan Province 650032 China; 2 Stem Cells and Immune Cells Biomedical Techniques Integrated Engineering Laboratory of State and Regions, Kunming, Yunnan Province China; 3 Cell Therapy Technology Transfer Medical Key Laboratory of Yunnan Province, Kunming, Yunnan Province China

**Correction: J Ovarian Res 14, 133 (2021)**

10.1186/s13048-021-00854-5

Following publication of the original article [1], the authors found an error in Fig. 6. In the original image, becline1 and lc3b of the model group are the same picture. The correct lc3b – model frame can be seen below in the corrected figure.

**Fig. 6:**
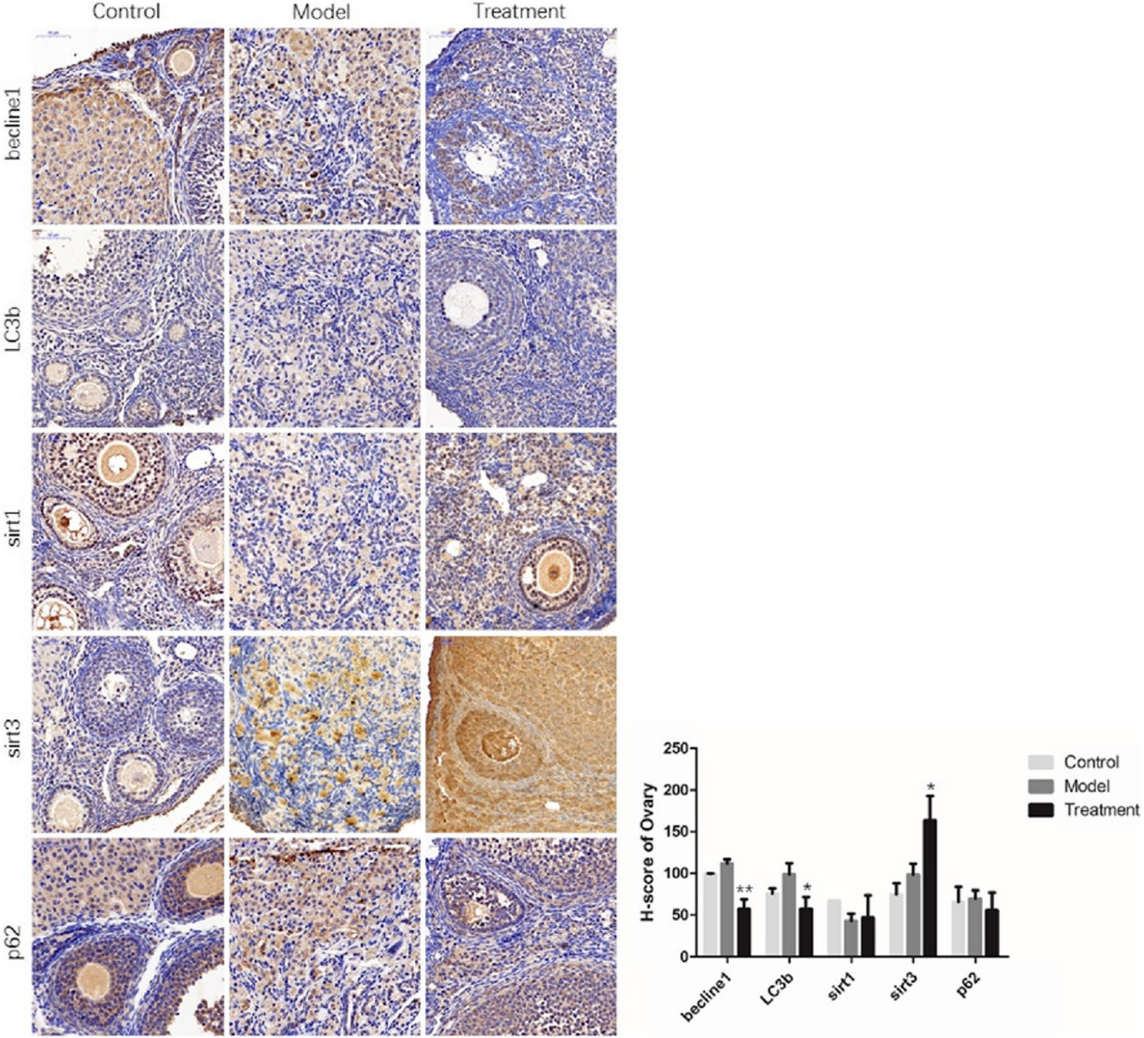
Protein staining and analysis of autophagy-related gene expression in ovaries (300 ×). * *P* < 0.05 and ** *P* < 0.01

## References

[1] Pan XH, Zhang XJ, Yao X (2021). Effects and mechanisms of mUCMSCs on ovarian structure and function in naturally ageing C57 mice. J Ovarian Res.

