# Supplementary figures and images for: Effects and mechanisms of mUCMSCs on ovarian structure and function in naturally ageing C57 mice
Source: J Ovarian Res. 2021 Oct 13;14:133. doi: 10.1186/s13048-021-00854-5 (PMC8515706; doi:10.1186/s13048-021-00854-5)

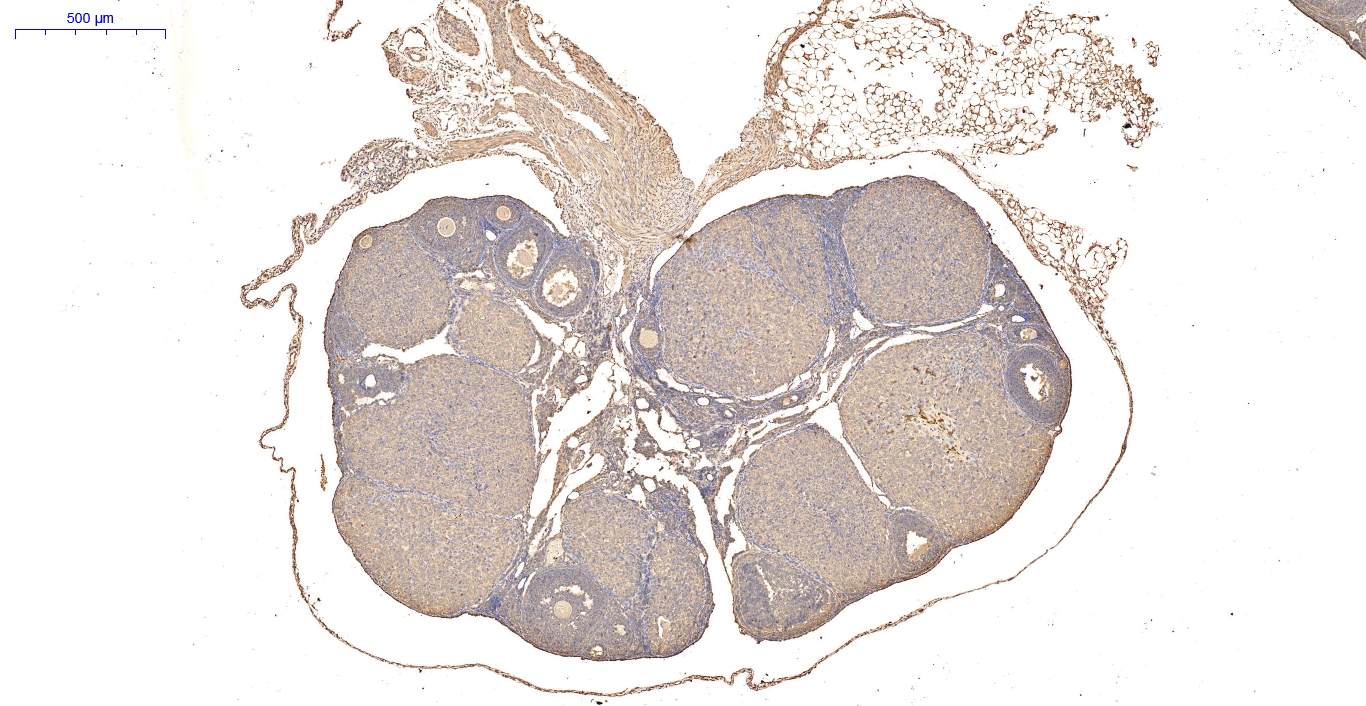

Supplement: Supplementary file 1 — Additional file 1. [file 13048_2021_854_MOESM1_ESM.zip › Supplementary Materials/Supplementary results of ovarian immunohistochemistry (whole ovarian section)/control group/Bax T_3.7x.jpg]

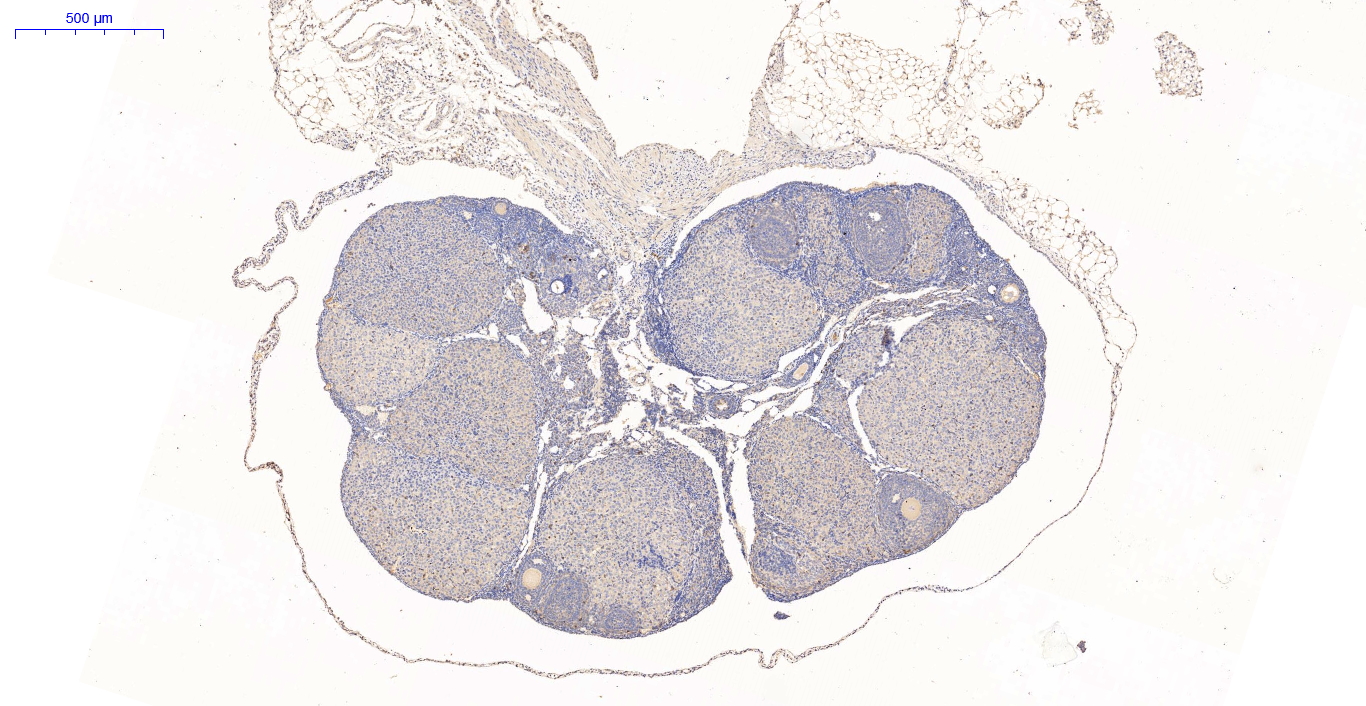

Supplement: Supplementary file 1 — Additional file 1. [file 13048_2021_854_MOESM1_ESM.zip › Supplementary Materials/Supplementary results of ovarian immunohistochemistry (whole ovarian section)/control group/Bcl-2 T_3.7x.jpg]

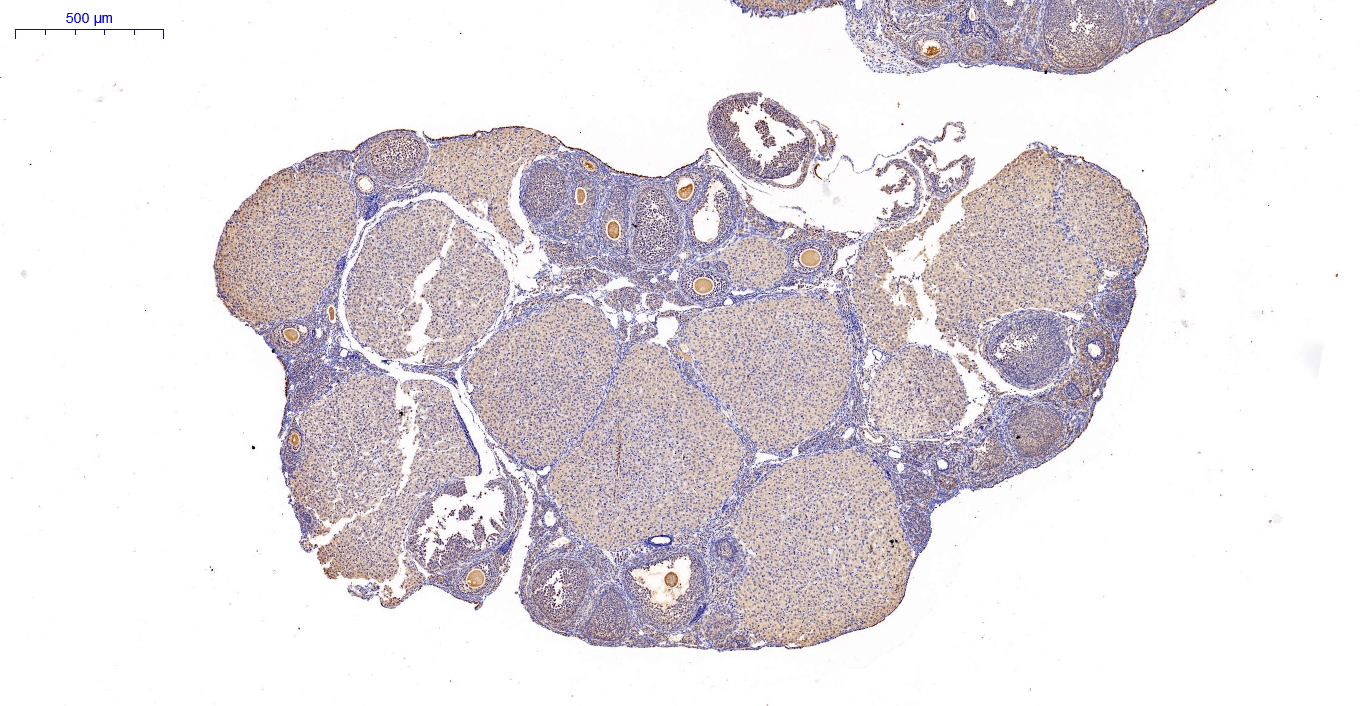

Supplement: Supplementary file 1 — Additional file 1. [file 13048_2021_854_MOESM1_ESM.zip › Supplementary Materials/Supplementary results of ovarian immunohistochemistry (whole ovarian section)/control group/FSHR T_3.7x.jpg]

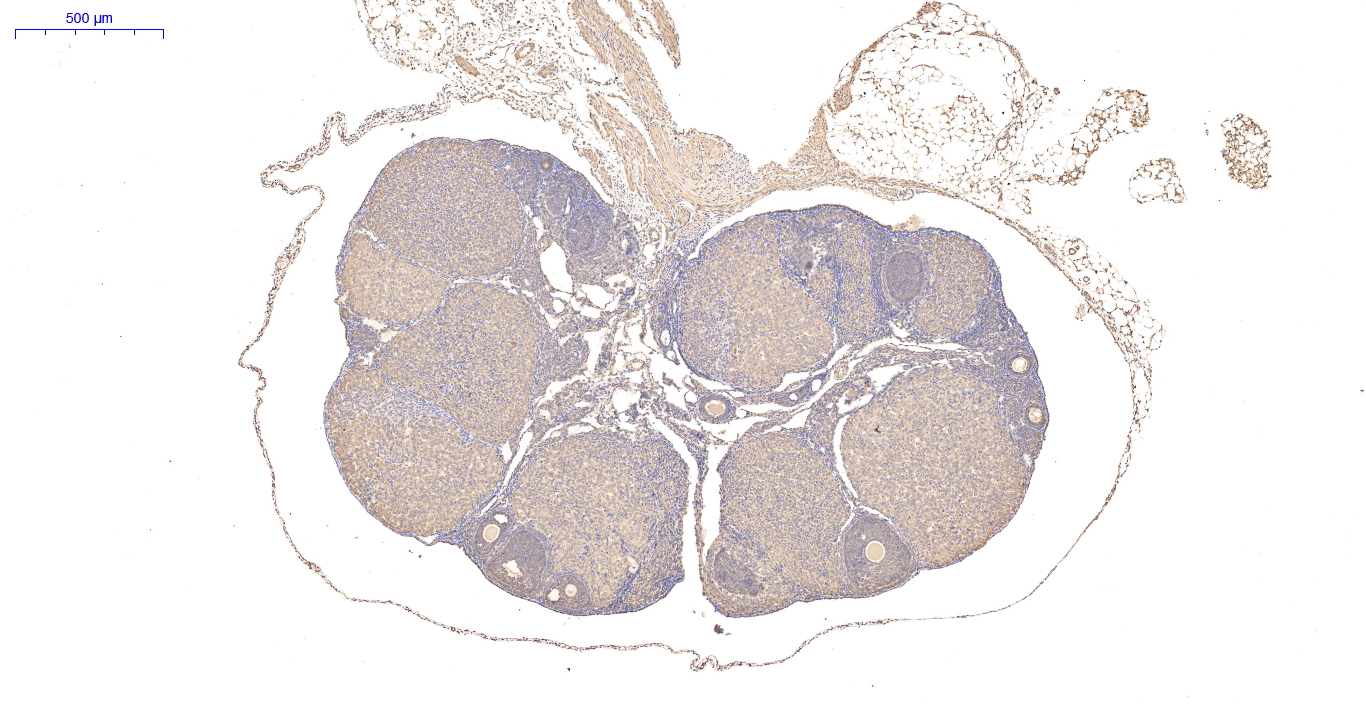

Supplement: Supplementary file 1 — Additional file 1. [file 13048_2021_854_MOESM1_ESM.zip › Supplementary Materials/Supplementary results of ovarian immunohistochemistry (whole ovarian section)/control group/LC36 T_3.7x.jpg]

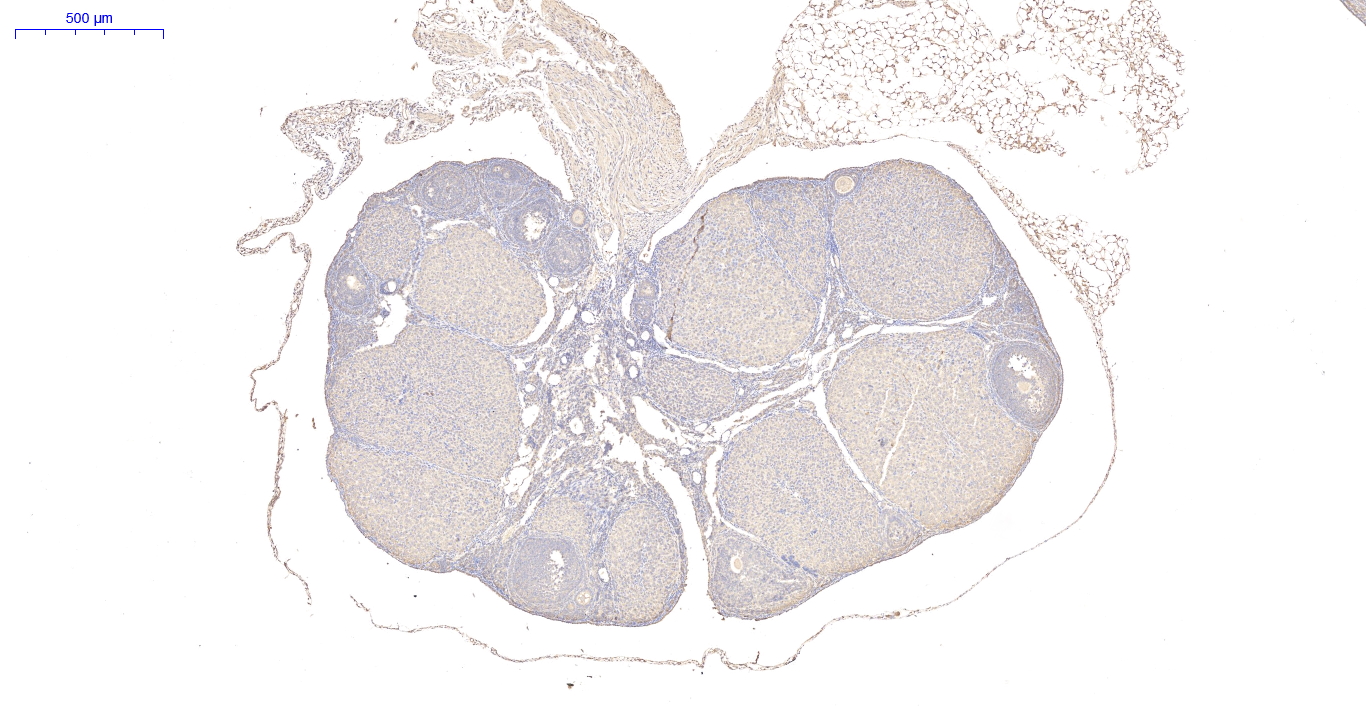

Supplement: Supplementary file 1 — Additional file 1. [file 13048_2021_854_MOESM1_ESM.zip › Supplementary Materials/Supplementary results of ovarian immunohistochemistry (whole ovarian section)/control group/P16 T_3.7x.jpg]

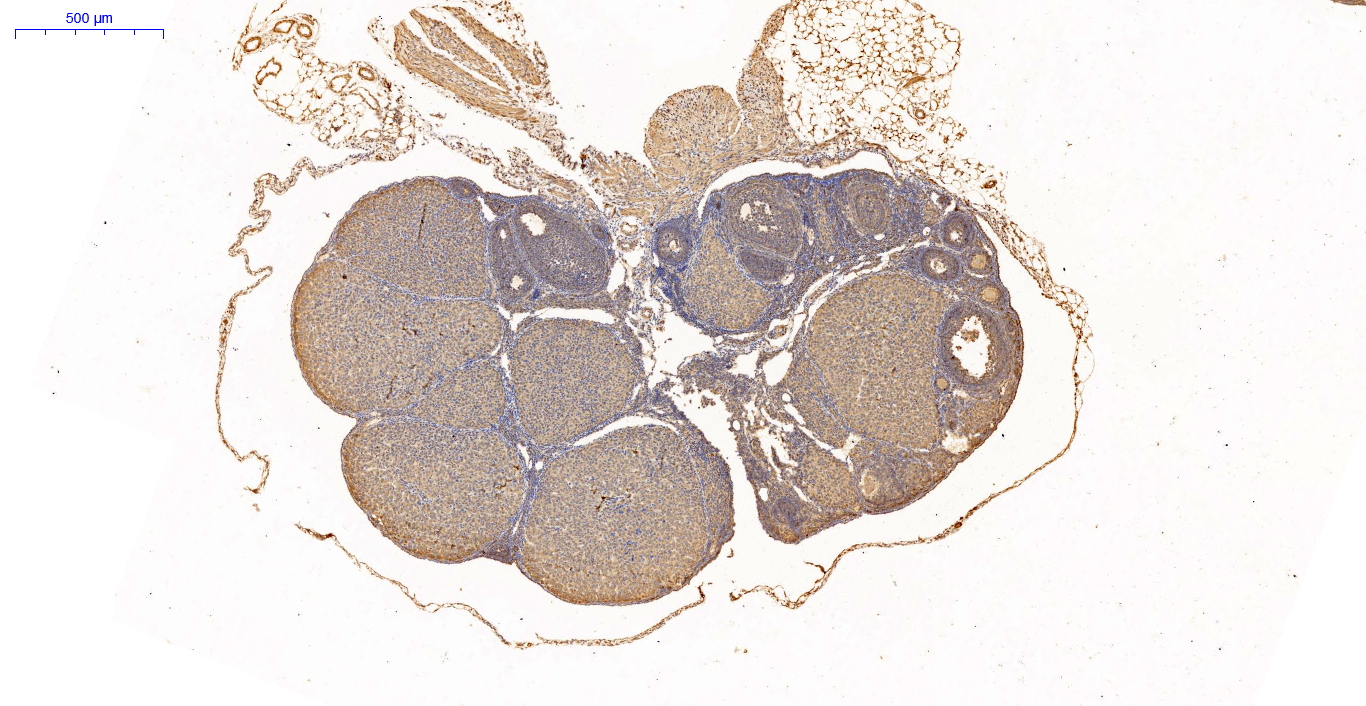

Supplement: Supplementary file 1 — Additional file 1. [file 13048_2021_854_MOESM1_ESM.zip › Supplementary Materials/Supplementary results of ovarian immunohistochemistry (whole ovarian section)/control group/P53 T_3.7x.jpg]

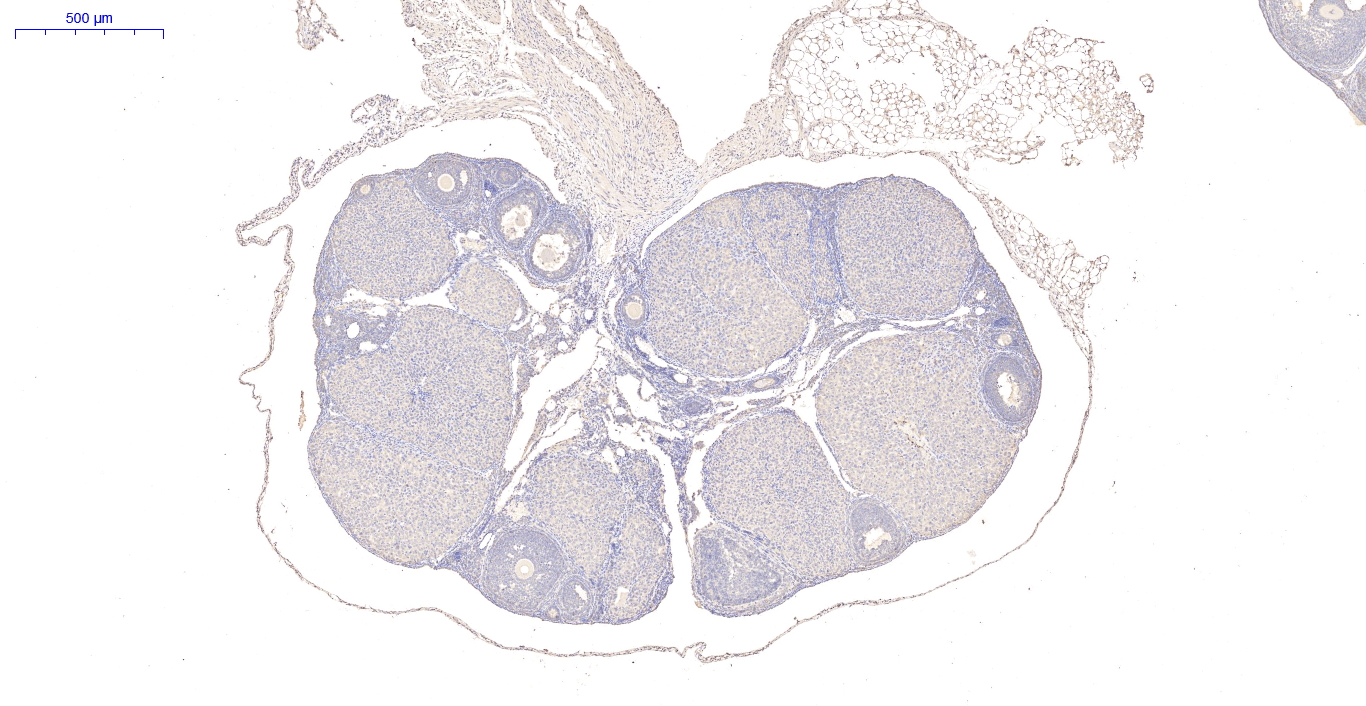

Supplement: Supplementary file 1 — Additional file 1. [file 13048_2021_854_MOESM1_ESM.zip › Supplementary Materials/Supplementary results of ovarian immunohistochemistry (whole ovarian section)/control group/P62_3.7x.jpg]

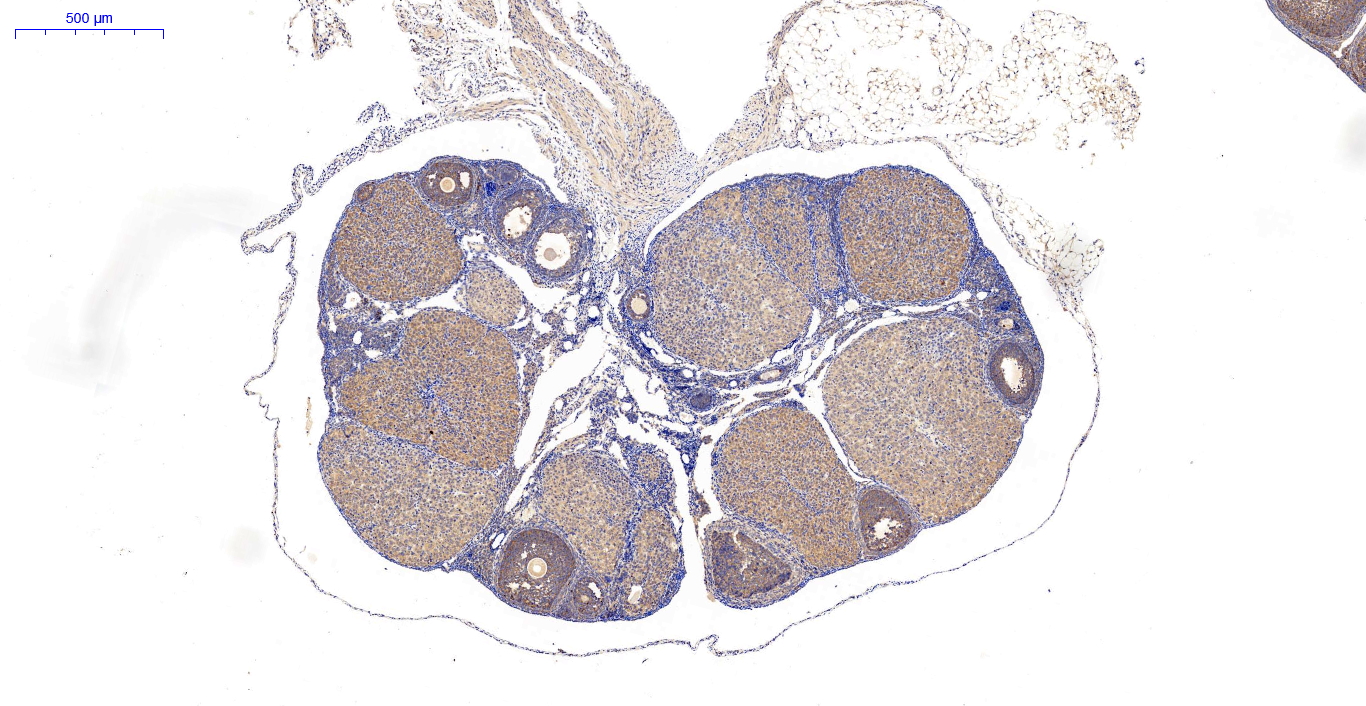

Supplement: Supplementary file 1 — Additional file 1. [file 13048_2021_854_MOESM1_ESM.zip › Supplementary Materials/Supplementary results of ovarian immunohistochemistry (whole ovarian section)/control group/SOD1 T_3.7x.jpg]

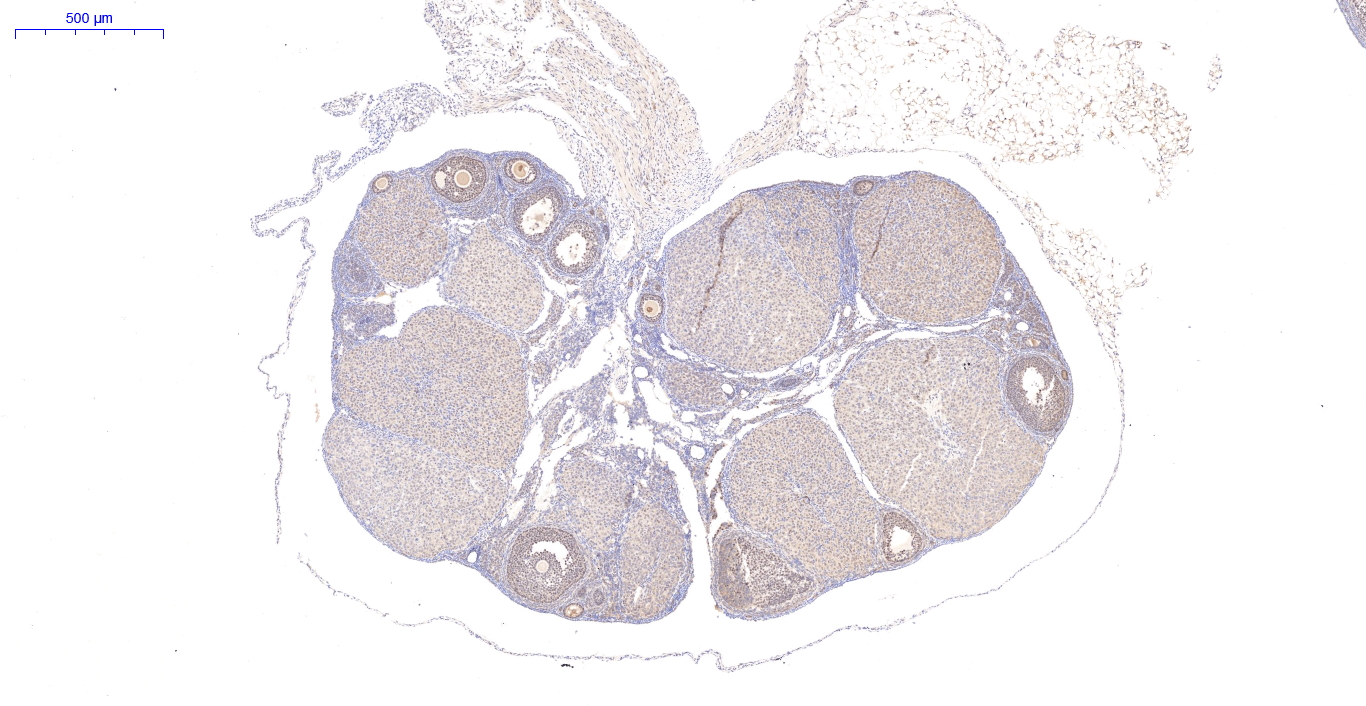

Supplement: Supplementary file 1 — Additional file 1. [file 13048_2021_854_MOESM1_ESM.zip › Supplementary Materials/Supplementary results of ovarian immunohistochemistry (whole ovarian section)/control group/Sirt-1 T_3.7x.jpg]

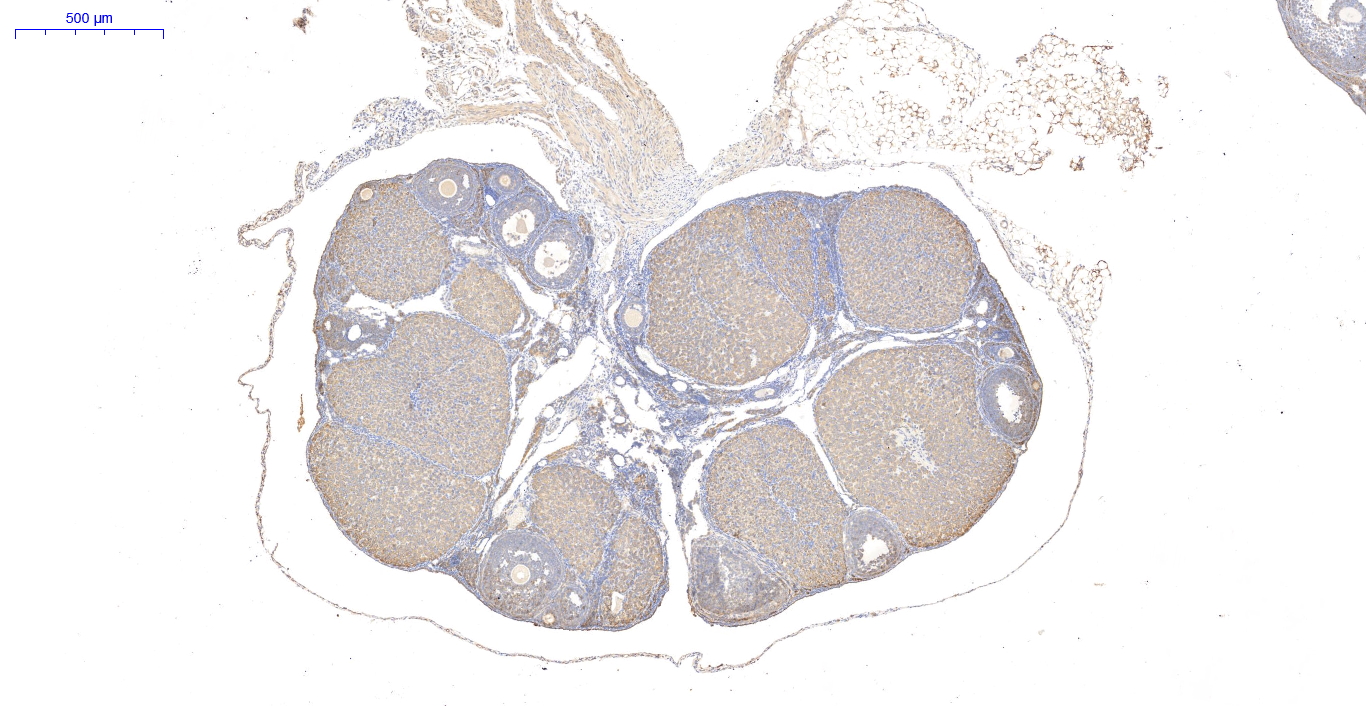

Supplement: Supplementary file 1 — Additional file 1. [file 13048_2021_854_MOESM1_ESM.zip › Supplementary Materials/Supplementary results of ovarian immunohistochemistry (whole ovarian section)/control group/becline-1 T_3.7x.jpg]

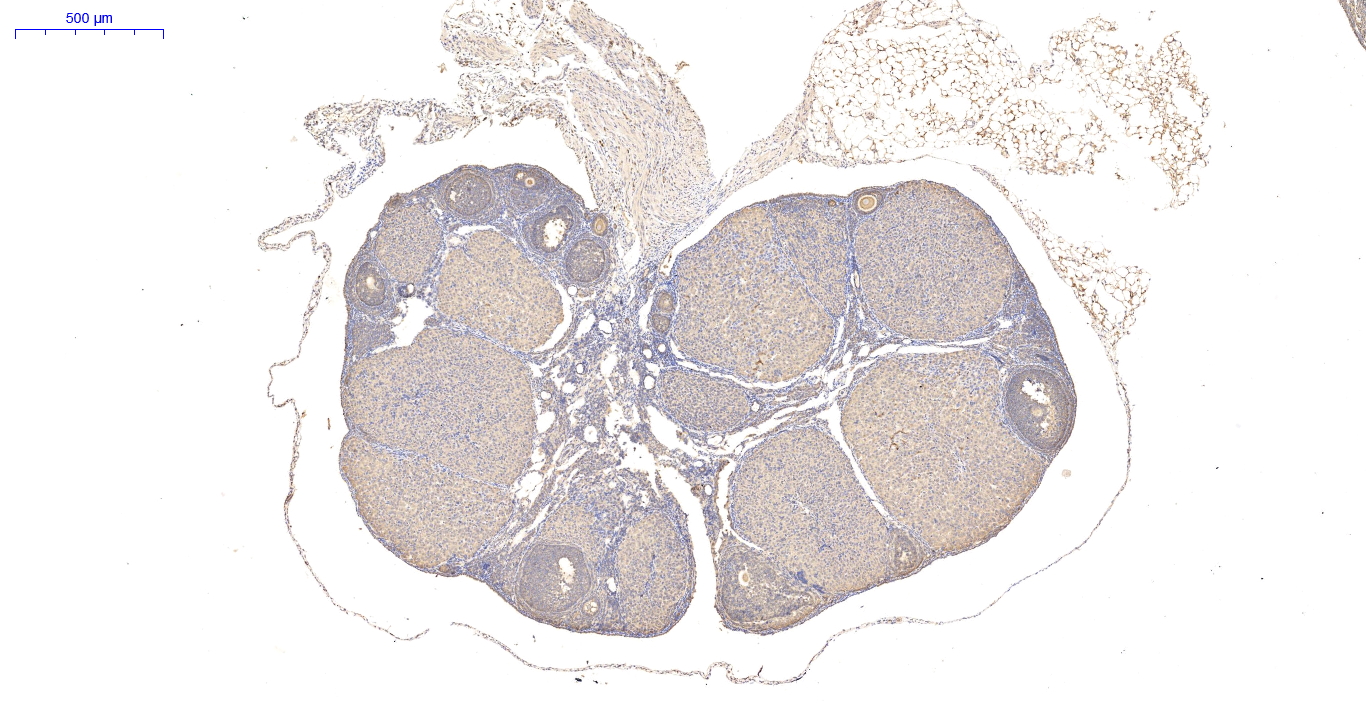

Supplement: Supplementary file 1 — Additional file 1. [file 13048_2021_854_MOESM1_ESM.zip › Supplementary Materials/Supplementary results of ovarian immunohistochemistry (whole ovarian section)/control group/caspase-3 T_3.7x.jpg]

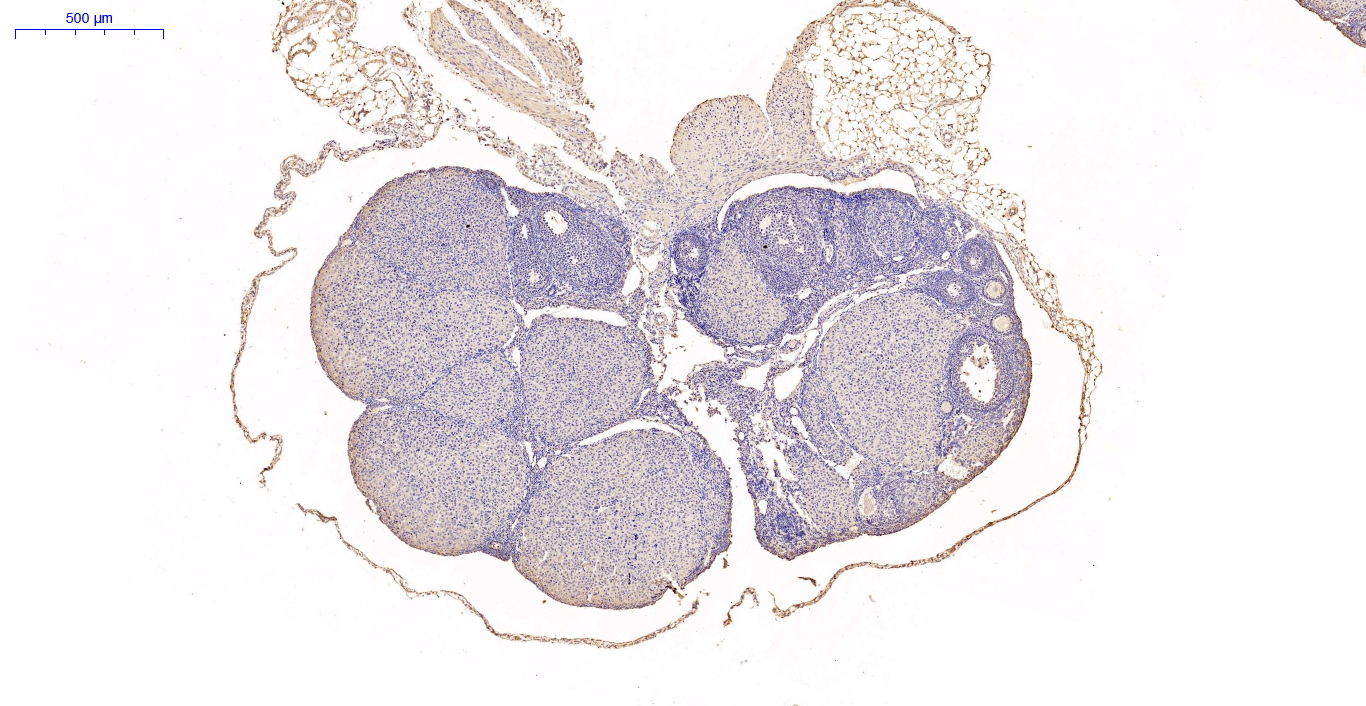

Supplement: Supplementary file 1 — Additional file 1. [file 13048_2021_854_MOESM1_ESM.zip › Supplementary Materials/Supplementary results of ovarian immunohistochemistry (whole ovarian section)/control group/sirt3 T_3.7x.jpg]

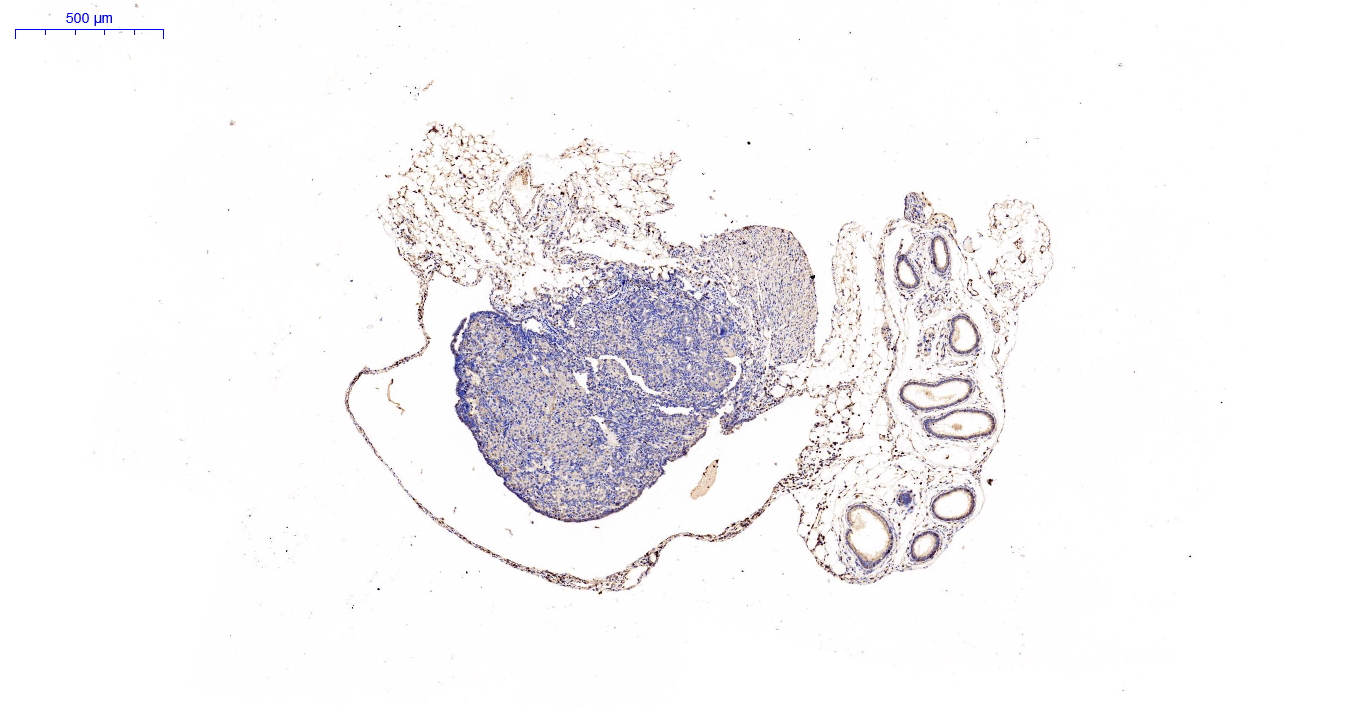

Supplement: Supplementary file 1 — Additional file 1. [file 13048_2021_854_MOESM1_ESM.zip › Supplementary Materials/Supplementary results of ovarian immunohistochemistry (whole ovarian section)/model group/Bax T_3.7x.jpg]

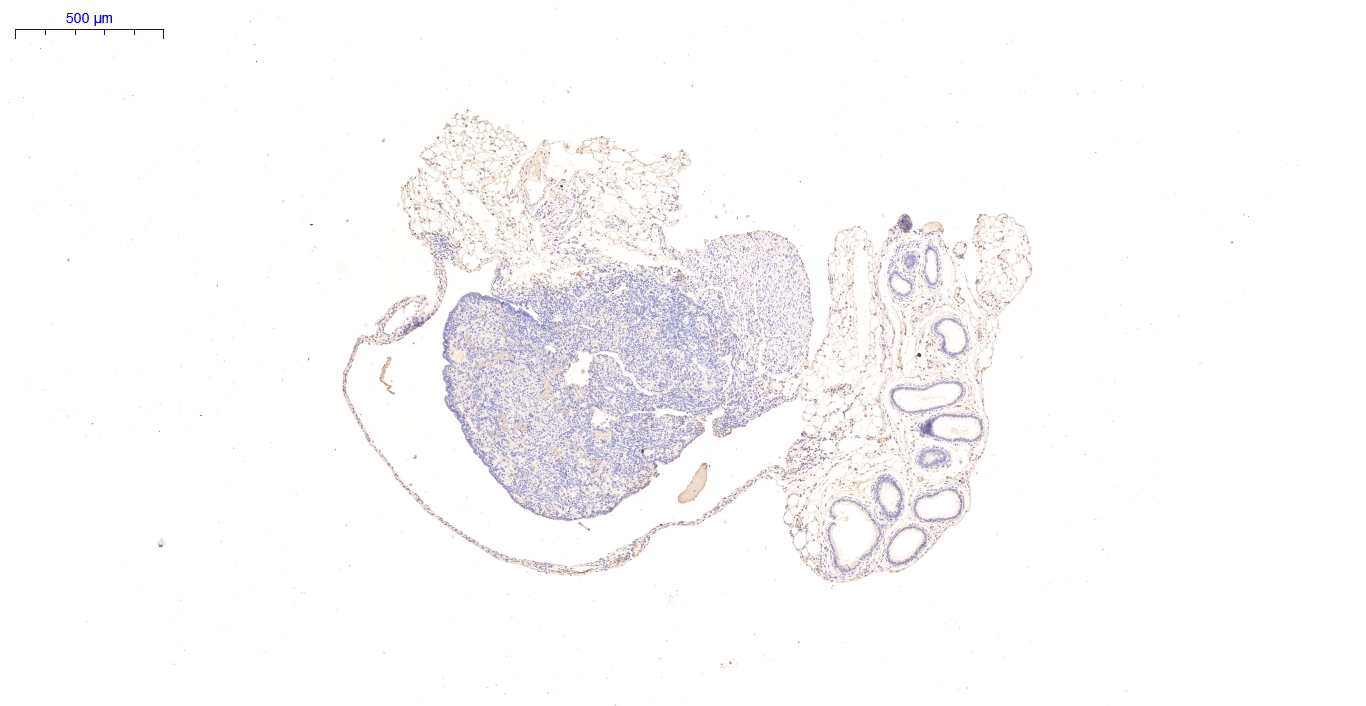

Supplement: Supplementary file 1 — Additional file 1. [file 13048_2021_854_MOESM1_ESM.zip › Supplementary Materials/Supplementary results of ovarian immunohistochemistry (whole ovarian section)/model group/Bd-2 T_3.7x.jpg]

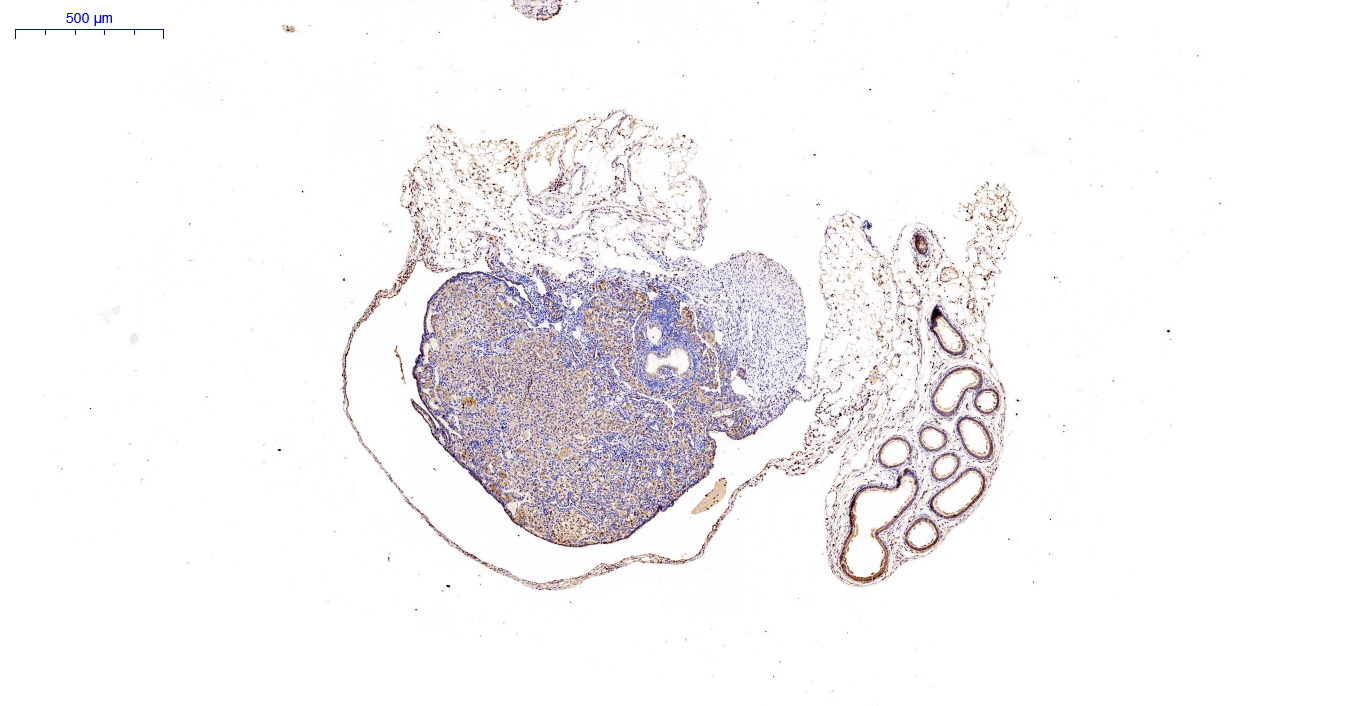

Supplement: Supplementary file 1 — Additional file 1. [file 13048_2021_854_MOESM1_ESM.zip › Supplementary Materials/Supplementary results of ovarian immunohistochemistry (whole ovarian section)/model group/Beclin-1 T_3.7x.jpg]

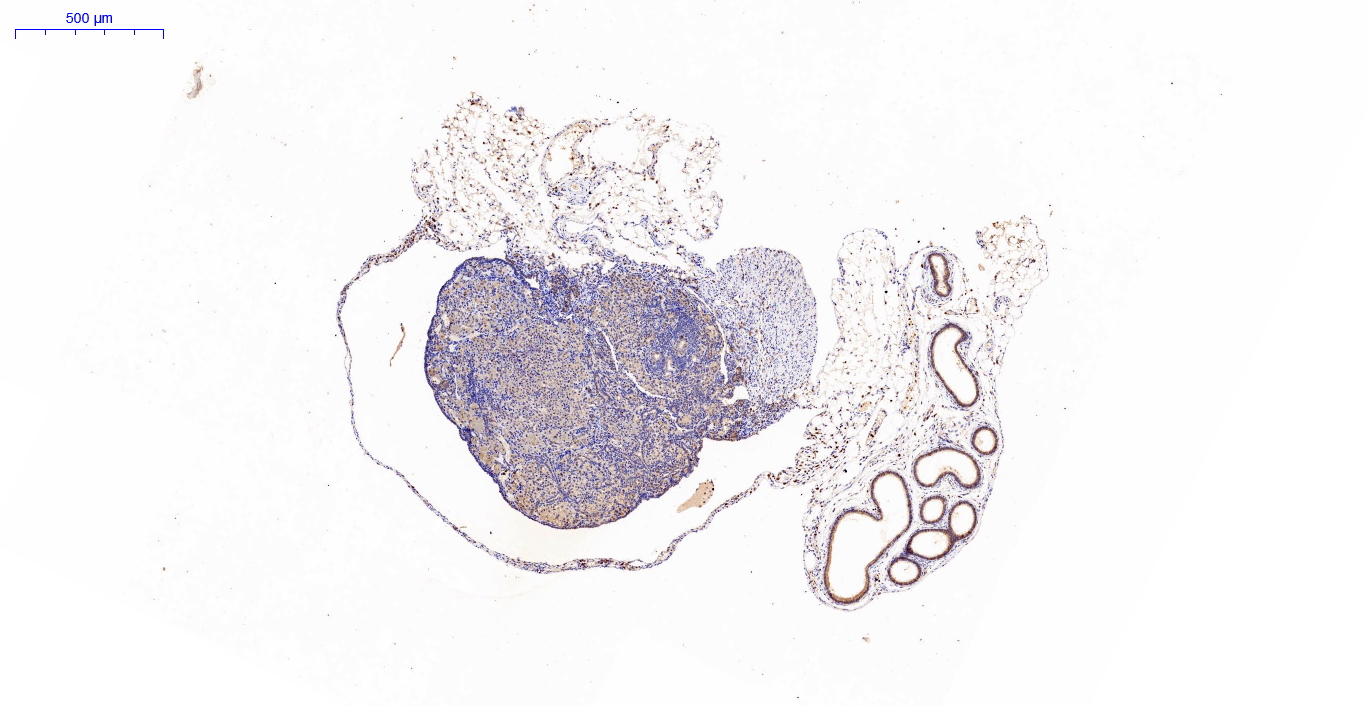

Supplement: Supplementary file 1 — Additional file 1. [file 13048_2021_854_MOESM1_ESM.zip › Supplementary Materials/Supplementary results of ovarian immunohistochemistry (whole ovarian section)/model group/Caspase3 T_3.7x.jpg]

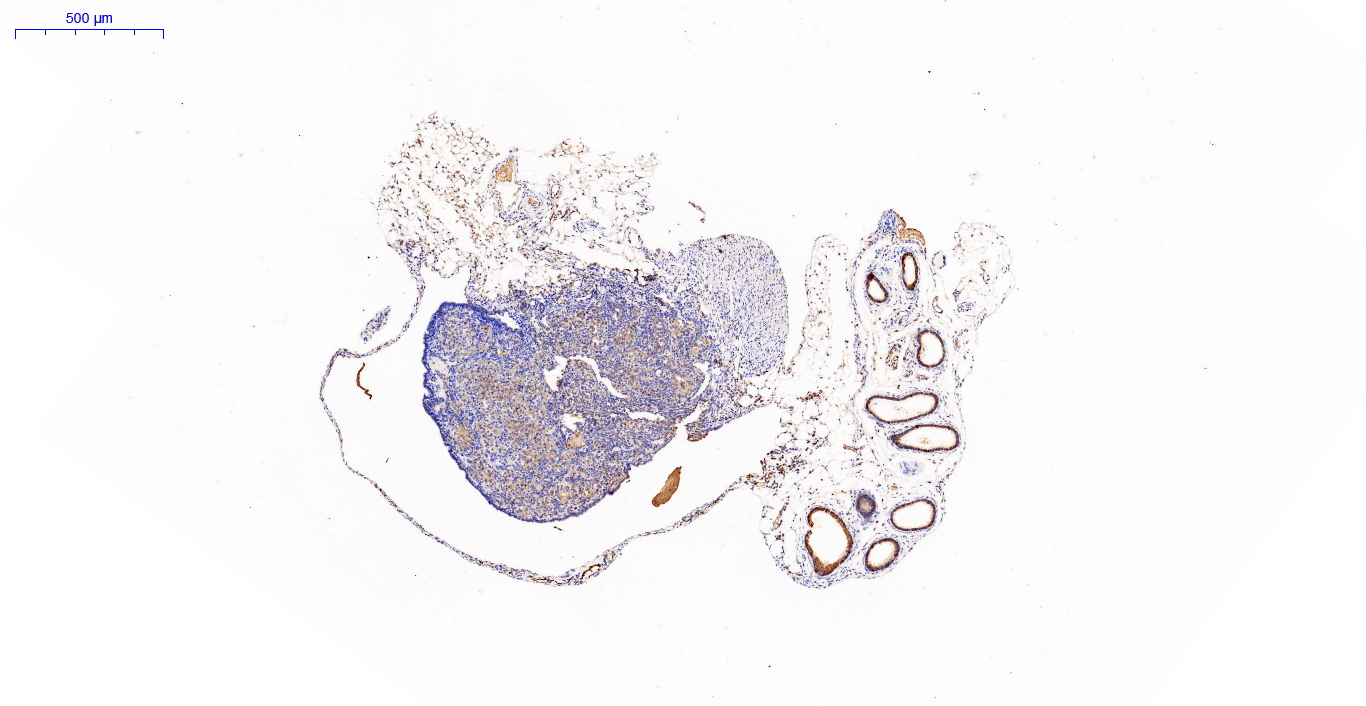

Supplement: Supplementary file 1 — Additional file 1. [file 13048_2021_854_MOESM1_ESM.zip › Supplementary Materials/Supplementary results of ovarian immunohistochemistry (whole ovarian section)/model group/FSHR T_3.7x.jpg]

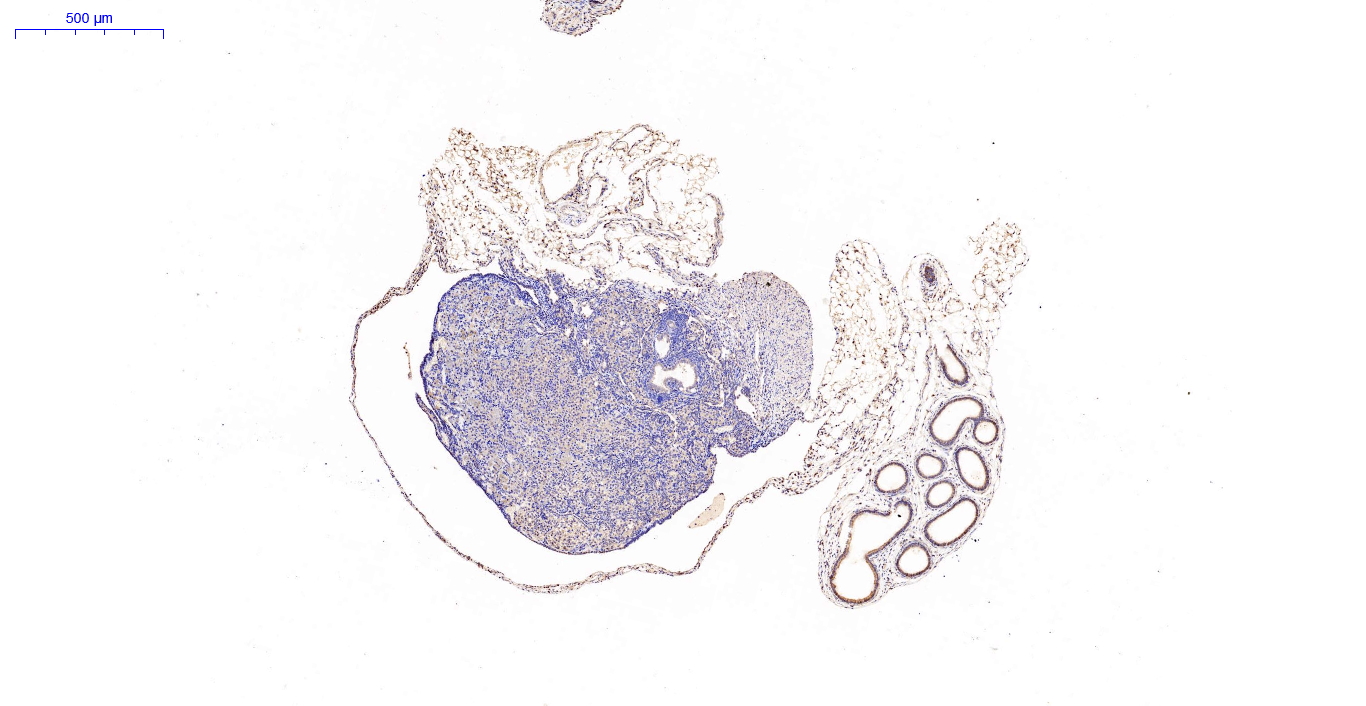

Supplement: Supplementary file 1 — Additional file 1. [file 13048_2021_854_MOESM1_ESM.zip › Supplementary Materials/Supplementary results of ovarian immunohistochemistry (whole ovarian section)/model group/LC36 T_3.7x.jpg]

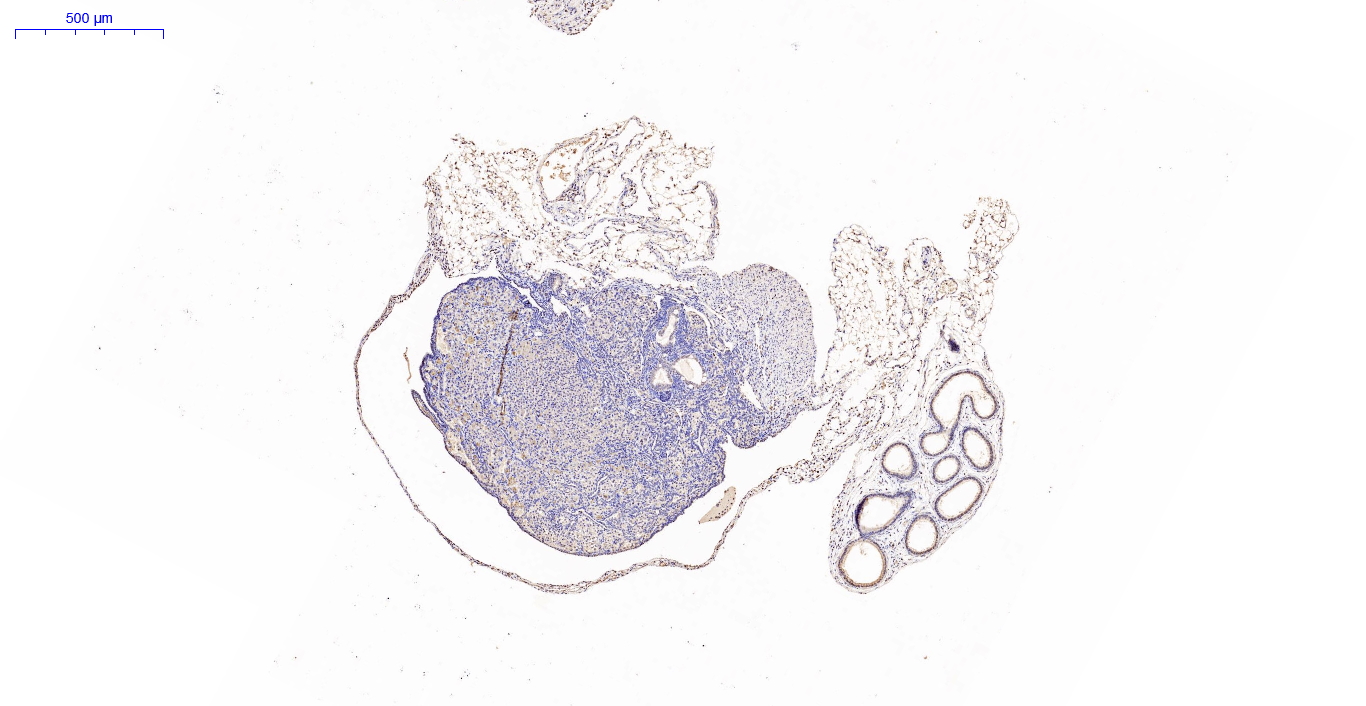

Supplement: Supplementary file 1 — Additional file 1. [file 13048_2021_854_MOESM1_ESM.zip › Supplementary Materials/Supplementary results of ovarian immunohistochemistry (whole ovarian section)/model group/P16 T_3.7x.jpg]

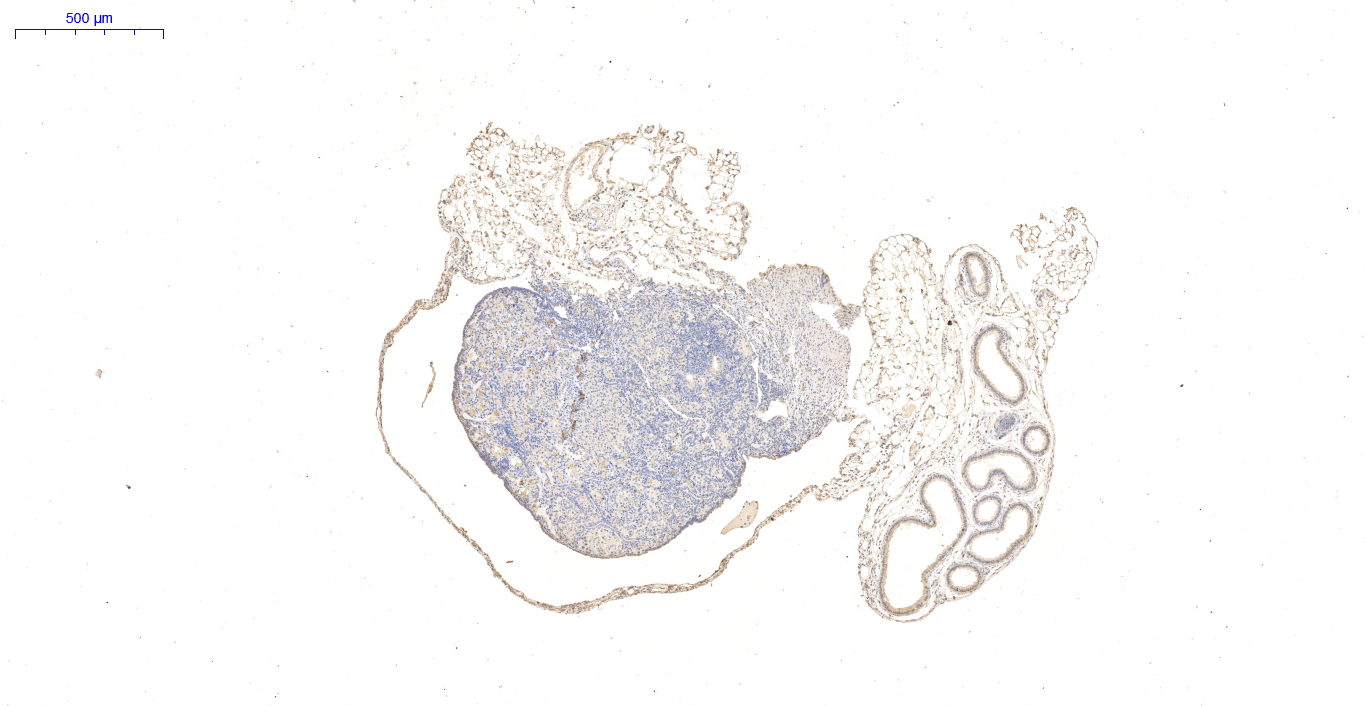

Supplement: Supplementary file 1 — Additional file 1. [file 13048_2021_854_MOESM1_ESM.zip › Supplementary Materials/Supplementary results of ovarian immunohistochemistry (whole ovarian section)/model group/P53_3.7x.jpg]

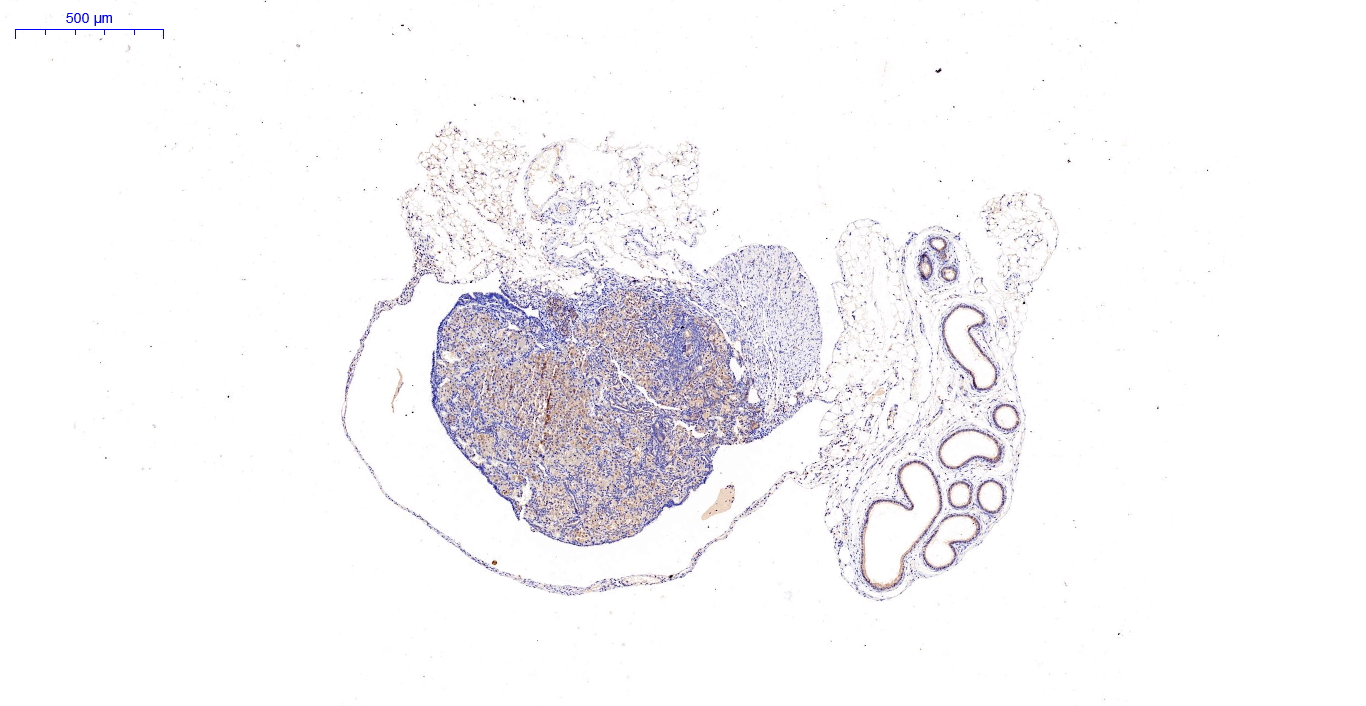

Supplement: Supplementary file 1 — Additional file 1. [file 13048_2021_854_MOESM1_ESM.zip › Supplementary Materials/Supplementary results of ovarian immunohistochemistry (whole ovarian section)/model group/P62 T_3.7x.jpg]

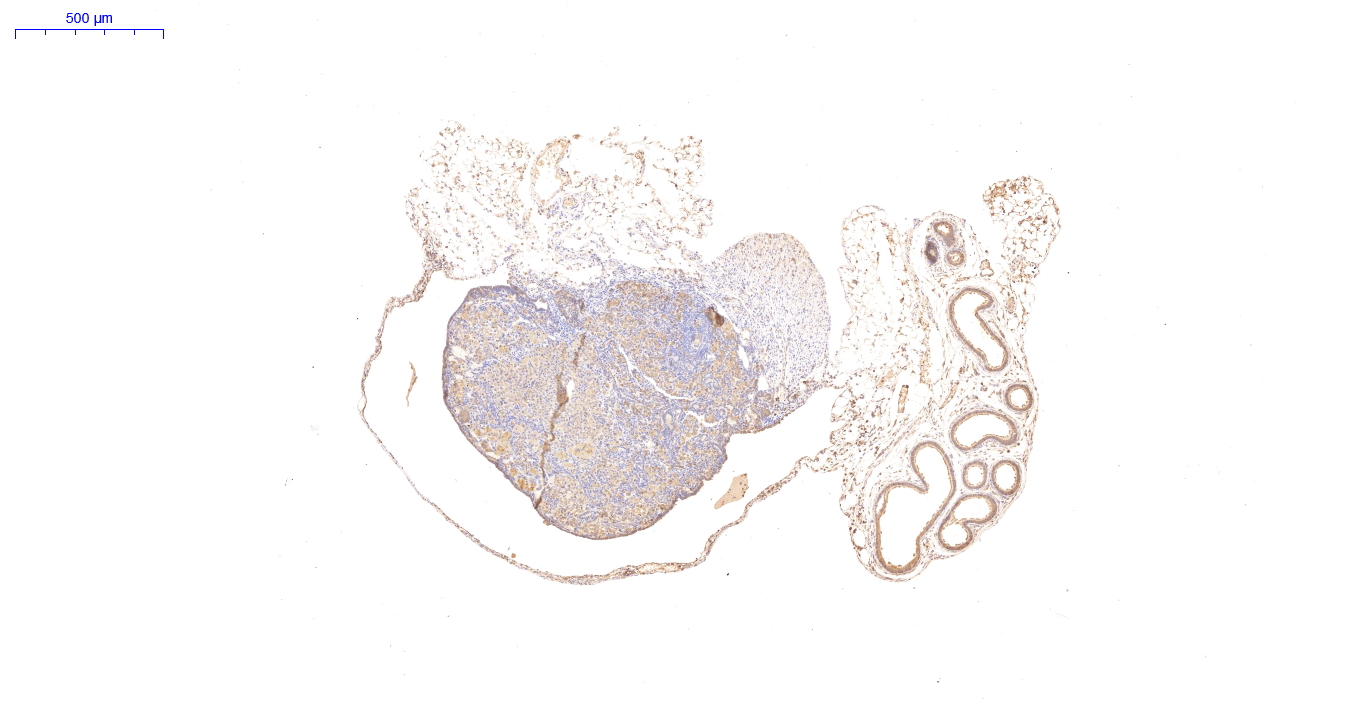

Supplement: Supplementary file 1 — Additional file 1. [file 13048_2021_854_MOESM1_ESM.zip › Supplementary Materials/Supplementary results of ovarian immunohistochemistry (whole ovarian section)/model group/SOD 1 T_3.7x.jpg]

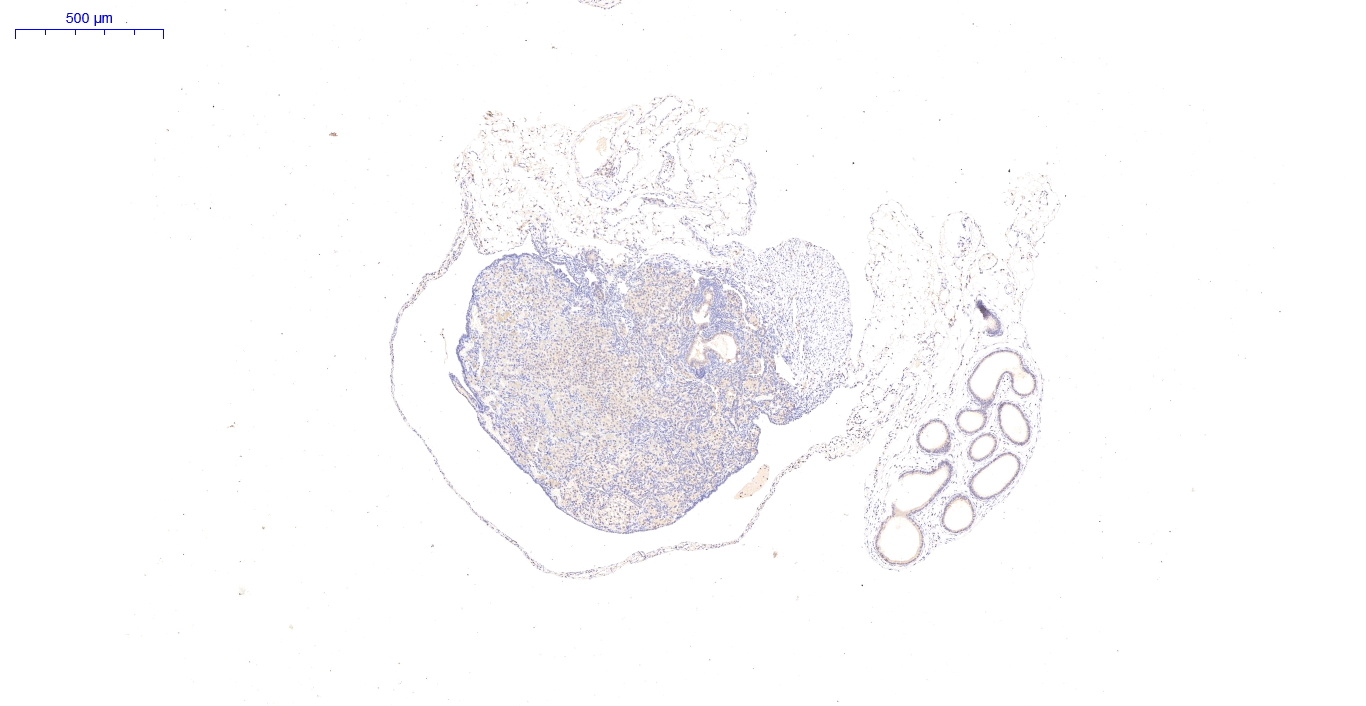

Supplement: Supplementary file 1 — Additional file 1. [file 13048_2021_854_MOESM1_ESM.zip › Supplementary Materials/Supplementary results of ovarian immunohistochemistry (whole ovarian section)/model group/sirt1 T_3.7x.jpg]

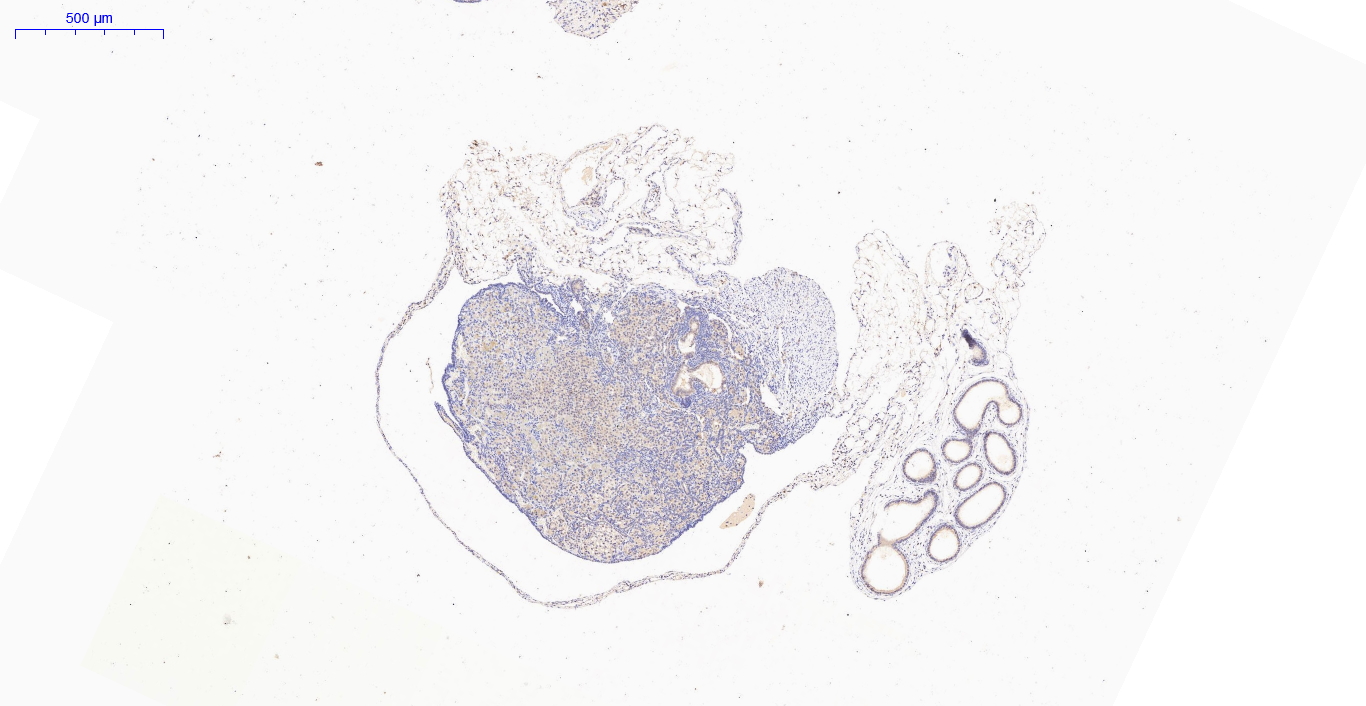

Supplement: Supplementary file 1 — Additional file 1. [file 13048_2021_854_MOESM1_ESM.zip › Supplementary Materials/Supplementary results of ovarian immunohistochemistry (whole ovarian section)/model group/sirt3 T_3.7x.jpg]

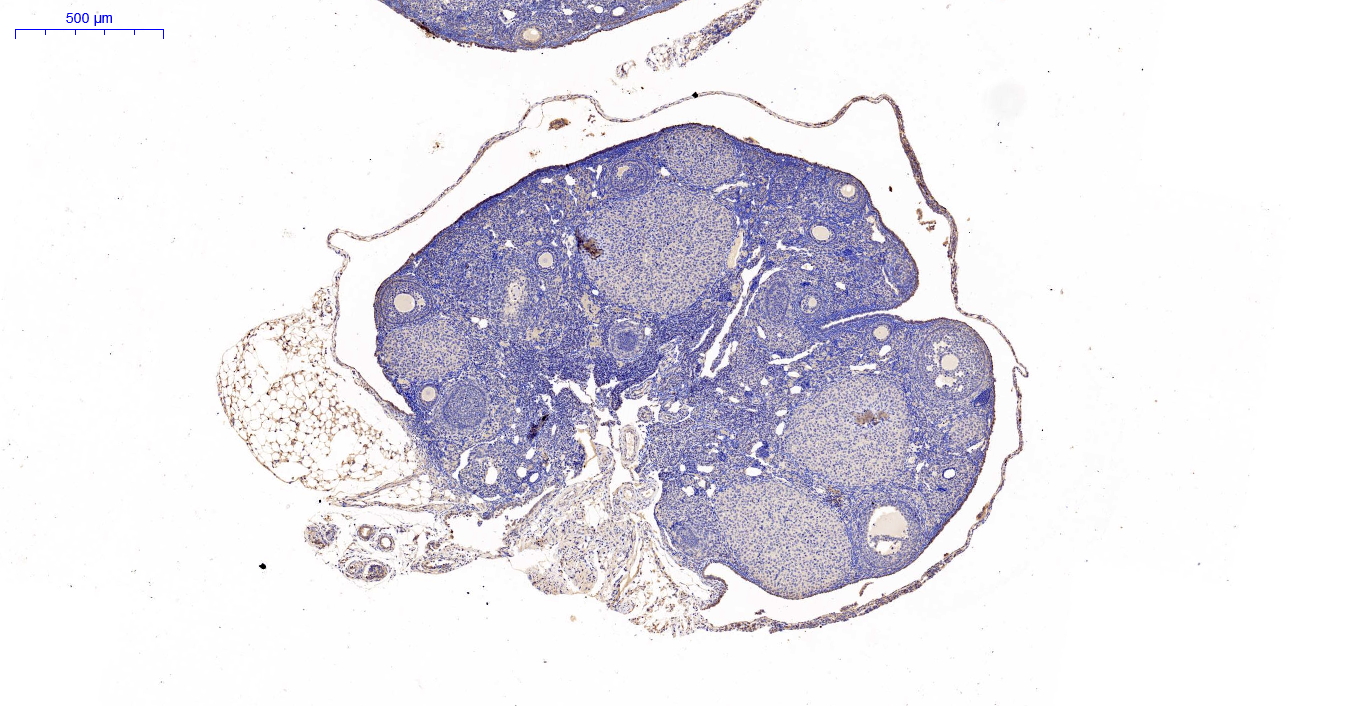

Supplement: Supplementary file 1 — Additional file 1. [file 13048_2021_854_MOESM1_ESM.zip › Supplementary Materials/Supplementary results of ovarian immunohistochemistry (whole ovarian section)/treatment group/Bax_3.7x.jpg]

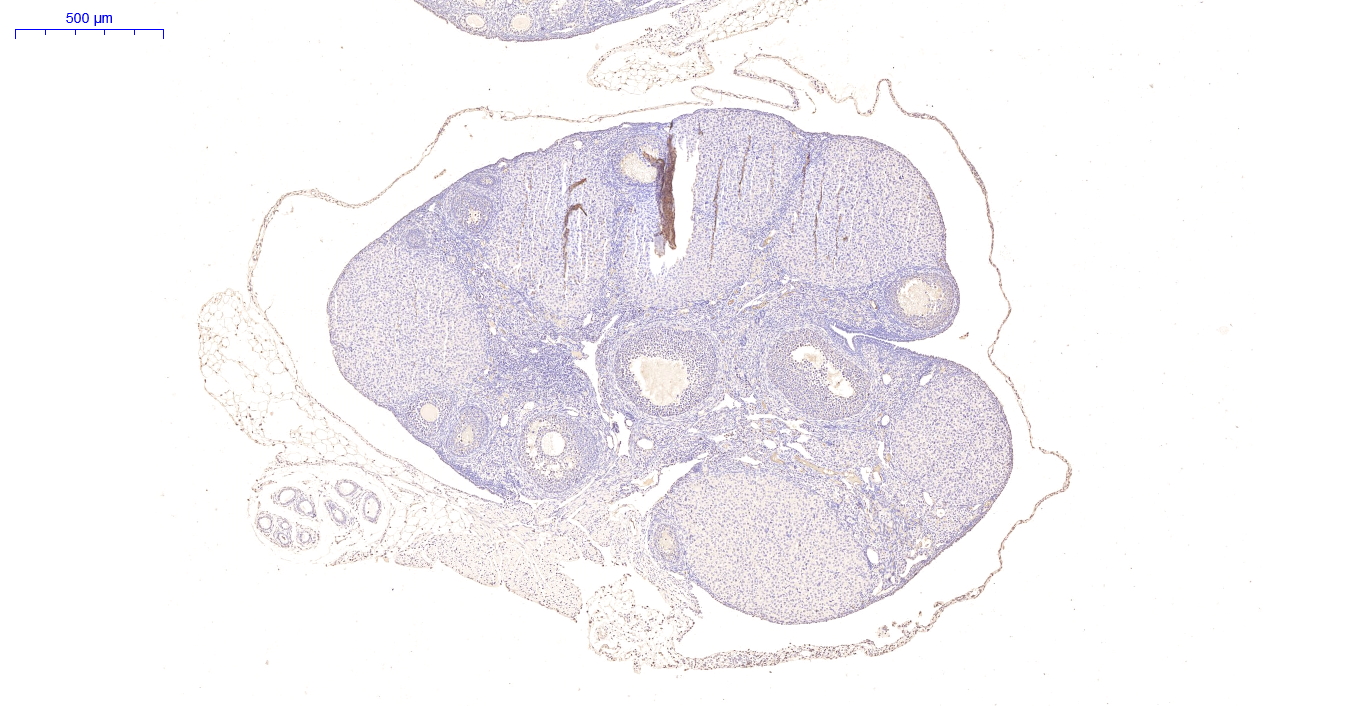

Supplement: Supplementary file 1 — Additional file 1. [file 13048_2021_854_MOESM1_ESM.zip › Supplementary Materials/Supplementary results of ovarian immunohistochemistry (whole ovarian section)/treatment group/Bd-2 T_3.7x.jpg]

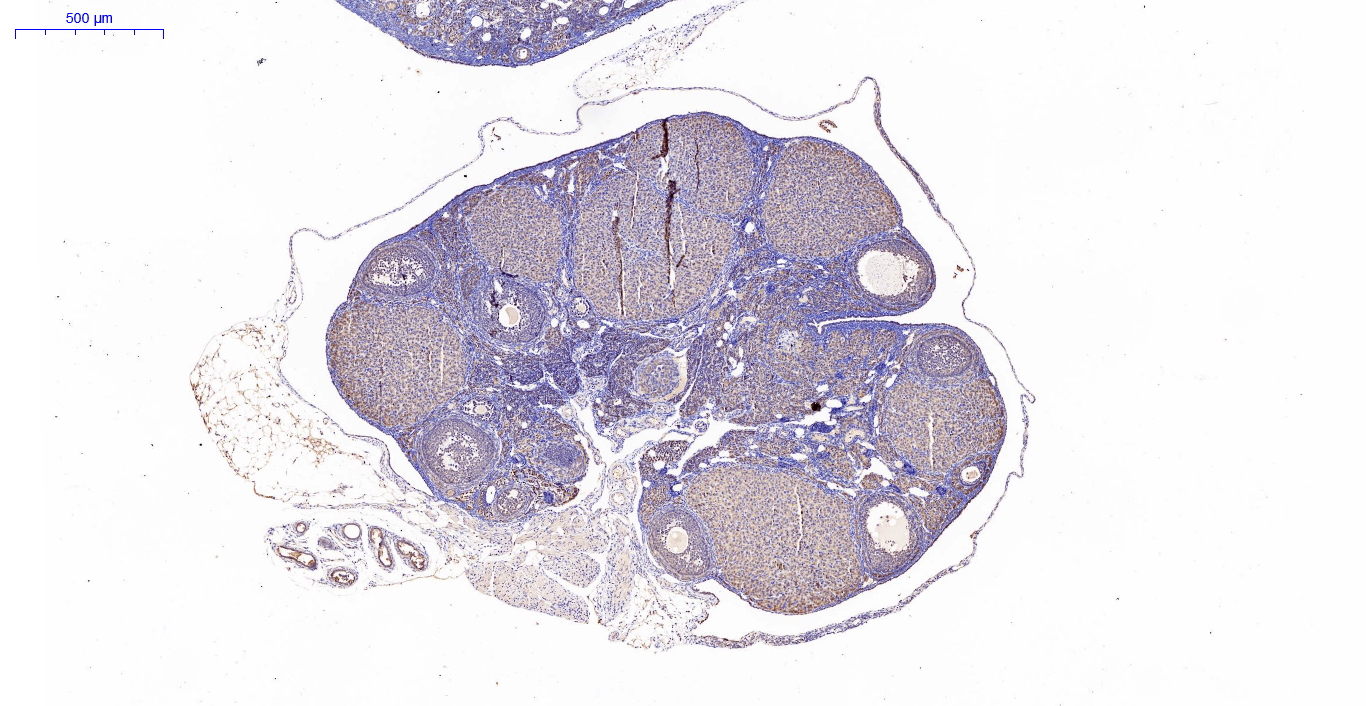

Supplement: Supplementary file 1 — Additional file 1. [file 13048_2021_854_MOESM1_ESM.zip › Supplementary Materials/Supplementary results of ovarian immunohistochemistry (whole ovarian section)/treatment group/Beclin-1 T_3.7x.jpg]

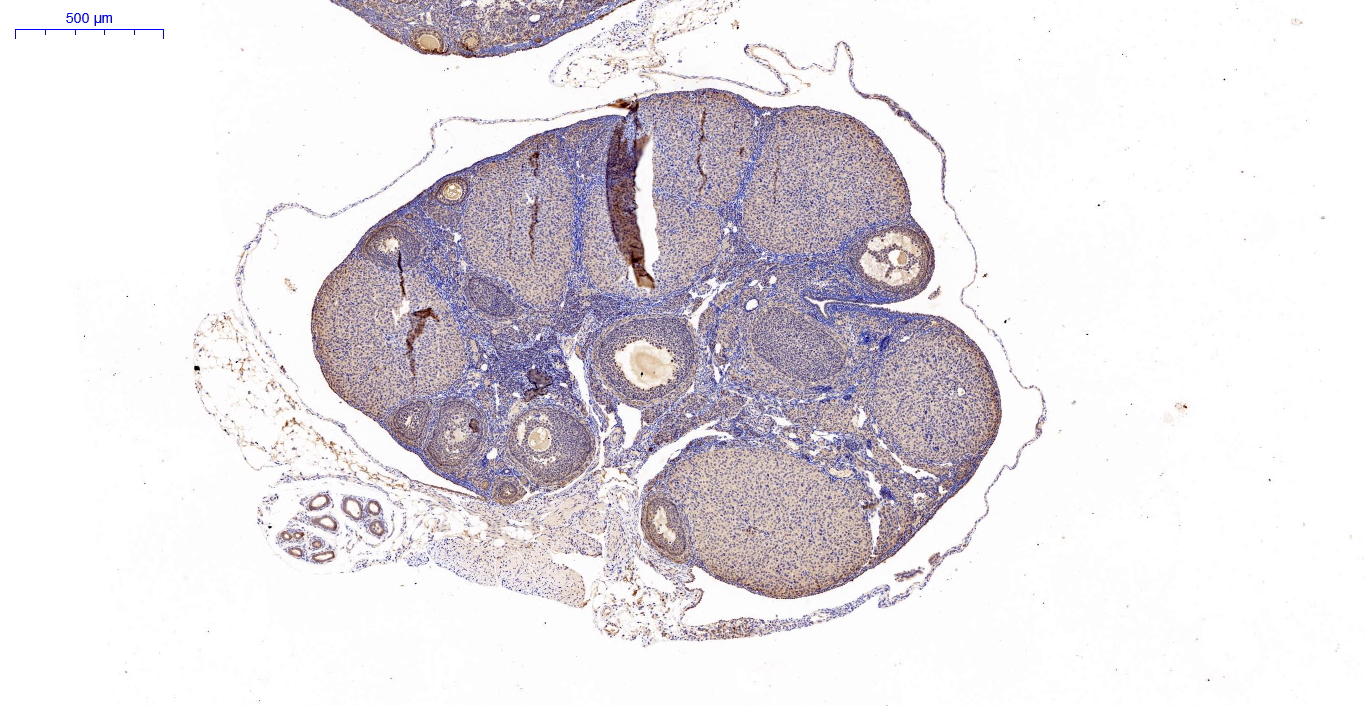

Supplement: Supplementary file 1 — Additional file 1. [file 13048_2021_854_MOESM1_ESM.zip › Supplementary Materials/Supplementary results of ovarian immunohistochemistry (whole ovarian section)/treatment group/Caspase3 T_3.7x.jpg]

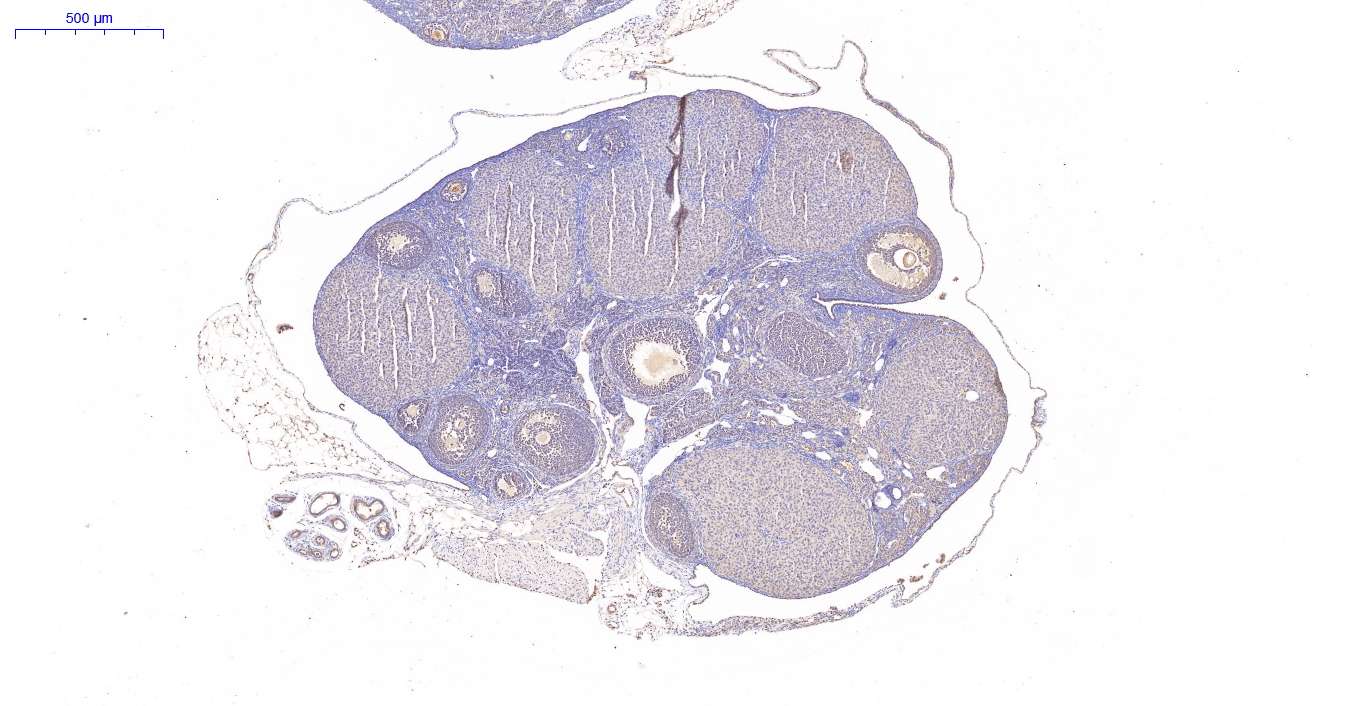

Supplement: Supplementary file 1 — Additional file 1. [file 13048_2021_854_MOESM1_ESM.zip › Supplementary Materials/Supplementary results of ovarian immunohistochemistry (whole ovarian section)/treatment group/FSHR T_3.7x.jpg]

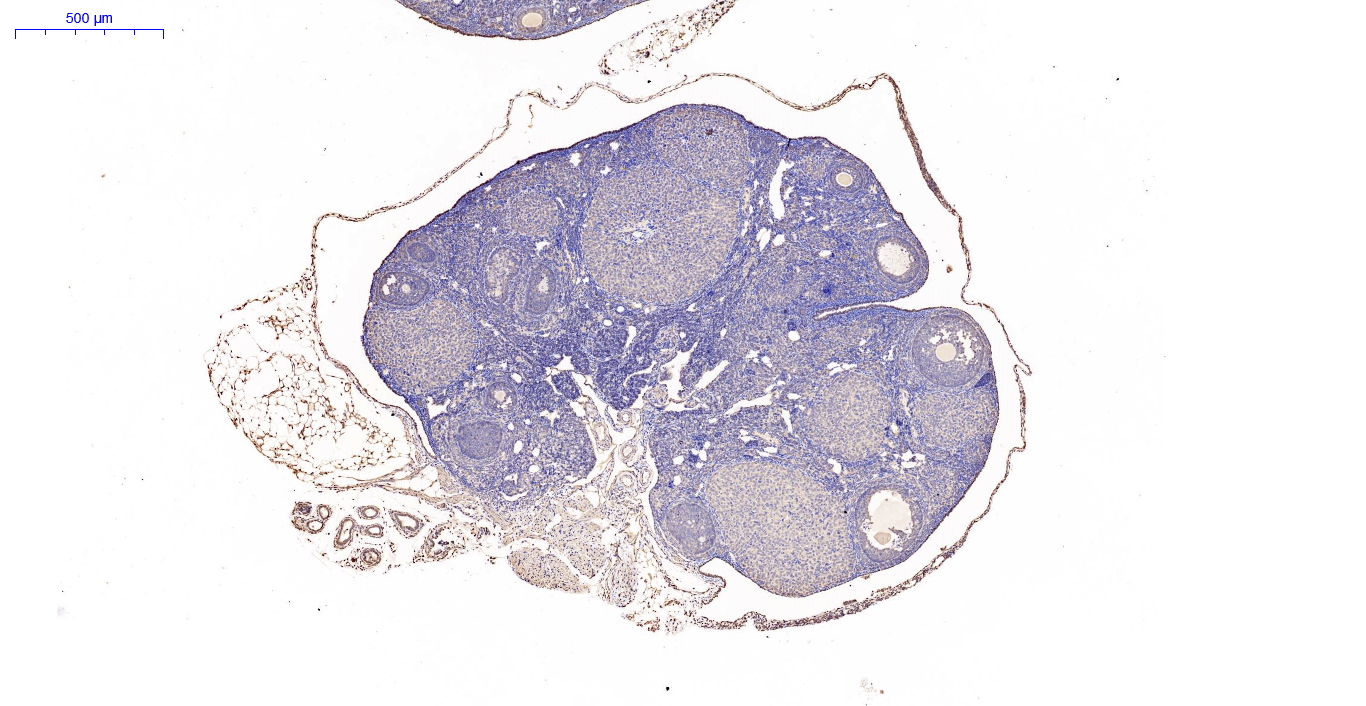

Supplement: Supplementary file 1 — Additional file 1. [file 13048_2021_854_MOESM1_ESM.zip › Supplementary Materials/Supplementary results of ovarian immunohistochemistry (whole ovarian section)/treatment group/LC36 T_3.7x.jpg]

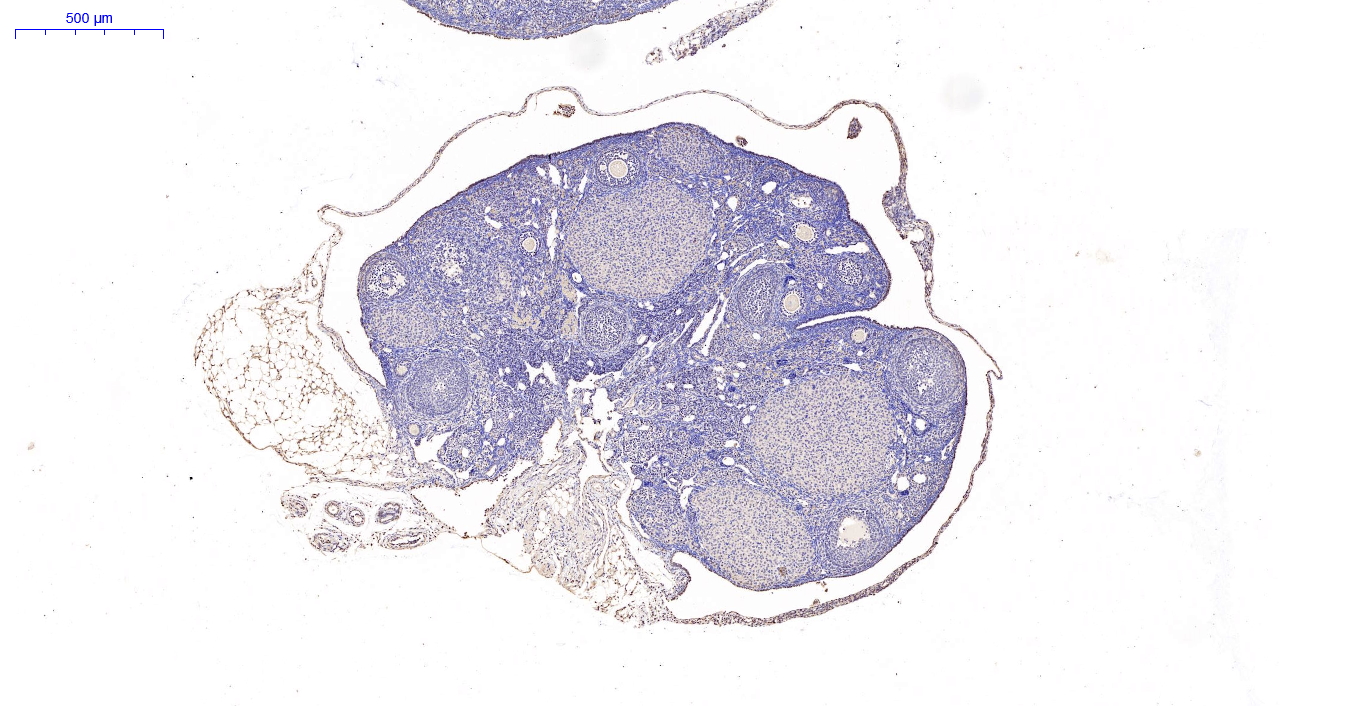

Supplement: Supplementary file 1 — Additional file 1. [file 13048_2021_854_MOESM1_ESM.zip › Supplementary Materials/Supplementary results of ovarian immunohistochemistry (whole ovarian section)/treatment group/P16 T_3.7x.jpg]

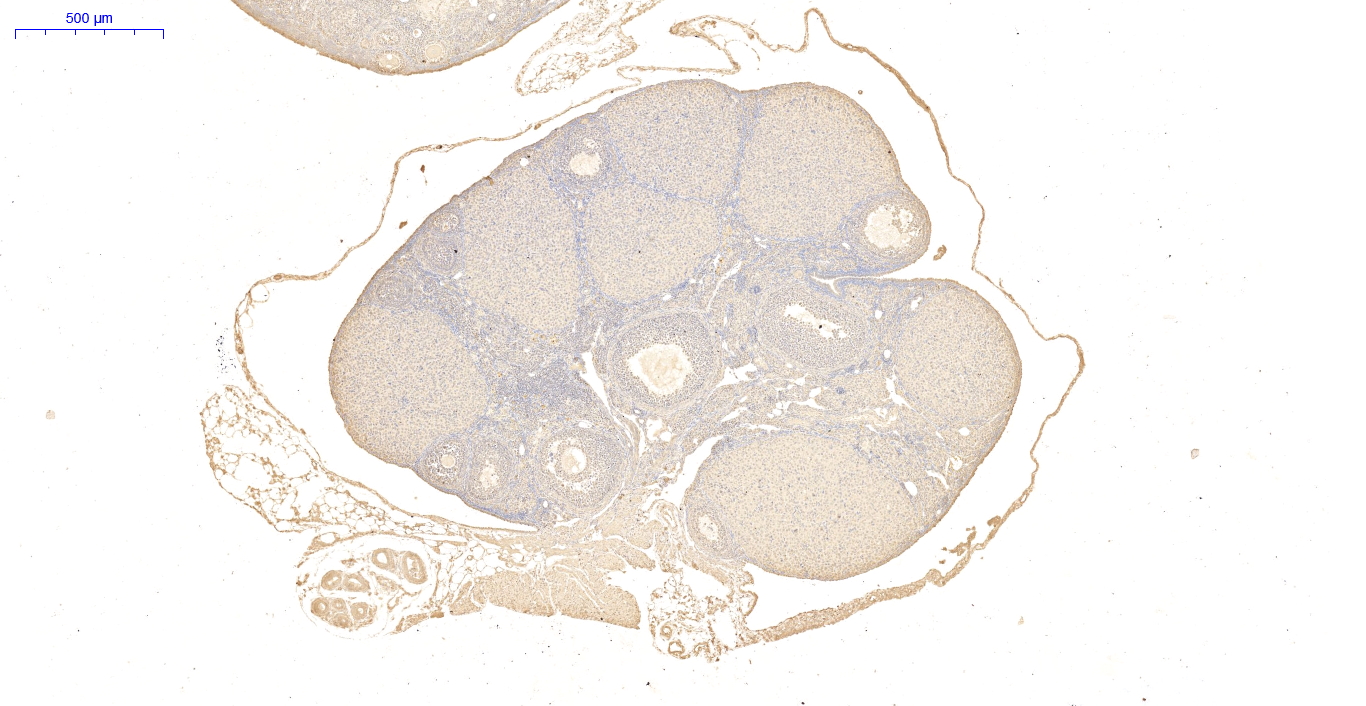

Supplement: Supplementary file 1 — Additional file 1. [file 13048_2021_854_MOESM1_ESM.zip › Supplementary Materials/Supplementary results of ovarian immunohistochemistry (whole ovarian section)/treatment group/P53_3.7x.jpg]

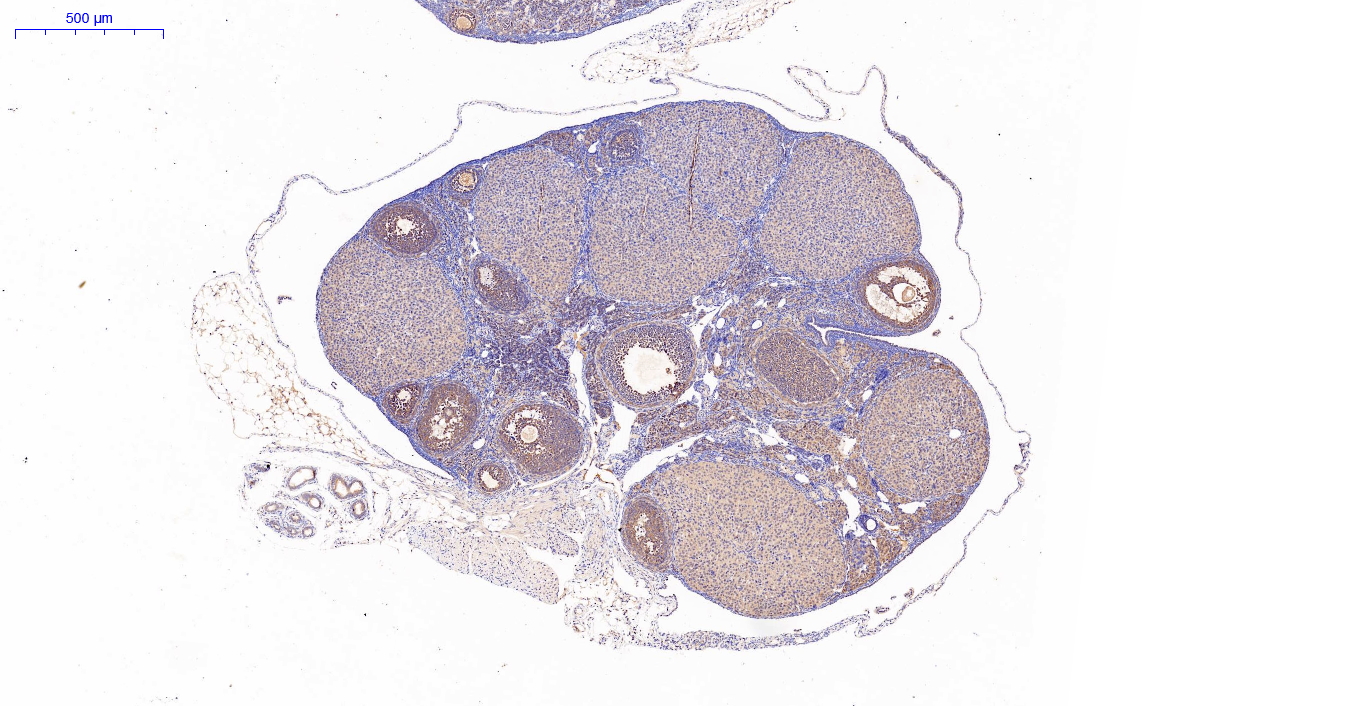

Supplement: Supplementary file 1 — Additional file 1. [file 13048_2021_854_MOESM1_ESM.zip › Supplementary Materials/Supplementary results of ovarian immunohistochemistry (whole ovarian section)/treatment group/P62 T_3.7x.jpg]

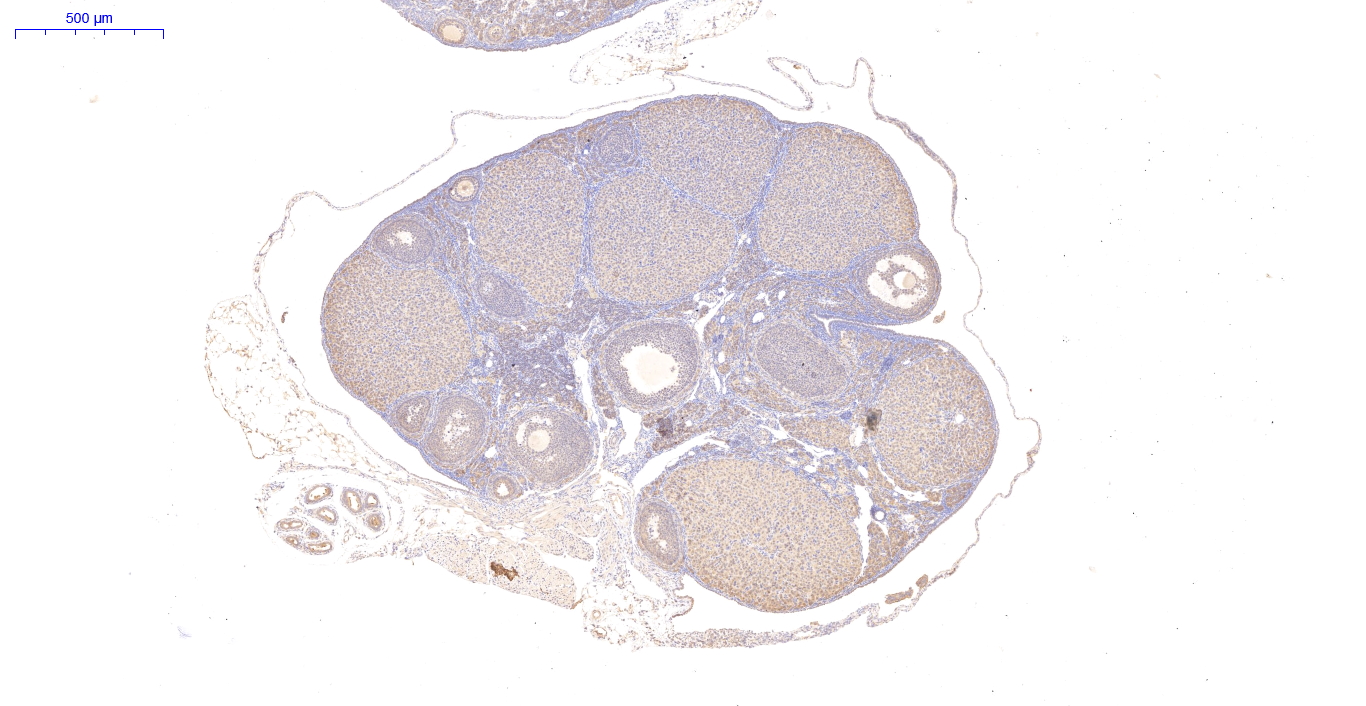

Supplement: Supplementary file 1 — Additional file 1. [file 13048_2021_854_MOESM1_ESM.zip › Supplementary Materials/Supplementary results of ovarian immunohistochemistry (whole ovarian section)/treatment group/SOD 1 T_3.7x.jpg]

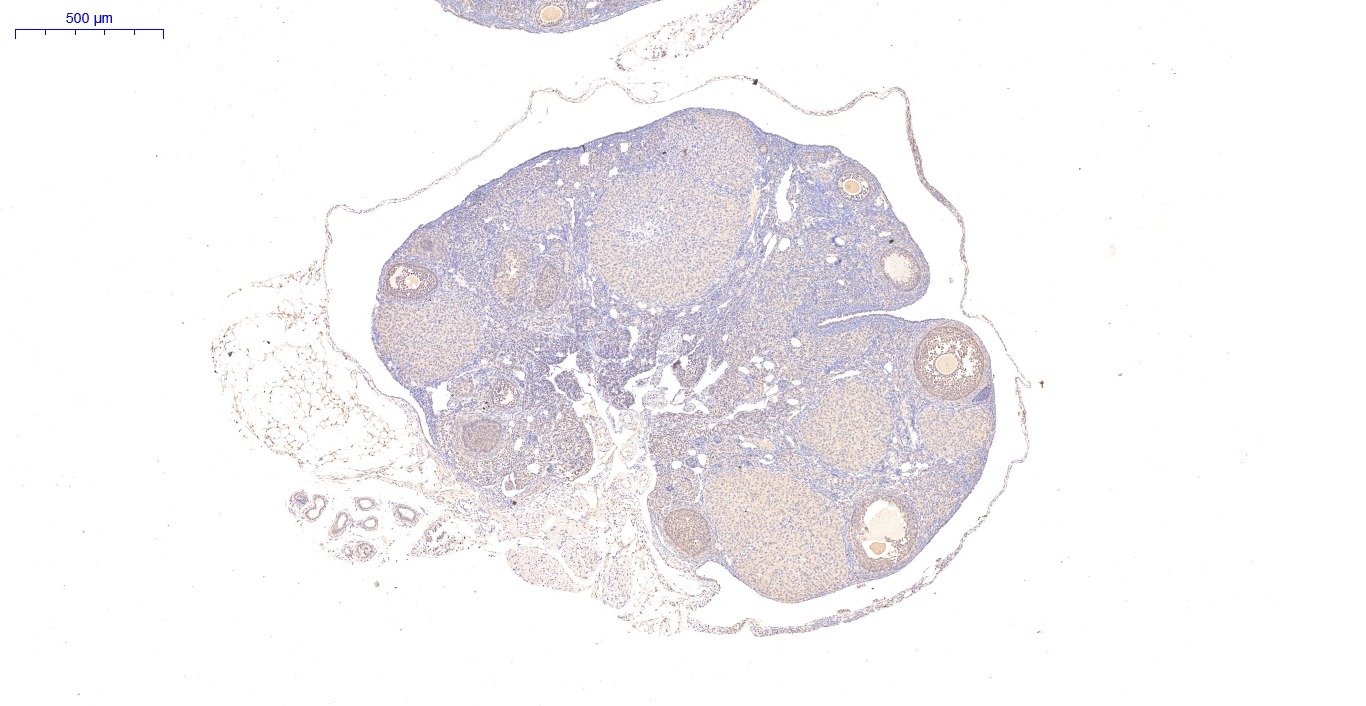

Supplement: Supplementary file 1 — Additional file 1. [file 13048_2021_854_MOESM1_ESM.zip › Supplementary Materials/Supplementary results of ovarian immunohistochemistry (whole ovarian section)/treatment group/sirt1 T_3.7x.jpg]

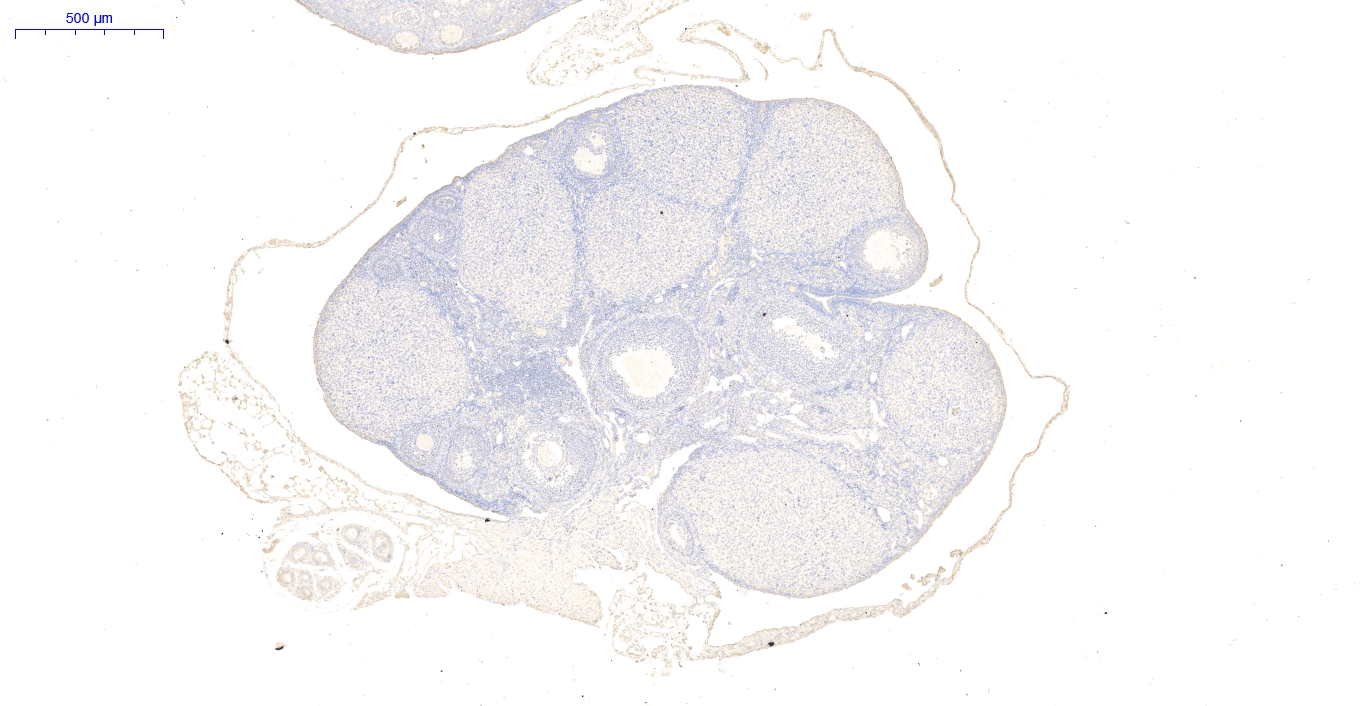

Supplement: Supplementary file 1 — Additional file 1. [file 13048_2021_854_MOESM1_ESM.zip › Supplementary Materials/Supplementary results of ovarian immunohistochemistry (whole ovarian section)/treatment group/sirt3_3.7x.jpg]
