# Supplementary material for: Effects and mechanisms of mUCMSCs on ovarian structure and function in naturally ageing C57 mice
Source: J Ovarian Res. 2021 Oct 13;14:133. doi: 10.1186/s13048-021-00854-5 (PMC8515706; doi:10.1186/s13048-021-00854-5)
Supplement: Supplementary file 1 — Additional file 1. [file 13048_2021_854_MOESM1_ESM.zip › Supplementary Materials/20210705Supplementary materials.doc]

**Supplementary materials**

**Preparation of mouse ovarian granulosa cells**

1) PMSG was injected at a concentration of 1 IU/µl into the intraperitoneal cavity of each mouse at 10 IU/mouse (i.e., 10 µl/mouse). The PMSG was diluted by 10 times with sterile water for injection, and 100 µl was injected per mouse via tail vein after dilution.

2) Forty-eight hours later, the mouse was killed by cervical dislocation. The ovaries and fallopian tubes were removed, placed in saline containing 1% penicillin, and then transferred to a sterile operating table.

3) The ovaries and fallopian tubes were washed twice with normal saline containing 1% penicillin-streptomycin and transferred to a new EP tube. The tissue was cut with scissors, and 10 times the volume of 0.1% type II collagenase was added. The tube was heated, and the contents were mixed well. The tissue was digested with shaking in a 37 °C incubator for 30 min.

4) A 70 µm cell strainer was placed on the mouth of a 50 ml centrifuge tube and used to filter the digested tissue suspension. An appropriate amount of pre-cooled PBS was added to the 50 ml centrifuge tube after filtration, and the mixture was centrifuged at 1000 rpm for 5 min. The supernatant was discarded. The pellet was washed with PBS once and then resuspended with 100 µl of PBS. The liquid was transferred to a 5 ml round-bottomed sorting test tube (polypropylene, locking cap).

5) A primary antibody against FSHR (2 µl) was added to the polypropylene flow tube as well as a blank tube. Then, 4 µl of FITC-labelled secondary antibody was added and mixed well. The tubes were incubated on ice for 30 min.

6) A volume of 300 µl of PBS was added to each polypropylene flow tube and mixed well. The tube contents were filtered twice with a 200-mesh gauze sheet and sorted with a cell sorter. The sorted cells were collected with a new EP tube.

7) Each aliquot of cells was centrifuged at 3000 rpm for 10 min. The remaining 50 µl of the upper layer of liquid was aspirated and discarded; 70 µl of Qiagen micro-lysis reagent was then added to each aliquot and mixed well. The liquid was transferred to a labelled cryotube and flash-frozen in liquid nitrogen. Each sample was stored at -80°C and sent on dry ice to Shanghai Ouyi Biomedical Technology Co., Ltd., for single-cell sequencing.

**Principal component analysis data**

Dark blue represents the young control group, light blue represents the model control group, and green represents the mUCMSC treatment group. Samples of the same group tended to cluster together.
